# Rooting plant awareness: development of a tool to explore human-plant relationships

**DOI:** 10.7717/peerj.21667

**Published:** 2026-09-08

**Authors:** Benno Dünser, Ivo Ponocny, Francisco Javier Navarro Prieto, Peter Lampert, Peter Pany

**Affiliations:** 1 Austrian Educational Competence Centre for Biology, University of Vienna, Vienna, Austria; 2 Faculty of Psychology, Sigmund Freud Private University Vienna, Vienna, Austria; 3 Department of Philosophy, Universidad Autónoma de Madrid, Madrid, Spain; 4 Department of Environmental and Life Sciences, Karlstad University, Karlstad, Sweden; 5 Department of Education in Secondary Schools, University of Teacher Education Vienna, Vienna, Austria; 6 Core Facility Botanical Garden, University of Vienna, Vienna, Austria

**Keywords:** Assessment, Plant awareness, Construct validation, Rating scale, Nature disconnection, Environmental education, Human-plant relations, Plant blindness

## Abstract

**Background:**

Although plants play a crucial role in ecosystems and human well-being, public awareness of their ecological significance remains limited. Furthermore, no validated construct or instrument currently exists to systematically assess plant awareness.

**Methods:**

This study presents an operationalisation and statistical validation of the plant awareness construct through a newly-developed assessment tool. Using confirmatory factor analyses (CFA) on data from 858 Austrian students across three school types and an expert group (botanists), this study is the first to validate plant awareness as a measurable psychological construct with distinct dimensions.

**Results:**

Results provide evidence for the proposed construct’s dimensionality and show internal consistency within its dimensions (attention, knowledge, attitudes). Comparing the dimensions, we found that attitudes towards plants were generally positive, knowledge varied depending on educational exposure, and attention tended to be low.

**Conclusion:**

Both the construct and the assessment tool offer initial insights into the structure of individual relationships with plants. By reliably distinguishing between individuals with varying levels of plant awareness, the tool demonstrates its applicability in research and education. The findings of this study provide a step towards a better understanding of human–plant relationships and pave the way for further research into connections with nature.

## Introduction

Plant awareness refers to the recognition, understanding, and valuation of plants’ ecological and societal roles (Dünser et al., 2024b; Parsley, Daigle & Sabel, 2022; Stagg & Dillon, 2022). It is foundational to sustainability, as plants underpin food systems, climate regulation, and biodiversity, and are connected to nearly every Sustainable Development Goal (Amprazis & Papadopoulou, 2025; Gosnell et al., 2025). However, cultural representations—from children’s literature (Schussler, 2008) and science textbooks (Link-Pérez et al., 2010) to journal covers (Fančovičová, Prokop & Kubíčková, 2022)—frequently undervalue plants, contributing to low public awareness of their complex functions (Sanders, 2007).

This limited understanding has tangible consequences: misperceptions reduce support for plant-focused conservation (Balding & Williams, 2016) and introduce aesthetic and geographic biases in resource allocation, favouring attractive, conspicuous, or broadly distributed species (Adamo et al., 2021; Adamo et al., 2022). Plant awareness, defined as attention to plants, understanding of their roles, and attitudes reflecting their value, is thus multidimensional. It shapes how people notice plants, interpret their functions, and prioritise their conservation and use (Gosnell et al., 2025).

Fostering plant awareness is both an educational and policy imperative. Higher awareness strengthens support for plant-inclusive conservation, reduces funding and research biases, and informs more effective climate and biodiversity policies (Adamo et al., 2021; Adamo et al., 2022; Balding & Williams, 2016; Bofferding & Kloser, 2015). By advancing plant awareness, societies can better recognize plants’ contributions to human well-being and translate that recognition into action, aligning with environmental and social objectives.

### Related theories and concepts for understanding plant awareness

Scientific knowledge becomes socially actionable only when it is supported by a public that actively engages with and applies it. Civic epistemologies—societal practices for validating and integrating scientific knowledge (Jasanoff, 2005)—help explain why (botanical) knowledge alone often fails to inform decisions (Knight et al., 2008; Kollmuss & Agyeman, 2002). In the case of plants, this “plant civic epistemology” is particularly weak: despite growing scientific understanding of plants’ ecological complexity, societal practices often fail to reflect this knowledge. For example, studies show that students from kindergarten through college are more capable of recalling and naming animals than plants (Patrick & Tunnicliffe, 2011; Schussler & Olzak, 2008) and exhibit a marked preference for studying animals over plants (Wandersee, 1986). This disconnect is further evidenced by the systematic neglect of plants in textbooks (Schussler et al., 2010; Uno, 2009) and laboratory work, where botanical content is frequently marginalised. Such patterns demonstrate how botanical knowledge, despite its scientific robustness, struggles to translate into educational priorities or public consciousness.

However, educational interventions offer promising pathways to bridge this gap. Nyberg & Sanders (2014) demonstrated that hands-on activities, such as having students grow pea plants and document their development, can foster both observational skills and emotional connections to plants. These examples align with Mach et al.’s (2020) call for the “coproduction of knowledge”, where researchers and the public collaborate to generate actionable insights. Such approaches decentralise knowledge production, ensuring that botanical science is not only understood but also applied in ways that address societal challenges like biodiversity loss and climate change.

However, in many Western contexts, weak public engagement with plant-related civic epistemologies limits the diffusion of plant awareness into education, policy, and everyday practice. While individual perceptual shifts (*e.g.*, philosophical reflection or experiential learning) can foster appreciation for plants, systemic change is equally critical (Kacprzyk, 2026). Institutional arrangements that legitimise and mobilise plant knowledge as a public good, such as equitable funding for botany education, inclusive policy frameworks, and cross-disciplinary collaborations, are necessary to counter cultural biases. Without such structures, the marginalisation of plants in civic discourse and sustainability efforts will persist, undermining the ethical foundations of conservation.

Furthermore, complementary perspectives on human–nature relations highlight mechanisms of disconnection at individual and societal levels. The “wheel of disconnection” (Beery et al., 2023) shows that limited personal experiences, sociocultural norms, and institutional barriers constrain nature connectedness and, by extension, plant awareness. Evidence from local contexts illustrates how gaps in botanical knowledge and insufficient institutional support can be countered through skills-building, community-based conservation, and other participatory approaches (Beckwith, Johansson & Huff, 2022). Co-production of knowledge among scientists, educators, and communities enhances relevance, legitimacy, and adoption compared to traditional one-way dissemination (Mach et al., 2020). Additionally, recent plant-focused citizen science demonstrates that co-produced, context-specific engagement can increase participation and facilitate shared knowledge creation in local contexts (Gibson et al. 2026).

The relationship between plant awareness and the broader construct of nature connectedness (Nisbet & Zelenski, 2013; Tam, 2013) reveals a hierarchical yet interdependent dynamic. Nature connectedness—often measured through validated scales such as the NR-6 (Nisbet & Zelenski, 2013) or Environmental Identity Scale (EID) (Clayton et al., 2021; Clayton & Opotow, 2003)—captures an individual’s cognitive, affective, and experiential bonds with the natural world. Tam’s (2013) empirical synthesis of connection-to-nature measures demonstrates strong convergence across all of these constructs. However, given that plants are frequently marginalised in cultural and educational contexts (Fančovičová, Prokop & Kubíčková, 2022; Link-Pérez et al., 2010; Schussler, 2008), plant awareness may serve as a specific mechanism for addressing broader disconnection, especially in fields where plants are consistently undervalued.

This individual-level construct is further expanded by the human-nature disconnect framework (Beery et al., 2023), which underscores that disconnection is not merely the absence of connection but an actively produced state through sociocultural, institutional, and political processes, including at both individual and societal levels. While plant awareness is assessed at the personal level—through one’s capacity to perceive, understand, and value plants—its development is profoundly shaped by the structural impediments identified in Beery et al.’s (2023) “wheel of disconnection.” Plant awareness illustrates how individuals become disconnected, often beginning with limited personal experience (Stagg & Dillon, 2022).

This disconnection is reinforced by cultural norms, such as the underrepresentation of plants in media (Fančovičová, Prokop & Kubíčková, 2022; Schussler, 2008) and by institutional biases, including the underfunding of plant conservation (Balding & Williams, 2016). Thus, mirroring Beery’s ideas, plant awareness exists at the intersection of personal cognition (nature connectedness) and societal context (human-nature disconnect): plants are underrepresented in children’s literature (Schussler et al., 2010), science curricula (Uno, 2009), and conservation campaigns (Albert, Luque & Courchamp, 2018; Mammola et al., 2020), which collectively perpetuate a cycle of disconnection.

Furthermore, plants are especially vulnerable in human-nature relationships because their static, silent, and often unnoticed roles in ecosystems and daily life attract less human attention. Unlike charismatic megafauna, plants lack sensory cues such as movement or vocalisations that typically engage people (Balas & Momsen, 2014; New, Cosmides & Tooby, 2007). As a result, they potentially may be more susceptible to extinction of experience (Soga & Gaston, 2016; Soga & Gaston, 2020) and shifting baseline syndrome (Soga & Gaston, 2018).

### Current measurement, limitations, and the need for improvement

Progress is hampered by the absence of a clearly defined, empirically validated construct and a corresponding measurement approach (Brkovic, Sanders & Nyberg, 2025). Terminology has shifted from “plant blindness” (Wandersee & Schussler, 2001) to “plant awareness disparity” (Parsley, 2020) and, more recently, “plant awareness” (Dünser et al., 2024b; Pany et al., 2022), reflecting a move away from pathologising perception toward a broader socio-cognitive framing (Albuquerque et al., 2025). Yet inconsistencies in definitions and dimensionality, and the lack of a comprehensive instrument, impede assessment across individual, regional, and cultural contexts (Brkovic, Sanders & Nyberg, 2025; Stagg, Hetherington & Dillon, 2024).

Existing tools typically capture fragments—such as attitudes or self-reported interest—without integrating multiple dimensions or linking measures across studies (Fančovičová & Prokop, 2010; Parsley, Daigle & Sabel, 2022). Concept clarity is crucial for cumulative science: well-specified constructs support coherent theorising, enable placement within a nomological network, and improve comparability across studies (Gerring, 2012; Green, 2014; Locke, 2003). Within this framework, a construct is a term that describes and specifies the unique attributes of a phenomenon (Lambert & Newman, 2023).

A pivotal contribution to developing the phenomenon into a construct came from Parsley (2020), who introduced plant awareness disparity (PAD) to address ableist connotations in the original terminology. Building on this, Parsley, Daigle & Sabel (2022) developed and validated the Plant Awareness Disparity Index (PAD-I), the first comprehensive self-report instrument to measure plant awareness (disparity) across four components: attention, attitude, knowledge, and relative interest. The instrument underwent rigorous development, including expert review, pilot testing with qualitative interviews, and two rounds of exploratory factor analysis (*N* = 1,062 and *N* = 553), resulting in a 25-item version, providing the first empirically validated tool to assess plant awareness and test educational interventions.

However, the PAD-I has faced critiques regarding its conceptual and methodological limitations. Parsley, Daigle & Sabel (2022) themselves noted that the instrument was developed primarily with undergraduate biology students in the US, a sample that was 74.3% female and 69.1% white, raising concerns about generalisability to more diverse populations. They also observed that, while the knowledge component improved significantly in their pilot study, attention and relative interest changed little, suggesting that knowledge-deficit models alone may be insufficient to address all aspects of plant awareness disparity.

These critiques were extended by demonstrating that students typically held positive attitudes toward plants, guided by evaluations based on aesthetic features and ecosystem services (Dünser et al., 2024a). However, interest in plants was notably low and specific to certain activities, clarifying that attitude and interest are conceptually distinct constructs (a differentiation sometimes unclear in existing literature; see Krapp & Prenzel, 2011) that require separate measurement. Furthermore, the deficit-oriented framing might inadvertently pathologise normal variations in plant awareness (Albuquerque et al., 2025) which underlines the need for a solution-oriented, strength-based approach that focuses on cultivating high plant awareness rather than merely measuring its absence.

**Figure 1:**
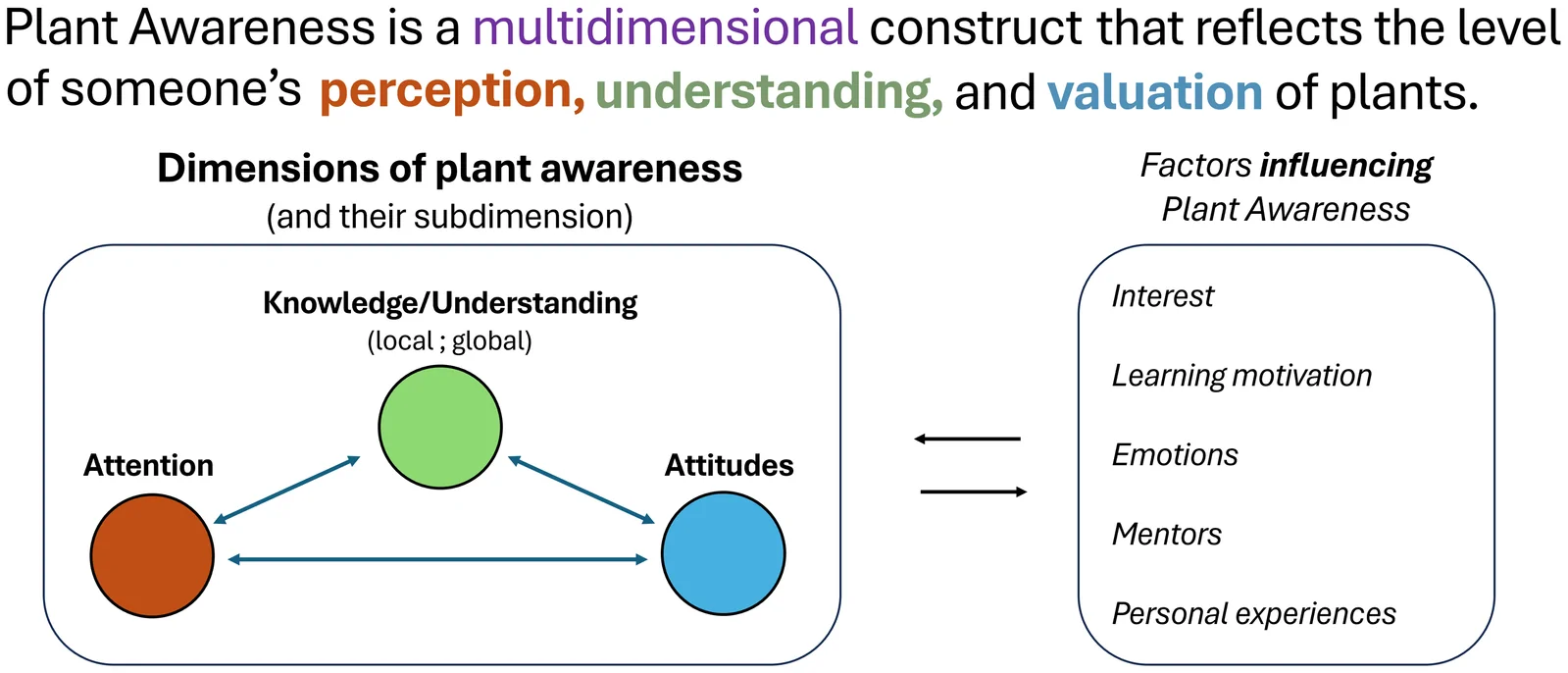
The plant awareness construct with its dimensions, as well as factors that affect plant awareness (adapted from Dünser et al., 2024b; p.1065).

After having explored the dimensions of plant awareness separately in several studies (Dünser et al., 2024b; Dünser et al., 2024a; Pany et al., 2022; Pany et al., 2026), in this paper, we present the statistical validation of the entire construct, which is the final step of construct development, following the three-step guideline of Lambert & Newman (2023): (1) definition and conceptualisation; (2) operationalisation; and (3) validation. The first step, definition and conceptualisation, has already been completed through a Delphi study that developed an expert-validated, theory-based definition of plant awareness (Dünser et al., 2024b). Experts defined plant awareness as a “multidimensional construct encompassing attention, understanding, and attitudes towards plants” (p. 1064). Variables such as interest in plants (Hidi & Renninger, 2006; Krapp, 2007), mentorship, and emotions, in contrast, were identified as factors affecting plant awareness (see Fig. 1).

A validated plant awareness tool, based on the consensus-based definition (Dünser et al., 2024b), can help identify how individual gaps in attention, understanding, or attitudes toward plants correspond to structural barriers described in the “wheel of disconnection” (Beery et al., 2023). For instance, low attention scores may be linked to sociocultural norms that make plants less visible in urban environments (Soga & Gaston, 2016), while limited understanding may reflect educational systems that prioritise animal biology over botany (Link-Pérez et al., 2010).

Furthermore, with a rigorous construct and validated tool, researchers and practitioners can assess individual plant awareness and, by extension, draw inferences about how societal, institutional, and educational systems foster or hinder it (Brkovic, Sanders & Nyberg, 2025). By situating plant awareness at the nexus of individual cognition (nature connectedness) and societal context (human-nature disconnect), researchers and practitioners can design targeted interventions—such as community-based conservation programs (Beckwith, Johansson & Huff, 2022) or plant-focused civic education—that address both personal and systemic dimensions of the human-plant relationship.

Standardised assessment thus supports targeted policy and educational design, helps correct biases in conservation attention (Adamo et al., 2022; Balding & Williams, 2016), and ultimately contributes to broader educational and societal transformation (Jasanoff, 2005). To address this measurement gap, this study advances the field from definition to application. Building on the Delphi-based construct definition, we translated the multidimensional conceptualisation of plant awareness into an assessment tool that can be used at the individual level and adapted for broader contexts (Dünser et al., 2024b).

Since we also seek to generate initial validity evidence to situate the construct within a broader theoretical framework, support comparability across studies, and inform interventions in education and policy, the present study pursues two objectives:

(1) Operationalise the plant awareness construct in a tool for assessing an individual’s plant awareness (point 2 on the checklist by Lambert & Newman, 2023).
(2) Obtain and analyse empirical evidence to take first steps for validating the plant awareness construct (point 3 on the checklist by Lambert & Newman, 2023).

## Materials & Methods

### The plant awareness assessment tool

In a first step, we used the checklist by Lambert & Newman (2023) to operationalise the plant awareness construct (see also DeVellis & Thorpe, 2021). This process generated selected items to effectively represent the dimensions of plant awareness (Dünser et al., 2024b). To link the items with the dimensions of plant awareness, we followed four key principles:

(1) Dimensionality: Each item was designed to specifically target one of the dimensions of plant awareness, ensuring a comprehensive and nuanced assessment and making possible the validation of the dimensions.
(2) Relevance in everyday life: We prioritised items that reflect the everyday experiences of respondents, focusing on common plant species, herbs, and basic ecological concepts. We conducted this study because previous research indicates low plant awareness, which we assessed using simple baseline items (*e.g.*, Pany et al., 2022).
(3) Empirical grounding: Many items were adapted from existing research in the field of plant awareness, leveraging the insights of previous studies (*e.g.*, Asshoff et al., 2019; Buck et al., 2019; Gladwell et al., 2012; Ryplova & Pokorny, 2020; Schunko & Brandner, 2022).
(4) Sustainability linkages: We aimed to connect plant awareness to broader sustainability issues by incorporating items related to relevant Sustainable Development Goals (SDGs). This approach aligns with the theoretical framework proposed by Amprazis & Papadopoulou (2020); Amprazis & Papadopoulou (2025). The complete item universe and item origins are shown in Table S1.

#### Scales of the plant awareness assessment tool

We developed one scale each for the three proposed dimensions of plant awareness: attention, knowledge, and attitudes. Scale development was informed by theoretical implications of DeVellis & Thorpe (2021) and used for the overwhelming part already published and peer reviewed structures. Scale values were computed as unweighted sum scores. All scales are designed in a way that higher scores indicate higher plant awareness. As effect size for group comparisons, the ordinal *η*^2^ was used (Cohen, 2008).

The operationalisation of the three dimensions—*attention*, *knowledge*, and *attitudes*—employs various tools that have already been tested and published in different papers. The tool for assessing attention is based on recollection from a picture (Pany et al., 2026). The knowledge subscales cover identification items already used in Pany et al. (2022), evapotranspiration items (Ryplova & Pokorny, 2020), and practical knowledge (Schunko & Brandner, 2022). Finally, the attitude items are taken from (Dünser et al., 2024a) who already validated and tested the reliability of the scale, as well as the evaluative valences underlying the individual items. Subsequently, all items were expert-validated by a group of 13 experts (six male, seven female; seven plant awareness researchers, six botany educators, two psychologists).

##### “Attention” scale.

Using the definition of the Delphi study, we understand attention towards plants within this paper as follows: “Attention towards plants encompasses the capacity to consciously perceive, recognise, and distinguish plants from their surroundings.” (Dünser et al., 2024b, p. 1064). Using this definition, attention can be understood as a selective cognitive mechanism (Koivisto, Kainulainen & Revonsuo, 2009; Lamme, 2003; Lamme, 2005) which enables the visual system to prioritise certain information (Carrasco, 2011). This is necessary as processing all incoming stimuli is impossible because processing is limited by the metabolic costs of neural activity (Rensink, 2014).

Different cognitive processes thus amplify relevant features and attenuate less relevant ones (Prinz, Müsseler & Rieger, 2017), representing an important evolutionary advantage. Working memory plays a central role in selective attention by temporarily holding and manipulating information during complex tasks (Hitch, Allen & Baddeley, 2024). Finally, attention is often influenced by past experiences, emotional associations, and stored knowledge in long-term memory (Collins & Curby, 2013; Ericsson & Kintsch, 1995). When specific values or objectives are introduced, attentional focus can be sustained for extended periods, even for low-priority stimuli (Wolfe & Horowitz, 2017).

For this first validation of the plant awareness construct, the attention dimension was operationalised as a recall task (see Schussler & Olzak, 2008) in the form of a free listing exercise after looking at an image of a typical Central European landscape (Pany et al., 2026). Researchers frequently use sketches or paintings in vision and attention studies because they allow precise control over visual stimuli while still conforming to established principles of ecological visual perception, such as face processing (Frischen, Eastwood & Smilek, 2008), spatial frequency statistics (Graham et al., 2012), and eye-movement patterns (Fuchs et al., 2011). The drawing was designed to represent a plausible Central European landscape using Austrian land-use data and species diversity information (*e.g.*, Ehrendorfer et al., 2022).

Species groups were included in roughly ecological proportions, while accounting for practical constraints such as the impossibility of depicting the true numerical dominance of insects or the small size of many organisms. Decisions, such as depicting more plant than animal species, limiting red tones due to common red-green weakness, and focusing on a spring/early-summer scene to maximise visual variety, were made based on perceptual and ecological reasoning. Expert discussions led to refinements, including additional flowering plants, fungi, and well-known animal species. A professional graphic designer then created the final digital drawing, following strict instructions to ensure accurate diagnostic features, relative sizes, and high recognizability. Items were positioned naturalistically, though proportional adjustments were necessary to enhance recognisability on a screen.

After looking at the drawing for a maximum of 60 s, students were asked to write a list of all objects they could remember. To assess attention towards plants, we focused on a group of plants that are relevant in students’ everyday life and, thereby, easier to recognise: useful plants (Krüger & Burmester, 2005). Therefore, we examined the frequency with which the ten most prominent useful plants depicted in the image, including the apple tree and the strawberry, appeared in participants’ free recall lists.

##### “Knowledge about plants” scale.

In operationalising the “knowledge about plants” dimension, we follow its definition as “(…) encompass(ing) an individual’s understanding of plants, their needs and life processes, their essential contributions to ecosystems, as well as their interconnectedness and interaction with most life, including humans and their culture.” (Dünser et al., 2024b, p. 1064). This broad conceptualisation is grounded in constructivist and conceptual-change perspectives, which view “understanding” as the organisation and restructuring of interconnected concepts rather than the accumulation of isolated facts (Amin, Levin & Levrini, 2023).

We acknowledge that the operationalisation of this dimension also includes factual knowledge and may therefore, at first glance, appear to function as a simple knowledge test rather than a measure of deeper conceptual grasp. However, several subscales require system-level reasoning (*e.g.*, carbon cycle, agriculture, sustainability) or draw on practical and culturally embedded knowledge, thereby exceeding simple recall. The coexistence of factual, conceptual, and practical elements reflects the assumption that plant awareness relies on a multifaceted knowledge base that integrates global biological principles with locally grounded experience. Additionally, we would like to emphasise that “knowledge” is an important intermediary component and key for influencing attitudes (Schlegel, Breuer & Rupf, 2015), which is the reason for the prominent role of knowledge-related items in the presented instrument.

The “knowledge about plants” scale evaluates students’ foundational knowledge about plants through seven subscales: “plant species identification” (*e.g.*, Buck et al., 2019; Kaasinen, 2019), “spice plants,” (Pany et al., 2022) “practical knowledge,” (Schunko & Brandner, 2022) “plants as living organisms” (Urry et al., 2017), “carbon cycle,” (Asshoff et al., 2019; Düsing, Asshoff & Hammann, 2019) “agriculture,” (Lampert et al., 2020) and “sustainability” (Dünser, 2022). These subscales were chosen as their content relates closely to students’ everyday experiences (Krüger & Burmester, 2005; Tunnicliffe & Reiss, 2000) or is fundamental for understanding the role of plants for ecosystems and humankind. To illustrate this, three selected subscales that are closest to students’ everyday life will be described in the following.

The “plant species identification” subscale, which represents local knowledge, requires students to identify five common mid-European plant species (*e.g.*, *Taraxacum officinale*) by selecting the correct image from four options or the correct plant name from a list of four. The “practical knowledge” subscale incorporates ethnobotanical views and practical knowledge into the assessment tool. An item battery by Schunko & Brandner (2022), which focuses on the naming—like Bermudez, Diaz & De Longhi (2018)—and a follow-up question about harvesting habits for Linden tree flowers (*Tilia* sp.) and blackberries (*Rubus* sp.), is used. The corresponding harvest item was also adapted and used for other identification items like dandelions (*Taraxacum officinale*). The “plants as living organisms” subscale, which represents global knowledge, measures the extent to which students classify plants as living organisms relative to animals, fungi, and bacteria. To achieve this, students evaluated eight life characteristics for each organism group (animals, plants, fungi, and bacteria) as defined by Urry et al. (2017).

##### “Attitude” scale.

In this article, we use the definition of attitudes towards plants as “evaluated responses towards plants, such as feeling comfortable or uncomfortable around plants.” (Dünser et al., 2024b, p. 1064; Karjalainen, Sarjala & Raitio, 2010). We define these responses as evaluation of an object (Eagly & Chaiken, 1993) as a valenced response to an entity (Ledgerwood, Eastwick & Smith, 2018). Although attitudes can be informed by cognitive, affective, and behavioural information (Maio & Haddock, 2007), our focus in this study is on capturing the overall evaluative orientation towards plants, rather than separately modelling its underlying components. Thus, our scale is not intended to represent the full tripartite model that informs attitudes, but to assess the outcome of these processes, namely, whether participants evaluate plants positively or negatively (in general). Therefore, we oriented our scale on prominent five-item scales that are often used to tackle similarly complex issues, like the “satisfaction of life scale” (Diener et al., 1985) that also uses rather general items. The index is not intended to replace comprehensive multidimensional attitude instruments, but rather to provide a concise, conceptually grounded measure of plant-related evaluations appropriate for large-scale surveys.

The current five-item attitude scale builds on the instrument developed by Dünser et al. (2024a), which builds on earlier attempts to measure attitudes towards plants by Fančovičová & Prokop (2010). Each of the five items targets a distinct evaluative component that is well documented in the literature. Two items assess students’ evaluative preferences for having more or fewer plants in (1) their school environment and (2) an urban setting, reflecting evidence on plants’ cognitive and emotional benefits, including improved attention and perceived restoration (Berto, 2005; Kuo, 2001; Laumann, Gärling & Stormark, 2003; Maller et al., 2009; Takayama et al., 2014; Taylor, Kuo & Sullivan, 2001). One item assesses perceived comfort when surrounded by plants, based on physiological evidence of calming effects (Gladwell et al., 2012; Karjalainen, Sarjala & Raitio, 2010; Park et al., 2011; Tsunetsugu et al., 2013). Another item targets aesthetic evaluation (“plants are beautiful”), given the acknowledged role of aesthetics in shaping positive attitudes (Dünser et al., 2024a; Hůla & Flegr, 2021; Nyberg, Brkovic & Sanders, 2021). The final item captures cultural valuation of plants within the family context (Berkes, 2017). Since all five items ask participants to provide an evaluative judgement, they align with the used definition of attitudes as valenced evaluations. The validation of the used items showed different important evaluative dimensions across all items, from aesthetic perception to emotional expressions and behavioural results of being close to plants (Dünser et al., 2024a).

#### Nomological network and differentiating between attitudes and interest

The distinction between attitudes and interest is subject to an ongoing debate in biology education (Krapp & Prenzel, 2011), as there are similar components that overlap in their expression (*e.g.*, emotions). In this paper, interest is understood as a motivational state (Hidi & Renninger, 2006) emerging from a dynamic interaction between the person and the object or stimulus (Krapp, 2007). The original mixed-methods research supporting the scale examined the distinction between attitudes and interests in detail, showing that the test items assess these constructs rather than focus on plants (Dünser et al., 2024a).

Qualitative analysis revealed that students provided fundamentally different types of reasoning for interest and attitude items: attitude explanations referred to evaluative considerations (*e.g.*, “plants are important for animals and humans because they produce oxygen”), whereas interest explanations referred to personal engagement, time investment, or social context (*e.g.*, “I find plants beautiful but I don’t deal with them in my spare time”). Quantitative analyses further supported this conceptual separation, showing generally positive attitudes but consistently low interest.

These findings indicated the need for a concise measure that captures evaluative stance rather than given engagement. The need (and chance) to separate attitudes from interest is also evident when applying the psychological definitions of interest and attitudes, as interest is a motivational state (Hidi & Renninger, 2006), while attitudes involve an evaluative process (Eagly & Chaiken, 1993). Against this background, the five-item attitude scale presented in this paper (see above) aims to provide a broad yet coherent measure of students’ general evaluative orientation towards plants. Finally, in order to explore the relation of plant awareness to other constructs, we assessed interest in plants, using the 14 plant related interest items of the ROSE-Questionnaire (Schreiner & Sjøberg, 2004) (*α* = 0.96), and nature connectedness combining the NR-6 (Nisbet & Zelenski, 2013) and the INS-scale (Kleespies et al., 2021), together consisting of seven items (*α* = 0.90).

#### Content validation

To ensure the content validity of the different items, we used think-aloud protocols. This method, commonly employed in piloting questionnaires, is particularly effective in identifying misleading task descriptions and unintended interpretations, ensuring definitional distinctiveness. The use of think-aloud protocols is intended to prevent the questionnaire items from being solved by alternative means rather than assessing the intended variable (Sandmann, 2014; Völzke, 2012).

Participants ranged in age from 10 to 18 years, as targeted. Given the age and gender differences reported in botanical education (*e.g.*, Kaasinen, 2019; Strgar, 2008), the sample included six female and six male students, with two think-aloud protocols conducted per tested age group. To ensure applicability across school types, piloting took place in a *Mittelschule* and a *Gymnasium* (see section “Sample” below). Moreover, participants with native languages other than German were included to verify that the items avoided jargon and complex phrasing, making them accessible and understandable.

Additionally, guided interviews were conducted. These interviews focused on clarifying challenging items, reviewing the understanding of specific keywords, and exploring the causes of difficulties encountered during questionnaire completion. When the think-aloud protocols did not reveal how students arrived at their conclusions, they were asked to explain their reasoning. Based on this thorough validation process, the items were revised to ensure that the assessment tool accurately assessed the desired understanding of plants. The combination of think-aloud protocols and guided interviews allowed us to align construct definitions with their operationalisation, to ensure content validity and to support interpretation of organisational and individual performance measures (Miller, Washburn & Glick, 2013).

Next, the complete assessment tool with items for all three dimensions was reviewed by 13 experts (seven female, six male) in plant awareness research (seven), botany education (four), and psychology (two), with expertise ranging from 5 to 20 years. All experts assessed each item’s alignment with its dimension, ensuring definitional distinctiveness (Colquitt et al., 2019) and confirming the general functionality of each item. This expert assessment of the items was part of the larger Delphi-study that also led to the definition of the plant awareness construct (see Dünser et al., 2024b). However, certain ambiguities stemming from expert-level understanding were addressed by simplifying the phrasing to avoid over-complication. The complete assessment tool along with a detailed rationale (Dünser, Navarro Prieto & Pany, 2024) is available online (Dünser, Navarro Prieto & Pany, 2024).

### Sample

In our study, we applied known-groups validity (Davidson, 2023) to our dataset by comparing the scores of students from three different school types (*Mittelschule*, *Gymnasium*, *Naturparkschule*) with a group of experts. This approach evaluated whether the assessment tool effectively distinguished between groups expected to differ in plant awareness, supporting its validity for this purpose.

The selected schools include three types of schools: urban “Mittelschule” (*n* = 66, 56% female, mean_Age_ = 12.77 ± 1.68, urban “Gymnasium” (*n* = 370, 51% female, mean_Age_ = 13.52 ±  2.33), and rural “Naturparkschulen” (*n* = 388, 43% female, mean_Age_ = 12.25 ±  1.26). *Mittelschulen* are lower secondary schools that provide pathways to vocational training or further academic studies after completing four school years. *Gymnasien* are schools that offer lower and upper secondary education and are characterised by higher academic expectations and a strong focus on preparing students for university. In rural areas, *Naturparkschulen* are a special type of lower secondary schools with a specific focus on environmental education and regional identity in their curriculum. *Naturparkschulen* align teaching approaches with the mission of a local “Nature Park” using it as a resource for outdoor learning.

These structural differences suggest that variations emerge across school types regarding the three dimensions of plant awareness, and the respective subscales. Such variations may stem from place-based education and differing curricular emphases; for example, *Naturparkschulen* attach more importance to environmental education. Based on these distinctions, we anticipate significant differences between *Mittelschulen* and *Gymnasien*, as well as between *Mittelschulen* and *Naturparkschulen*. However, due to their respective educational focus, we expect *Naturparkschulen* and *Gymnasien* to be more similar to one another.

In addition to testing the plant awareness assessment tool across these three school types, we have tested the tool among a group of plant experts. This inclusion of experts provides another layer for the known-groups comparison (Davidson, 2023). The expert group consisted of a group of 34 members of the Austrian Flora Research Association (http://www.flora-austria.at) (*n* = 34, 32% female, median_Age_ = 51–60 years), who participated voluntarily in our study after being invited *via* the newsletter of the Austrian Flora Research Association. We predict that students across all school types will exhibit lower plant awareness than experts.

The number of students participating was determined by availability at three schools, where teachers were willing to support data collection. Thus, the sample size reflects the practical constraints and opportunities of the study context, rather than being based on a priori statistical considerations. While this limits the potential to optimise sample size, it ensures ecological validity and aligns with the authentic conditions of educational research.

The “Guidelines for Good Scientific Practice” (Austrian Agency for Research Integrity, 2016) guided the research team. Before participation, students and experts were informed about the aims of the research, duration, procedure, and anonymity of the data. Participation was voluntary, and only those persons that gave written consent to participate in the study were included in the data analysis. Data were collected and analysed anonymously.

### Analytic strategy

In general, analyses were conducted using data from secondary 1 level students of all schools combined (*i.e.,* neither experts nor older pupils above fourth grade of Gymnasium). The reliabilities of the scales and subscales were computed as internal consistency *via* Cronbach’s *α*, based on tetrachoric correlations from the R-package psych (Revelle, 2020). The dimensionality of the dimension *knowledge* was further investigated by means of a Rasch analysis applied to the whole sample, using WinSteps (Linacre, 2023) calculating person and item reliabilities, IMNSQ infit and outfit statistics. The theoretically assumed dimensionality and latent structure were tested using descriptive correlations and confirmatory factor analyses (CFA), the latter computed *via* the R-package lavaan (Rosseel, 2012), using maximum likelihood estimation and tetrachoric correlations because of the dichotomous data format. Model fit was evaluated using the Tucker-Lewis-Index (TLI), the Comparative Fit Index (CFI), Akaike and Bayesian Information Criteria (AIC and BIC), as well as the Root Mean Square Error of Approximation (RMSEA). Finally, to validate assumptions about the dimensional structure, and the influence of sociodemographic variables, we applied linear regression.

Evaluations involving group comparisons (for known-group validation) were based on the complete data set. Due to some incomplete responses, the number of used cases can be smaller than the size of the corresponding sample. For distributional reasons, the comparisons were evaluated for significant outcomes *via* Kruskal-Wallis tests and pairwise Mann–Whitney tests for *post-hoc* analyses. Group differences served as indicators of both construct validity and the validity of the operationalisation, assessing whether comparisons between school types and experts revealed the theoretically expected differences in accordance with known-group comparison methodology (Davidson, 2023). Additionally, divergent outcomes in group comparisons between two scales indicated that they should remain separate.

### Ethics statement

According to the statute of the Ethics Committee of the University of Vienna (§2; see re-release of the statute section “Ethikkommission”, as per university gazette 2012-03-16, 18th piece, no. 106), the Ethics Committee evaluates research projects on or with humans: these are investigations which may affect the physical or psychological integrity, the sphere of privacy, other subjective rights or predominant interests of research participants. According to national laws (Austrian Universities Act 2002), the type of study presented in this article is exempt from mandatory formal ethical approval.

## Results

### Dimensionality of the plant awareness construct

The theoretical framework and dimensionality of plant awareness as well as interest imply clear expectations about the factorial structure (four-dimensional: attention, knowledge, attitudes, interest) which was tested by means of a confirmatory factor analysis (Table S2). The model fit indexes resulted in CFI (scaled value) = 0.906 (0.866), TLI = 0.903 (0.862), in spite of a significant *χ*^2^ = 12,269.745 (*df* = 4,088, *p* < 0.001), and an error of RMSEA = 0.065 (scaled values: 0.036), see Table S2 in the supplements for estimates, standard errors, and confidence intervals. Table 1 shows the intercorrelations between *attention*, *knowledge*, and *attitude*, which are small to moderate, though significant.

Reliability for all three dimensions showed sufficient to high consistency (see Table 2): Cronbach’s *α* resulted in acceptable values for attention and attitudes, and excellent ones for knowledge (Field, 2024; Nunnally & Bernstein, 1994). The reliability for the subscales measuring knowledge also ranged from sufficient to high, with values spanning from 0.73 for “practical knowledge” to 0.94 for “Categorizing plants as living organisms” (Table S3).

Rasch analysis confirmed the internal consistency of the *knowledge* dimension (across all groups). The analysis yielded a person reliability of 0.88, indicating sufficient consistency in the measurement of individual ability across the dataset. The item reliability with 0.99 demonstrated that the set of items is well-targeted to the participants’ abilities and sufficiently robust to replicate the findings in similar samples. Fit statistics supported the assumption of Rasch homogeneity: the IMNSQ infit for persons was 0.99, and the outfit was 1.06, while the IMNSQ infit for items was 0.99, and the outfit was 1.07. These values fall well within the acceptable range (0.7–1.3), confirming that both items and persons conform to the expectations of the Rasch model. No items or participants had to be excluded from the analysis due to misfit or extreme responses, reinforcing the validity of the dataset and the usability of the itemset for the sample.

### Known-groups comparison

**Figure 2:**
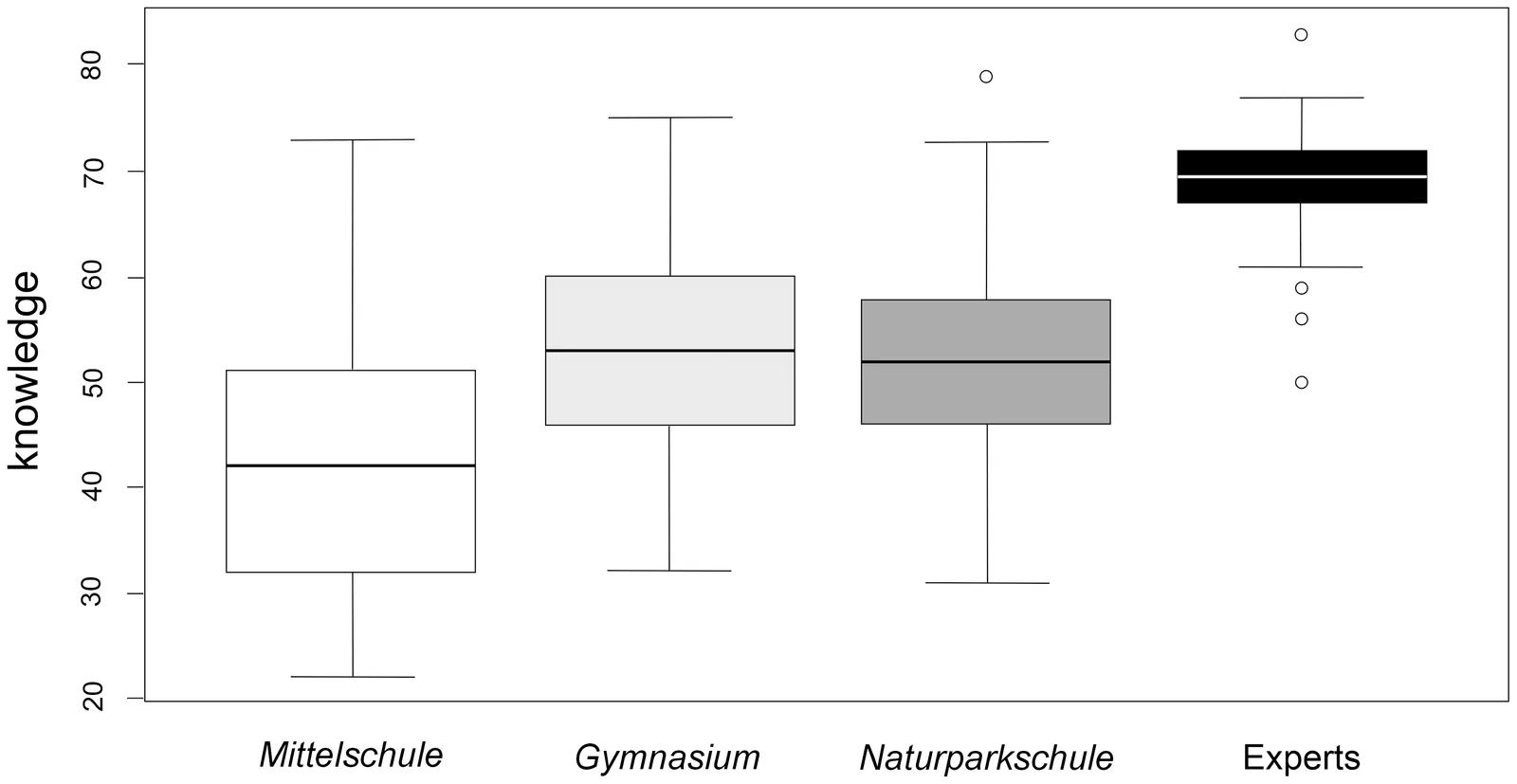
Boxplots showing differences in *knowledge aboutplants* between the four subsamples *Mittelschule, Gymnasium, Naturparkschule,* and experts.

For the dimension *attention* (values could range from a minimum of 0 to a maximum score of 10), we found a clear differentiation between students and experts, whereas students from the different types of school did not differ (see Table 3). Regarding *knowledge*, students from *Gymnasium* keep pace with those from *Naturparkschulen*, however, not those from *Mittelschule*. The experts stand out as the most homogeneous group with the smallest standard deviations. For the *knowledge* dimension (values could range from a minimum of 4 to a maximum score of 78), Kruskal–Wallis with *post hoc* analysis *via* pairwise Mann–Whitney tests (with Holm correction) found highly-significant differences between all school types and the expert group. Medium-level differences were observed between *Mittelschule* and other school forms, while only minimal differences emerged between *Gymnasium* and *Naturparkschulen* (see Table 3 and Fig. 2; for exact *p*-values see Table S4). Standard errors and confidence intervals are available in (Table S5). Students from *Naturparkschulen* outperformed or matched those from *Mittelschule* and *Gymnasium* in local and practical knowledge subscales. In contrast, *Gymnasium* students scored higher in global knowledge subscales. All Kruskal–Wallis tests for the *knowledge* subscales were highly significant (*p* < 0.001; exact *p*-values see Table S6) and are presented as boxplots in the (Figs. S1–S7).

**Table 1:** Scale intercorrelations with confidence intervals in brackets. Significant correlations are printed in bold, exact *p*-values are shown in the upper half of the table.

	Attention	Knowledge	Attitudes
Attention	–	**0.17** *p* = 1.60e−5	0.04 *p* = 0.3902
Knowledge	**0.17** [0.10; 0.25]	–	**0.28** *p* = 4.15e−11
Attitudes	0,04 [−0.05; 0.12]	**0.28** [0.20; 0.35]	–

**Table 2:** Reliability of the plant awareness dimensions (Cronbach’s *α*).

**Dimension**	**Cronbach’s *α***
Attention	0.78
Knowledge	0.96
Attitudes	0.77

**Table 3:** Means and standard deviations of the three dimensions of plant awareness (*attention*, *knowledge*, *attitudes*), interest, and nature connectedness, as well as effect sizes (ordinal *η*^2^) and *p*-values for the four subsamples *Mittelschule, Gymnasium, Naturparkschule,* and experts.

		*Attention*		*Knowledge*		*Attitudes*		Interest		Nature connectedness	
	N	Mean	SD	Mean	SD	Mean	SD	Mean	SD	Mean	SD
*Mittelschule*	66	0.59	0.84	36.09	11.5	19.29	3.67	18.82	10.28	24.75	6.65
*Gymnasium*	370	0.82	1.15	46.28	10.72	19.10	3.67	25.21	12.24	22.55	6.90
*Naturparkschule*	388	0.76	1.05	47.31	10.21	20.04	3.14	38.75	7.51	26.23	6.57
Experts	34	2.41	1.79	66.18	6.35	22.74	1.93	49.13	4.97	33.68	2.88
Kruskal Wallis test											
Ordinal *η*^2^ all groups		0.0611		0.1300		0.0428		0.289		0.112	
Ordinal *η*^2^ between schools		0		0.0472		0.00856		0.240		0.0554	
Ordinal *η*^2^ students *vs.* experts		0.0616		0.0878		0.0348		0.0737		0.0602	
*p*-value		2.45E−09		2.2E−16		5.07E−13		2.2E−16		2.2E−16	
H (*χ*^2^)		1.395		113.88		39.571		250.17		98.234	
df		3		3		3		3		3	

**Figure 3:**
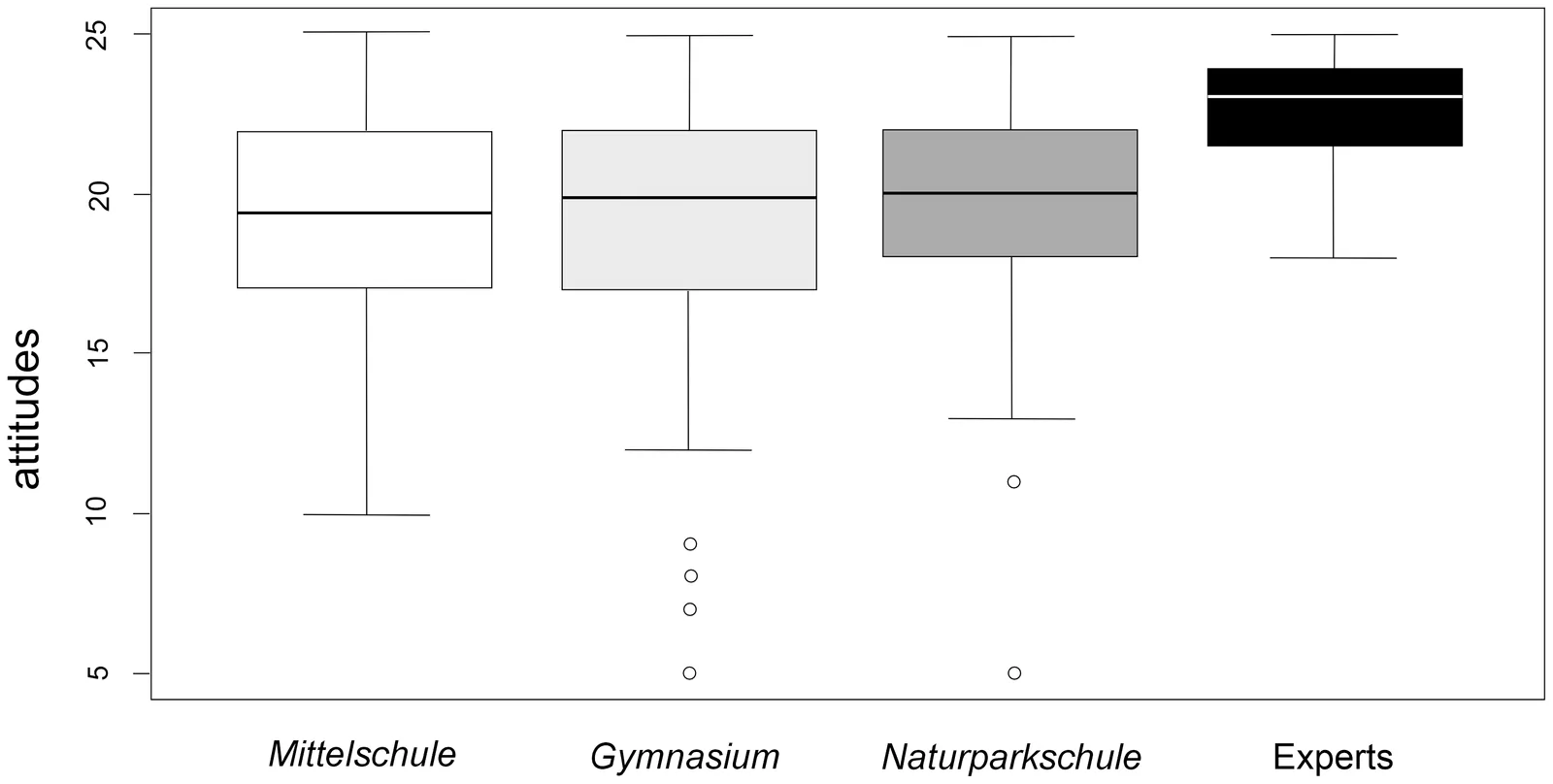
Boxplots showing differences in *attitudes towards plants* between the four subsamples *Mittelschule, Gymnasium, Naturparkschule,* and experts.

*Attitudes* towards plants were generally positive (values could range from a minimum of 5 to a maximum score of 25). *Post hoc* analysis using pairwise Mann–Whitney tests (with Holm correction) shows highly significant differences in *attitudes* between all students and experts. Significant differences are also found for all other pairs, except between *Mittelschule* and the other school forms. The mean comparison also reflects this, as experts’ attitudes towards plants are higher than those of students (see Table 3 & Fig. 3).

### Nomological network

The values for nature connectedness could range from a minimum of 0 to a maximum score of 42. Experts report the highest nature connectedness (33.68 ± 2.88), followed by *Naturparkschule* students (26.23 ± 6.57), *Mittelschule* students (24.75 ± 6.65), and *Gymnasium* students (22.55 ± 6.90). Experts not only score highest but also exhibit the least variability, while *Gymnasium* students show the lowest mean and the highest variability among the groups (see Table 3).

An open key question resulting from the Delphi study (Dünser et al., 2024b) was whether interest should be treated as a distinct variable or whether it should be merged with the attitude dimension. Mean comparisons show that while attitudes differ only slightly between students (ordinal *η*^2^ = 0.0428) interest differences between schools are very distinct (*η*^2^ = 0.289; see Table 3 & Fig. S8).

However, interest plays a significant role in explaining the relation of the plant awareness dimensions *attention*, *knowledge* and *attitude* with other constructs. For example, we predicted the scale *nature connectedness*, which consists of seven items such as “I like being outdoor in nature”, in a linear regression model by *attention*, *knowledge* and *attitude*, producing an adjusted *r*^2^ = 0.3263 (*p* = 2.24e−16, *F* = 75.27, df1=3, df2 = 457). Adding interest as a predictor, adjusted r^2^ was increased to 0.3666, with *p* = 2.2e−16, *F* = 67.57, df1 = 4, df2 = 456) and a highly significant coefficient of interest (0.12628, s.e. = 0.02302, *p* = 6.85e−08). This also means that the high explanatory value of interest as a single predictor (adjusted *r*^2^ = 0.1729, *p* = 2.2e−16, *F* = 97.19, df1 = 1, df2 = 459) collapsed substantially when *attention*, *knowledge* and *attitude* were also included.

### Influence of sociodemographic variables on plant awareness

The dimensions of plant awareness (*attention*, *knowledge*, *attitude*) are in part dependent on sociodemographic characteristics, according to a multiple linear regression with age, gender (dummy variable “male"), and type of school as predictors (with *Gymnasium* as anchor category). Regressions, confidence intervals and effect sizes can be found in Table S7. Differences from *Gymnasium* were only noted for *Mittelschule* in knowledge. Being male predicted less awareness in all variables except for *knowledge*. *Attitude* decreased with age, but *attention* increased.

## Discussion

Results show that plant awareness exists as a separate construct, including a robust definition, conceptualisation, and validation. Confirmatory factor analyses confirmed the proposed three-dimensional structure of the plant awareness construct based on the proposed definition (Dünser et al., 2024b). The model fit reached the cutoff scores (>0.90 for TLI or CFI, and RMSEA < 0.08). Higher cutoff scores are often applied (TLI or CFI > 0.95 or RMSEA < 0.06) based on Hu & Bentler (1998) who, however, rather recommend “close to” criteria instead of an absolute cutoff (p. 449). Due to the complexity of the construct, we consider the scores as a good basis for further refinement of the tool (see Groskurth, Bluemke & Lechner, 2023; Marsh, Hau & Wen, 2004) for a critical discussion of absolute benchmarks).

The three dimensions are distinctly separated (see Table S2) yet remain correlated (see Table 1), which supports the proposed dimensionality of the construct. *Attitudes* and *attention* show no correlation, which may be explained by the low number of items for *attitude* and the differing item difficulties of the *attention* scale (which consists of rather difficult items) and the *attitude* scale (which consists of rather easy items). The correlation of *knowledge* with both other dimensions underlines the importance of environmental education as a door opener to foster plant awareness (Mach et al., 2020).

Based on observed differences, we propose a hierarchical structure in which attitudes towards plants form a universal and foundational baseline, as reaffirmed by Dünser et al. (2024a). The assessed attitudes are positive and consistent across students and experts (see Table 3) regardless of predictors. *Knowledge*, by contrast, is more exclusive, influenced by multiple factors, and displaying broader variability. *Attention*—at least evaluated as a recall free-list task—emerges as the mostadvanced and challenging dimension to achieve.

Given the theoretical distinction between attitudes and interest, the data provide cautious support for treating interest as a related but distinct dimension, in line with previous work (Dünser et al., 2024b; Dünser et al., 2024a). The confirmatory factor analysis shows that interest loads on a separate factor (see Table S2), which is consistent with the conceptual differentiation between attitude and interest outlined in the literature (*e.g.*, Eagly & Chaiken, 1993; Hidi & Renninger, 2006; Krapp, 2007).

At the same time, the correlations between interest and the three dimensions of plant awareness indicate that the constructs are not completely independent. The linear regression further suggests that the direct effect of interest on nature connectedness decreases substantially once its influence is mediated through these three dimensions, supporting its role as an intervening variable rather than a standalone core dimension of plant awareness.

Further empirical evidence for treating attitudes and interest as different constructs is given when comparing the different school types in our samples: attitudes show only very minor differences (see Fig. 2), whereas interest shows a strong discriminative effect (see Fig. S8). This is not consistent with the assumption that both variables lie on a common latent scale. In that case, school differences would occur on both variables or none. Overall, we interpret these findings as tentative empirical support for separating interest from attitudes.

Known-group comparison (Davidson, 2023) further proves the validity and reliability of the tool as it was possible to differentiate between the groups in the prognosed way: experts’ values were the highest in all three scales representing the three dimensions of plant awareness followed by *Naturparkschule* and *Gymnasium* with *Mittelschule* as worst performing (see Fig. 2 and Table 3). As expected, students from *Naturparkschulen* surpass students from *Mittelschule* and *Gymnasium* or equal them in the local knowledge subscales (plant identification, spice plants) and the practical knowledge subscale, while students from *Gymnasium* score higher in the global knowledge subscales (agriculture, living organisms, sustainability, carbon cycle; see Table S6). The Rasch analyses provide robust evidence for the internal consistency and one-dimensionality of the *knowledge* dimension. Across diverse groups, the fit statistics, person reliability, and item reliability of the Rasch metrics demonstrated that the instrument performs well in measuring the intended construct among the intended participants and can be used across multiple age groups. This highlights the minimal ambiguity and understandability of items, as the assessment tool could be used in all school forms and ages in Austria producing reliable data regardless of upbringing, age or language spoken at home. No items or participants exhibited significant misfit, further reinforcing the validity of the testing tool for subsequent analyses.

By integrating CFA and linear regression, this study presents a first validation of the plant awareness construct and a theory-driven assessment tool that can advance research and educational practice. Our results prove that the operationalisation of the items depict the construct and the separation of the corresponding dimension well. The validity of the tool was supported by CFA. Dimensions themselves showed strong scale consistency in terms of Cronbach’s *α* (Field, 2024), reflecting high item fit. Across diverse groups, psychometric values demonstrated that the instrument performs well in measuring the intended construct within the aimed participants.

While the dimensions of plant awareness are now well-defined, the predictors fail to coherently explain variation among participants within the construct. Linear regression showed the impact of different factors regarding different dimensions and subscales, although their impact was not as prominent as assumed. We neither found clarity by comparing our findings to the already ambiguous findings of previous literature about the dimensionality and direction of the impact of the assessed socio-economic predictors. Amprazis, Papadopoulou & Malandrakis (2019) found differences in the naming of plants (in our conceptualisation a subcategory of the dimension *knowledge*) between male and female students. In our study, being male consistently predicted lower *attention* and *attitudes* but not *knowledge*, suggesting that gender differences in plant awareness may be linked to social or cultural influences, as is known from other educational contexts and variables, (*e.g.*, Baram-Tsabari & Yarden, 2011; Strgar, 2008).

We assume that stronger factors underlie the assessed predictors. The impact of different cultures, regions, and local transfer of knowledge and values regarding organisms may be even stronger than anticipated. We adopt an ethnobotanical perspective that views knowledge as a system interconnected with understanding, values, beliefs, and worldviews (defined by Gosnell et al., 2025). We argue that vertical knowledge transfer—passing knowledge from one generation to the next—may be a particularly significant factor. To better understand this process, we propose shifting focus from broad sociocultural assessments. Instead, we suggest examining the roles played within the parental generation and the extent to which both values and knowledge are transmitted (Stagg, Hetherington & Dillon, 2024).

### Limitations of the study

This study establishes an initial framework for assessing plant awareness. While linear regression clarified the relationship between plant awareness and interest, nature connectedness provides a limited example of the additional explanatory value of interest beyond plant awareness, as it primarily involves attitude rather than attention and knowledge. In further research, other reference constructs, like *willingness to protect the environment* (Abdul-Wahab, 2008) or *willingness to act against climate change* (Xie et al., 2019), should be tested to obtain a better understanding of the plant awareness nomological network.

The presented assessment tool is specifically adapted to the flora and ecological context of northern and central Europe, particularly Austria, and includes locally relevant plant, animal, and fungal species, especially in the plant identification subscale and the attention assessment tool. When using this tool outside Europe, adaptations would be necessary to replace the depicted species and landscape elements with those representative of the target region’s native biodiversity, land-use patterns, and cultural significance. Additionally, the seasonal and geographical context (*e.g.*, spring/summer in Austria) would need to be adjusted to align with local ecological conditions and the familiarity of participants with regional species. Without such modifications, the tool’s validity and reliability in assessing plant awareness could be compromised.

The scales presented in the tool provide a first foundation for assessing the different dimensions of plant awareness. However, there is room for improvement. Further development and refinement of the scales is important to solidify the tool, but also the validity of the construct. For example, the assessment tool could be enhanced by a more differentiated scale for attitudes towards plants, focusing on cognitive and affective factors.

Simultaneously, a comparison of how different assessment tools influence the construct would be interesting. For the *attention* dimension, we used a free-listing recall task, which indirectly assesses attention. Thus, in the next step, a comparison between our free-listing recall task and a direct attention assessment, for instance, presented by Sanders, Nyberg & Brkovic (2024), would improve our grasp of how the assessment tool may impact the correlation between the different dimensions of plant awareness. Finally, it would provide insight into whether attention, in our validation, the most advanced dimension of plant awareness, shifts in difficulty depending on the testing method.

## Conclusions

By enabling the assessment of an individual’s plant awareness, the study presented here allows conclusions to be drawn about how societal, institutional, and educational systems foster plant awareness. Following the suggestion of Beery et al. (2023), we adopted a mixed-methods approach to contribute to addressing humans’ disconnection from nature. Specifically, our findings regarding the plant awareness of individuals in Austria highlight the need to engage not only at the individual level but also from a societal standpoint. With a rigorous construct and validated tool (Brkovic, Sanders & Nyberg, 2025), we can now assess and compare plant awareness across different contexts, paving the way for educational and societal transformation (Kacprzyk, 2026).

The introduction of the assessment tool opens new opportunities that were previously unavailable (Stagg, Hetherington & Dillon, 2024). Although various educational interventions have been implemented, each relied on its own assessment method without a clear underlying dimensional framework, making comparison difficult (Brkovic, Sanders & Nyberg, 2025). The new tool facilitates the assessment and comparison of intervention impacts. Enhanced understanding of the framework supports the strategic selection and design of targeted approaches, streamlining the desired changes in plant awareness and relationships with plants (Beckwith, Johansson & Huff, 2022). Furthermore, the tool can be used to provide insights into national differences, allowing comparison of both baseline data and predictors. This step is crucial for understanding how educational systems influence plant awareness and, more importantly, for identifying underlying factors (*e.g.*, integration of plants into everyday-life, mode of knowledge transfer), which can help establish effective actions to foster plant awareness.

Moving forward, integrating the tool into diverse educational and civic settings can bridge the gap between scientific knowledge and society (Jasanoff, 2005). As highlighted in the theoretical background, civic epistemologies emphasise that scientific knowledge only becomes actionable when sustained by public engagement and culturally specific ways of knowing. Therefore, future research should examine how different knowledge transfer methods—from formal education to community-based interventions—shape plant awareness and contribute to the development of robust plant-related civic epistemologies. This aligns with Mach et al. (2020) who call for coproduction of knowledge, where researchers and communities collaborate to generate insights that are both scientifically rigorous and socially relevant. Expanding the tool’s application across diverse contexts can enhance engagement and strengthen the societal integration of botanical knowledge, directly addressing the limited public engagement that currently characterises plant-related civic epistemologies.

Subsequently, the tool could be expanded to explore local understanding and attention-related aspects tied to regionality across different parts of the world, reflecting the multidimensional nature of plant awareness. Furthermore, its scope can extend beyond students to include a broader range of participants, from policymakers to community groups. While plant awareness has primarily been studied within educational settings, this tool enables a more comprehensive approach, allowing for an examination across society. Such expansion would not only address individual-level gaps but also structural barriers identified in the wheel of disconnection (Beery et al., 2023), thereby fostering both personal and systemic changes in how societies value and engage with plants.

Finally, when focusing on the biodiversity crisis, plants are not the only overlooked organisms. The vast amount of literature regarding plant awareness is an excellent basis to broaden the construct. This newly-built basis could be useful for addressing many other cultural biases involving other groups of organisms (*e.g.*, insects, fungi). A clearer picture of how society regards plants opens the chance to now explore how plant awareness affects behaviours and values, which is crucial for tackling the biodiversity and climate crises through tailored educational interventions and to find answers how to lead society back to a closer connection to living organisms.

##  Supplemental Information

10.7717/peerj.21667/supp-1 Supplemental Information 1 Raw data with description of variables for all four subsamples of the study (English translation)

10.7717/peerj.21667/supp-2 Supplemental Information 2 Raw data with description of variables for all four subsamples of the study (German original)

10.7717/peerj.21667/supp-3 Supplemental Information 3 Codebook for the raw dataset

10.7717/peerj.21667/supp-4 Supplemental Information 4 Boxplots presenting the differences between the four subsamples *Mittelschule (Wr. NMS), Gymnasium (Wr. AHS), Naturparkschule,* and experts, regarding the *sustainability* subscale of the knowledge scale

10.7717/peerj.21667/supp-5 Supplemental Information 5 Boxplots presenting the differences between the four subsamples *Mittelschule (Wr. NMS), Gymnasium (Wr. AHS), Naturparkschule,* and experts, regarding the* spice plants* subscale of the knowledge scale

10.7717/peerj.21667/supp-6 Supplemental Information 6 Boxplots presenting the differences between the four subsamples *Mittelschule (Wr. NMS), Gymnasium (Wr. AHS), Naturparkschule,* and experts, regarding the *practical knowledge* subscale of the knowledge scale Since this scale also tests frequency of collecting plants, students from *Naturparkschulen* who live close to a nature park collect plants more often than experts who are rather located in an urban area

10.7717/peerj.21667/supp-7 Supplemental Information 7 Boxplots presenting the differences between the four subsamples *Mittelschule (Wr. NMS), Gymnasium (Wr. AHS), Naturparkschule,* and experts, regarding the *living organisms* subscale of the knowledge scale

10.7717/peerj.21667/supp-8 Supplemental Information 8 Boxplots presenting the differences between the four subsamples *Mittelschule (Wr. NMS), Gymnasium (Wr. AHS), Naturparkschule,* and experts, regarding the *plant identification* subscale of the knowledge scale

10.7717/peerj.21667/supp-9 Supplemental Information 9 Boxplots presenting the differences between the four subsamples *Mittelschule (Wr. NMS), Gymnasium (Wr. AHS), Naturparkschule,* and experts, regarding the *carbon cycle* subscale of the knowledge scale

10.7717/peerj.21667/supp-10 Supplemental Information 10 Boxplots presenting the differences between the four subsamples *Mittelschule (Wr. NMS), Gymnasium (Wr. AHS), Naturparkschule,* and experts, regarding the *agriculture* subscale of the knowledge scale

10.7717/peerj.21667/supp-11 Supplemental Information 11 Boxplots showing differences in interest for plants between the four subsamples *Mittelschule (Wr. NMS), Gymnasium (Wr. AHS), Naturparkschule,* and experts

10.7717/peerj.21667/supp-12 Supplemental Information 12 The item universe, showing the origin and genesis of the subscales, along with their relationship to the SDGs and their distribution within the dimensions

10.7717/peerj.21667/supp-13 Supplemental Information 13 Results of the confirmatory factor analysis for the dimensions of plant awareness and interest: estimates, standard errors, and confidence intervals. *N* = 474

10.7717/peerj.21667/supp-14 Supplemental Information 14 Reliability of knowledge and its subscales (Cronbach’s *α*)

10.7717/peerj.21667/supp-15 Supplemental Information 15 Exact p-values for the *post hoc* analysis via pairwise Mann-Whitney tests (with Holm correction) of the mean comparison of the three dimensions of plant awareness *attention*, *knowledge*, and *attitude*, as well as interest between the four subsamples

10.7717/peerj.21667/supp-16 Supplemental Information 16 Means, standard deviations, standard errors, and confidence intervals of the three dimensions of plant awareness (*attention*, *knowledge*, *attitudes*), as well as interest for the four subsamples * Mittelschule, Gymnasium, Naturparkschule,* and experts

10.7717/peerj.21667/supp-17 Supplemental Information 17 Means and standard deviations of the seven subscales of the plant awareness dimension *knowledge* effect sizes (ordinal *η*^2^) and p-values for the four subsamples *Mittelschule, Gymnasium, Naturparkschule,* and experts

10.7717/peerj.21667/supp-18 Supplemental Information 18 Regressions with confidence intervals and effect sizes

10.7717/peerj.21667/supp-19 Supplemental Information 19 Plant awareness inventory items (original language - German)

10.7717/peerj.21667/supp-20 Supplemental Information 20 Plant awareness inventory items (English translation)

## Additional Information and Declarations

### Data Availability

The following information was supplied regarding data availability: The raw measurements are available in Supplementary Files.

## References

[ref-1] Abdul-Wahab SA (2008). A preliminary investigation into the environmental awareness of the Omani public and their willingness to protect the environment. American Journal of Environmental Sciences.

[ref-2] Adamo M, Chialva M, Calevo J, Bertoni F, Dixon K, Mammola S (2021). Plant scientists’ research attention is skewed towards colourful, conspicuous and broadly distributed flowers. Nature Plants.

[ref-3] Adamo M, Sousa R, Wipf S, Correia RA, Lumia A, Mucciarelli M, Mammola S (2022). Dimension and impact of biases in funding for species and habitat conservation. Biological Conservation.

[ref-4] Albert C, Luque GM, Courchamp F (2018). The twenty most charismatic species. PLOS ONE.

[ref-5] Albuquerque UP, Cantalice AS, Oliveira ES, Santos FIR, Santos HC, Lima I da S, Silva JVM, Elias L, Albuquerque-Silva MM, Santana MVA de, Mata PT, Santos RKS dos, Brito Júnior V de M, Santos YAC (2025). Reframing botanical blindness and imperception through evolutionary lenses. Acta Botanica Brasilica.

[ref-6] Amin TG, Levin M, Levrini O, Taşar MF, Heron PRL (2023). Theorizing concept learning in physics education research: progress and prospects. The international handbook of physics education research: learning physics.

[ref-7] Amprazis A, Papadopoulou P (2020). Plant blindness: a faddish research interest or a substantive impediment to achieve sustainable development goals?. Environmental Education Research.

[ref-8] Amprazis A, Papadopoulou P (2025). Key competencies in education for sustainable development: a valuable framework for enhancing plant awareness. Plants, People, Planet.

[ref-9] Amprazis A, Papadopoulou P, Malandrakis G (2019). Plant blindness and children’s recognition of plants as living things: a research in the primary schools context. Journal of Biological Education.

[ref-10] Asshoff R, Düsing K, Winkelmann T, Hammann M (2019). Considering the levels of biological organisation when teaching carbon flows in a terrestrial ecosystem. Journal of Biological Education.

[ref-11] Austrian Agency for Research Integrity (2016). Guidelines for good scientific practice.

[ref-12] Balas B, Momsen JL (2014). Attention blinks differently for plants and animals. CBE—Life Sciences Education.

[ref-13] Balding M, Williams KJH (2016). Plant blindness and the implications for plant conservation. Conservation Biology.

[ref-14] Baram-Tsabari A, Yarden A (2011). Quantifying the gender gap in science interests. International Journal of Science and Mathematics Education.

[ref-15] Beckwith BR, Johansson EM, Huff VJ (2022). Connecting people, plants and place: a native plant society’s journey towards a community of practice. People and Nature.

[ref-16] Beery T, Stahl Olafsson A, Gentin S, Maurer M, Stålhammar S, Albert C, Bieling C, Buijs A, Fagerholm N, Garcia-Martin M, Plieninger T, Raymond MC (2023). Disconnection from nature: expanding our understanding of human–nature relations. People and Nature.

[ref-17] Berkes F (2017). Sacred ecology.

[ref-18] Bermudez GMA, Diaz S, De Longhi AL (2018). Native plant naming by high-school students of different socioeconomic status: implications for botany education. International Journal of Science Education.

[ref-19] Berto R (2005). Exposure to restorative environments helps restore attentional capacity. Journal of Environmental Psychology.

[ref-20] Bofferding L, Kloser M (2015). Middle and high school students’ conceptions of climate change mitigation and adaptation strategies. Environmental Education Research.

[ref-21] Brkovic I, Sanders D, Nyberg E (2025). Investigating plant awareness: methodologies, challenges and possibilities. Plants, People, Planet.

[ref-22] Buck T, Bruchmann I, Zumstein P, Drees C (2019). Just a small bunch of flowers: the botanical knowledge of students and the positive effects of courses in plant identification at German universities. PeerJ.

[ref-23] Carrasco M (2011). Visual attention: the past 25 years. Vision Research.

[ref-24] Clayton S, Czellar S, Nartova-Bochaver S, Skibins JC, Salazar G, Tseng Y-C, Irkhin B, Monge-Rodriguez FS (2021). Cross-cultural validation of a revised environmental identity scale. Sustainability.

[ref-25] Clayton S, Opotow S (2003). Identity and the natural environment: the psychological significance of nature.

[ref-26] Cohen BH (2008). Explaining psychological statistics.

[ref-27] Collins JA, Curby KM (2013). Conceptual knowledge attenuates viewpoint dependency in visual object recognition. Visual Cognition.

[ref-28] Colquitt JA, Sabey TB, Rodell JB, Hill ET (2019). Content validation guidelines: evaluation criteria for definitional correspondence and definitional distinctiveness. Journal of Applied Psychology.

[ref-29] Davidson M, Maggino F (2023). Known-groups validity. Encyclopedia of quality of life and well-being research.

[ref-30] DeVellis RF, Thorpe CT (2021). Scale development: theory and applications.

[ref-31] Diener E, Emmons RA, Larsen RJ, Griffin S (1985). The satisfaction with life scale. Journal of Personality Assessment.

[ref-32] Dünser B (2022). Pilotierung eines Fragebogens zur Quantifizierung von Plant Blindness. Master thesis.

[ref-33] Dünser B, Möller A, Anđić B, Lampert P, Bergmann-Gering A, Pany P (2024b). (Re) growing plant awareness: a Delphi study. Plants, People, Planet.

[ref-34] Dünser B, Möller A, Fondriest V, Boeckle M, Lampert P, Pany P (2024a). Attitudes towards plants –exploring the role of plants’ ecosystem services. Journal of Biological Education.

[ref-35] Dünser B, Navarro Prieto F, Pany P (2024). The plant awareness inventory.

[ref-36] Düsing K, Asshoff R, Hammann M (2019). Students’ conceptions of the carbon cycle: identifying and interrelating components of the carbon cycle and tracing carbon atoms across the levels of biological organisation. Journal of Biological Education.

[ref-37] Eagly AH, Chaiken S (1993). The psychology of attitudes.

[ref-38] Ehrendorfer LE, Niklfeld H, Schröck C, Stöhr O, Gilli C, Sonnleitner M, Adler W, Barta T, Beiser A, Berg C (2022). Red List of Ferns and Flowering Plants of Austria. Upper Austria.

[ref-39] Ericsson KA, Kintsch W (1995). Long-term working memory. Psychological Review.

[ref-40] Fančovičová J, Prokop P (2010). Development and initial psychometric assessment of the plant attitude questionnaire. Journal of Science Education and Technology.

[ref-41] Fančovičová J, Prokop P, Kubíčková M (2022). The effect of aposematic signals of plants on students’ perception and willingness to protect them. Sustainability.

[ref-42] Field A (2024). Discovering statistics using IBM SPSS statistics.

[ref-43] Frischen A, Eastwood JD, Smilek D (2008). Visual search for faces with emotional expressions. Psychological Bulletin.

[ref-44] Fuchs I, Ansorge U, Redies C, Leder H (2011). Salience in paintings: bottom-up influences on eye fixations. Cognitive Computation.

[ref-45] Gerring J (2012). Social science methodology: a unified framework.

[ref-46] Gibson K, Suškevičs M, Půse B, Barberis M, Cousins SAO, Fišer Ž, Julien M, Kivastik M, Klisz M, Lengyel A, Münzbergová Z, Puchałka R, Reinula I, Rienks F, Sheard JK, Siverin V, Stojanova B, Träger S, Aavik T (2026). Engaging the public in plant science: communication facilitators and barriers of scaling up a citizen science campaign. People and Nature.

[ref-47] Gladwell VF, Brown DK, Barton JL, Tarvainen MP, Kuoppa P, Pretty J, Suddaby JM, Sandercock GRH (2012). The effects of views of nature on autonomic control. European Journal of Applied Physiology.

[ref-48] Gosnell H, Reyes García V, Zinngrebe Y, Almeida Magris R, Benessaiah K, Bonilla-Moheno M, Chandipo R, Claudet J, Gemmill-Herren B, Goldstein B, Huntjens P, Ifejika Speranza C, Nakao F, Pandit R, Bosch Pereira L, Raab K, Soares T, Tittonell P, Guo X, Miwa K, Guibal C, Garibaldi L, Joly C, Zaccagnini ME (2025). https://zenodo.org/records/19821228.

[ref-49] Graham DJ, Hughes JM, Leder H, Rockmore DN (2012). Statistics, vision, and the analysis of artistic style. WIREs Computational Statistics.

[ref-50] Green RA (2014). The delphi technique in educational research. Sage Open.

[ref-51] Groskurth K, Bluemke M, Lechner CM (2023). Why we need to abandon fixed cutoffs for goodness-of-fit indices: an extensive simulation and possible solutions. Behavior Research Methods.

[ref-52] Hidi S, Renninger KA (2006). The four-phase model of interest development. Educational Psychologist.

[ref-53] Hitch GJ, Allen RJ, Baddeley AD (2024). The multicomponent model of working memory fifty years on. Quarterly Journal of Experimental Psychology.

[ref-54] Hůla M, Flegr J (2021). Habitat selection and human aesthetic responses to flowers. Evolutionary Human Sciences.

[ref-55] Hu L, Bentler PM (1998). Fit indices in covariance structure modeling: sensitivity to underparameterized model misspecification. Psychological Methods.

[ref-56] Jasanoff S (2005). Designs on nature: science and democracy in Europe and the United States.

[ref-57] Kaasinen A (2019). Plant species recognition skills in Finnish students and teachers. Education Sciences.

[ref-58] Kacprzyk J (2026). Philosophical foundations of plant awareness for sustainability. Trends in Plant Science.

[ref-59] Karjalainen E, Sarjala T, Raitio H (2010). Promoting human health through forests: overview and major challenges. Environmental Health and Preventive Medicine.

[ref-60] Kleespies MW, Braun T, Dierkes PW, Wenzel V (2021). Measuring connection to nature—a illustrated extension of the inclusion of nature in self scale. Sustainability.

[ref-61] Knight AT, Cowling RM, Rouget M, Balmford A, Lombard AT, Campbell BM (2008). Knowing but not doing: selecting priority conservation areas and the research–implementation gap. Conservation Biology.

[ref-62] Koivisto M, Kainulainen P, Revonsuo A (2009). The relationship between awareness and attention: evidence from ERP responses. Neuropsychologia.

[ref-63] Kollmuss A, Agyeman J (2002). Mind the gap: why do people act environmentally and what are the barriers to pro-environmental behavior?. Environmental Education Research.

[ref-64] Krapp A (2007). An educational–psychological conceptualisation of interest. International Journal for Educational and Vocational Guidance.

[ref-65] Krapp A, Prenzel M (2011). Research on interest in science: theories, methods, and findings. International Journal of Science Education.

[ref-66] Krüger D, Burmester A (2005). How students classify plants. Journal for Didactics of Natural Sciences.

[ref-67] Kuo FE (2001). Coping with poverty: impacts of environment and attention in the inner city. Environment and Behavior.

[ref-68] Lambert LS, Newman DA (2023). Construct development and validation in three practical steps: recommendations for reviewers, editors, and authors. Organizational Research Methods.

[ref-69] Lamme VAF (2003). Why visual attention and awareness are different. Trends in Cognitive Sciences.

[ref-70] Lamme VAF (2005). The difference between visual attention and awareness: a cognitive neuroscience perspective. Neurobiology of attention.

[ref-71] Lampert P, Müllner B, Pany P, Scheuch M, Kiehn M (2020). Students’ conceptions of plant reproduction processes. Journal of Biological Education.

[ref-72] Laumann K, Gärling T, Stormark KM (2003). Selective attention and heart rate responses to natural and urban environments. Journal of Environmental Psychology, Restorative Environments.

[ref-73] Ledgerwood A, Eastwick PW, Smith LK (2018). Toward an integrative framework for studying human evaluation: attitudes toward objects and attributes. Personality and Social Psychology Review.

[ref-74] Linacre JM (2023). https://www.winsteps.com/winsteps.htm.

[ref-75] Link-Pérez MA, Dollo VH, Weber KM, Schussler EE (2010). What’s in a name: differential labelling of plant and animal photographs in two nationally syndicated elementary science textbook series. International Journal of Science Education.

[ref-76] Locke EA (2003). Good definitions: the epistemological foundation of scientific progress. Organizational behavior: the state of the science.

[ref-77] Mach KJ, Lemos MC, Meadow AM, Wyborn C, Klenk N, Arnott JC, Ardoin NM, Fieseler C, Moss RH, Nichols L, Stults M, Vaughan C, Wong-Parodi G (2020). Actionable knowledge and the art of engagement. Current Opinion in Environmental Sustainability, Advancing the Science of Actionable Knowledge for Sustainability.

[ref-78] Maio GR, Haddock G, Kruglanski AW, Higgins ET (2007). Attitude change. Social psychology: handbook of basic principles.

[ref-79] Maller C, Townsend M, St Leger L, Henderson-Wilson C, Pryor A, Prosser L, Moore M (2009). Healthy parks, healthy people: the health benefits of contact with nature in a park context. The George Wright Forum.

[ref-80] Mammola S, Riccardi N, Prié V, Correia R, Cardoso P, Lopes-Lima M, Sousa R (2020). Towards a taxonomically unbiased European Union biodiversity strategy for 2030. Proceedings of the Royal Society B: Biological Sciences.

[ref-81] Marsh HW, Hau K-T, Wen Z (2004). In search of golden rules: comment on hypothesis-testing approaches to setting cutoff values for fit indexes and dangers in overgeneralizing Hu and Bentler’s (1999) findings. Structural Equation Modeling: A Multidisciplinary Journal.

[ref-82] Miller CC, Washburn NT, Glick WH (2013). Perspective—the myth of firm performance. Organization Science.

[ref-83] New J, Cosmides L, Tooby J (2007). Category-specific attention for animals reflects ancestral priorities, not expertise. Proceedings of the National Academy of Sciences of the United States of America.

[ref-84] Nisbet EK, Zelenski JM (2013). The NR-6: a new brief measure of nature relatedness. Frontiers in Psychology.

[ref-85] Nunnally J, Bernstein I (1994). Psychometric theory.

[ref-86] Nyberg E, Brkovic I, Sanders D (2021). Beauty, memories and symbolic meaning: Swedish student teachers views of their favourite plant and animal. Journal of Biological Education.

[ref-87] Nyberg E, Sanders D (2014). Drawing attention to the ‘green side of life’. Journal of Biological Education.

[ref-88] Pany P, Dünser B, Sanders DL, Pernausl LA, Kranner K, Wenzlik M, Fister D, Möller A, Lampert P (2026). Assessing attention towards plants: development and first steps to the validation of the Hidden Object Picture Instrument (HOPI). PLOS ONE.

[ref-89] Pany P, Meier FD, Dünser B, Yanagida T, Kiehn M, Möller A (2022). Measuring students’ plant awareness: a prerequisite for effective botany education. Journal of Biological Education.

[ref-90] Park B-J, Furuya K, Kasetani T, Takayama N, Kagawa T, Miyazaki Y (2011). Relationship between psychological responses and physical environments in forest settings. Landscape and Urban Planning.

[ref-91] Parsley KM (2020). Plant awareness disparity: a case for renaming plant blindness. Plants, People, Planet.

[ref-92] Parsley KM, Daigle BJ, Sabel JL (2022). Initial development and validation of the plant awareness disparity index. CBE—Life Sciences Education.

[ref-93] Patrick P, Tunnicliffe SD (2011). What plants and animals do early childhood and primary students’ name? Where do they see them?. Journal of Science Education and Technology.

[ref-94] Prinz W, Müsseler J, Rieger M (2017). General psychology.

[ref-95] Rensink RA, Huang W (2014). On the prospects for a science of visualization. Handbook of human centric visualization.

[ref-96] Revelle W (2020). https://cran.r-project.org/web/packages/psych/index.html.

[ref-97] Rosseel Y (2012). lavaan: an R package for structural equation modeling. Journal of Statistical Software.

[ref-98] Ryplova R, Pokorny J (2020). Saving water for the future via increasing plant literacy of pupils. European Journal of Sustainable Development.

[ref-99] Sanders DL (2007). Making public the private life of plants: the contribution of informal learning environments. International Journal of Science Education.

[ref-100] Sanders D, Nyberg E, Brkovic I (2024). Putting plants in the picture. Environmental Education Research.

[ref-101] Sandmann A, Krüger D, Parchmann I, Schecker H (2014). Thinking aloud: the analysis of thinking, learning, and problem-solving processes. Methods in science education research.

[ref-102] Schlegel J, Breuer G, Rupf R (2015). Local insects as flagship species to promote nature conservation? A survey among primary school children on their attitudes toward invertebrates. Anthrozoös.

[ref-103] Schreiner C, Sjøberg S (2004). Sowing the seeds of ROSE: background, rationale, questionnaire development and data collection for ROSE (The Relevance of Science Education) : a comparative study of students’ views of science and science education.

[ref-104] Schunko C, Brandner A (2022). Urban nature at the fingertips: investigating wild food foraging to enable nature interactions of urban dwellers. Ambio.

[ref-105] Schussler EE (2008). From flowers to fruits: how children’s books represent plant reproduction. International Journal of Science Education.

[ref-106] Schussler EE, Link-Pérez MA, Weber KM, Dollo VH (2010). Exploring plant and animal content in elementary science textbooks. Journal of Biological Education.

[ref-107] Schussler EE, Olzak LA (2008). It’s not easy being green: student recall of plant and animal images. Journal of Biological Education.

[ref-108] Soga M, Gaston KJ (2016). Extinction of experience: the loss of human–nature interactions. Frontiers in Ecology and the Environment.

[ref-109] Soga M, Gaston KJ (2018). Shifting baseline syndrome: causes, consequences, and implications. Frontiers in Ecology and the Environment.

[ref-110] Soga M, Gaston KJ (2020). The ecology of human–nature interactions. Proceedings of the Royal Society B: Biological Sciences.

[ref-111] Stagg BC, Dillon J (2022). Plant awareness is linked to plant relevance: a review of educational and ethnobiological literature (1998–2020). Plants, People, Planet.

[ref-112] Stagg BC, Hetherington L, Dillon J (2024). Towards a model of plant awareness in education: a literature review and framework proposal. International Journal of Science Education.

[ref-113] Strgar J (2008). How are age and gender related to attitude toward plants and animals?. Acta Biologica Slovenica.

[ref-114] Takayama N, Korpela K, Lee J, Morikawa T, Tsunetsugu Y, Park B-J, Li Q, Tyrväinen L, Miyazaki Y, Kagawa T (2014). Emotional, restorative and vitalizing effects of forest and urban environments at four sites in Japan. International Journal of Environmental Research and Public Health.

[ref-115] Tam K-P (2013). Concepts and measures related to connection to nature: similarities and differences. Journal of Environmental Psychology.

[ref-116] Taylor AF, Kuo FE, Sullivan WC (2001). Coping with ADD: the surprising connection to green play settings. Environment and Behavior.

[ref-117] Tsunetsugu Y, Lee J, Park B-J, Tyrväinen L, Kagawa T, Miyazaki Y (2013). Physiological and psychological effects of viewing urban forest landscapes assessed by multiple measurements. Landscape and Urban Planning.

[ref-118] Tunnicliffe SD, Reiss MJ (2000). Building a model of the environment: how do children see plants?. Journal of Biological Education.

[ref-119] Uno GE (2009). Botanical literacy: what and how should students learn about plants?. American Journal of Botany.

[ref-120] Urry LA, Cain ML, Wasserman SA, Minorsky PV, Reece JB (2017). Campbell biology.

[ref-121] Völzke K (2012). Think-aloud exercises in competency-oriented diagnostic tasks for scientific knowledge acquisition.

[ref-122] Wandersee JH (1986). Plants or animals—which do junior high school students prefer to study?. Journal of Research in Science Teaching.

[ref-123] Wandersee JH, Schussler E (2001). Toward a theory of plant blindness. Plant Science Bulletin.

[ref-124] Wolfe JM, Horowitz TS (2017). Five factors that guide attention in visual search. Nature Human Behaviour.

[ref-125] Xie B, Brewer MB, Hayes BK, McDonald RI, Newell BR (2019). Predicting climate change risk perception and willingness to act. Journal of Environmental Psychology.

