# Supplementary material for: Rooting plant awareness: development of a tool to explore human-plant relationships
Source: PeerJ. 2026 Sep 8;14:e21667. doi: 10.7717/peerj.21667 (PMC13564396; doi:10.7717/peerj.21667)
Supplement: Supplementary file 1 [file peerj-14-21667-s003.pdf]

Variable	Variable label	Response code	Response Label	Variable Type	Input Type
SD01	Age	1	9	NOMINAL	MC
SD01	Age	2	10	NOMINAL	MC
SD01	Age	3	11	NOMINAL	MC
SD01	Age	4	12	NOMINAL	MC
SD01	Age	5	13	NOMINAL	MC
SD01	Age	6	14	NOMINAL	MC
SD01	Age	7	15	NOMINAL	MC
SD01	Age	8	16	NOMINAL	MC
SD01	Age	9	17	NOMINAL	MC
SD01	Age	10	18	NOMINAL	MC
SD01	Age	11	19	NOMINAL	MC
SD01	Age	12	20	NOMINAL	MC
SD01	Age	-	No response	NOMINAL	MC
SD02	Gender	1	female	NOMINAL	MC
SD02	Gender	2	male	NOMINAL	MC
SD02	Gender	3	diverse	NOMINAL	MC
SD02	Gender	4	I don't want to answer that question.	NOMINAL	MC
SD02	Gender	-9	No response	NOMINAL	MC
Apple Tree		>0	selected	DICHOTOMOUS	CK
Pear Tree		>0	selected	DICHOTOMOUS	CK
Nettle		>0	selected	DICHOTOMOUS	CK
Strawberries		>0	selected	DICHOTOMOUS	CK
Spruce		>0	selected	DICHOTOMOUS	CK
Cereal Field		>0	selected	DICHOTOMOUS	CK
Cherry		>0	selected	DICHOTOMOUS	CK
Maize Field		>0	selected	DICHOTOMOUS	CK
Fruit Tree		>0	selected	DICHOTOMOUS	CK
Sunflowers		>0	selected	DICHOTOMOUS	CK
AT01_01	Plants and mood: very uncomfortable (bad)/very comfortable (good)	1	very uncomfortable (bad)	ORDINAL	MC
AT01_01	Plants and mood: very uncomfortable (bad)/very comfortable (good)	5	very well (good)	ORDINAL	MC
AT01_01	Plants and mood: very uncomfortable (bad)/very comfortable (good)	-9	No response	ORDINAL	MC
AT05_01	Aesthetics: not at all beautiful/very beautiful	1	Not at all beautiful	ORDINAL	MC
AT05_01	Aesthetics: not at all beautiful/very beautiful	5	very beautiful	ORDINAL	MC
AT05_01	Aesthetics: not at all nice/very nice	-9	No response	ORDINAL	MC
AT06_01	Culture: none at all/very important	1	None at all	ORDINAL	MC
AT06_01	Culture: none at all/very important	5	very important	ORDINAL	MC
AT06_01	Culture: none at all/very important	-9	No response	ORDINAL	MC
AT03_01	Environment: much less/much more	1	much less	ORDINAL	MC
AT03_01	Environment: much less/much more	5	much more	ORDINAL	MC
AT03_01	Environment: much less/much more	-9	No response	ORDINAL	MC
AT04_01	Cityscape: much less/much more	1	much less	ORDINAL	MC
AT04_01	Cityscape: much less/much more	5	much more	ORDINAL	MC
AT04_01	Cityscape: much less/much more	-9	No response	ORDINAL	MC
E101	Bellis perennis	1	Daisy	NOMINAL	MC
E101	Bellis perennis	2	Daisy	NOMINAL	MC
E101	Bellis perennis	3	Dandelion	NOMINAL	MC
E101	Bellis perennis	4	Meadow clover	NOMINAL	MC
E101	Bellis perennis	-9	No response	NOMINAL	MC
E113	Taraxacum officinalis	1	Dandelion1_200x242.jpg	NOMINAL	MC
E113	Taraxacum officinalis	2	Sunflower4_200x286.jpg	NOMINAL	MC
E113	Taraxacum officinalis	3	13_Illustration_Lotus_corniculatus_noC	NOMINAL	MC
E113	Taraxacum officinalis	4	15_Trifolium_pratensae_biolib_noC_20	NOMINAL	MC
E113	Taraxacum officinalis	-9	No response	NOMINAL	MC
E103	Picea abies	1	Habitus3_Spruce_200x366.jpg	NOMINAL	MC
E103	Picea abies	2	Habitus3_Foehre_200x292.jpg	NOMINAL	MC
E103	Picea abies	3	Habitus_Saeulenpappel_200x461.jpg	NOMINAL	MC
E103	Picea abies	4	Habitus2_Winterlinde_200x300.jpg	NOMINAL	MC
E103	Picea abies	-9	No response	NOMINAL	MC
E104	Subscale spices: alternative option (negative) or number of selected options			METRIC	TXT
E104_01	Subscale spices: Thyme	1	not selected	DICHOTOMOUS	CK
E104_01	Subscale spices: Thyme	2	selected	DICHOTOMOUS	CK
E104_02	Subscale spices: Marjoram	1	Not selected	DICHOTOMOUS	CK
E104_02	Subscale spices: Marjoram	2	selected	DICHOTOMOUS	CK
E104_03	Subscale spices: Oregano	1	Not selected	DICHOTOMOUS	CK
E104_03	Subscale spices: Oregano	2	selected	DICHOTOMOUS	CK
E104_04	Subscale spices: Basil	1	Not selected	DICHOTOMOUS	CK
E104_04	Subscale spices: Basil	2	selected	DICHOTOMOUS	CK
E104_05	Subscale spices: Pepper	1	Not selected	DICHOTOMOUS	CK
E104_05	Subscale spices: Pepper	2	selected	DICHOTOMOUS	CK
E117_01_correct	Ethno (name Tilia): [01]			TEXT	TXT
E118_01_correct	Ethno (name Rubus): [01]			TEXT	TXT
E203	Collection Blossom Linden	1	Never before	NOMINAL	MC
E203	Blossom Collection Lime Tree	2	1 - 5 times per year	NOMINAL	MC
E203	Blossom Collection Lime Tree	4	More than 5 times per year	NOMINAL	MC
E203	Blossom Collection Linden	5	(Almost) whenever this plant blooms	NOMINAL	MC
E203	Collection Blossom Linden	-9	No response	NOMINAL	MC
E206	Collection Flower Dandelion	1	Never	NOMINAL	MC
E206	Collection Blossom Dandelion	2	1 - 5 times per year	NOMINAL	MC
E206	Collection Flower Dandelion	4	More than 5 times per year	NOMINAL	MC
E206	Collection Flower Dandelion	5	(Almost) whenever this plant blooms	NOMINAL	MC
E206	Collection Flower Dandelion	-9	No response	NOMINAL	MC
E205	Collection Blossom Daisy	1	Never	NOMINAL	MC
E205	Collection Blossom Daisy	2	1 - 5 times per year	NOMINAL	MC
E205	Collection Blossom Daisy	4	More than 5 times per year	NOMINAL	MC
E205	Collection Blossom Daisy	5	(Almost) whenever this plant blooms	NOMINAL	MC
E205	Collection Flower Daisy	-9	No response	NOMINAL	MC
E204	Fruit collection Blackberry	1	Never	NOMINAL	MC
E204	Fruit Collection Blackberry	2	1 - 5 times per year	NOMINAL	MC
E204	Collection Fruit Blackberry	4	more than 5 times per year	NOMINAL	MC
E204	Collection Fruit Blackberry	5	(Almost) Whenever this plant bears fruit	NOMINAL	MC
E204	Collection Fruit Blackberry	-9	No response	NOMINAL	MC
K101_01_1	Living beings_Characteristics: Can move in different ways./Bacteria	1	not selected	DICHOTOMOUS	CK
K101_01_1	Living beings_Characteristics: Can move in different ways./Bacteria	2	selected	DICHOTOMOUS	CK
K101_01_2	Living beings_Characteristics: Can move in different ways./Plants	1	Not selected	DICHOTOMOUS	CK
K101_01_2	Living beings_Characteristics: Can move in different ways./Plants	2	selected	DICHOTOMOUS	CK
K101_01_3	Living beings_Characteristics: Can move in different ways./Animals	1	not selected	DICHOTOMOUS	CK
K101_01_3	Living beings_Characteristics: Can move in different ways./Animals	2	selected	DICHOTOMOUS	CK
K101_01_4	Living beings_Characteristics: Can move in different ways./Fungi	1	Not selected	DICHOTOMOUS	CK
K101_01_4	Living beings_Characteristics: Can move in different ways./Fungi	2	selected	DICHOTOMOUS	CK

K101_02_1	Living beings_Characteristics: Consist of one or more cells./Bacteria	1	Not selected	DICHOTOMOUS	CK
K101_02_1	Living beings_Characteristics: Consist of one or more cells./Bacteria	2	selected	DICHOTOMOUS	CK
K101_02_2	Living beings_Characteristics: Consist of one or more cells./Plants	1	Not selected	DICHOTOMOUS	CK
K101_02_2	Living beings_Characteristics: Consist of one or more cells./Plants	2	selected	DICHOTOMOUS	CK
K101_02_3	Living beings_Characteristics: Consist of one or more cells./Animals	1	not selected	DICHOTOMOUS	CK
K101_02_3	Living beings_Characteristics: Consist of one or more cells./Animals	2	selected	DICHOTOMOUS	CK
K101_02_4	Living beings_Characteristics: Consist of one or more cells./Fungi	1	not selected	DICHOTOMOUS	CK
K101_02_4	Living beings_Characteristics: Consist of one or more cells./Fungi	2	selected	DICHOTOMOUS	CK
K101_03_1	Living beings_Characteristics: Absorb nutrients, convert them, and excrete unwanted substances./Bacteria	1	not selected	DICHOTOMOUS	CK
K101_03_1	Living beings_Characteristics: Absorb nutrients, convert them, and release unused substances./Bacteria	2	selected	DICHOTOMOUS	CK
K101_03_2	Living beings_Characteristics: Absorb nutrients, convert them, and release unused substances./Plants	1	not selected	DICHOTOMOUS	CK
K101_03_2	Living beings_Characteristics: Absorb nutrients, convert them, and release unused substances./Plants	2	selected	DICHOTOMOUS	CK
K101_03_3	Living beings_Characteristics: Absorb nutrients, convert them, and excrete unwanted substances./Animals	1	not selected	DICHOTOMOUS	CK
K101_03_3	Living beings_Characteristics: Absorb nutrients, convert them, and excrete waste products that are not needed. substances./Animals	2	selected	DICHOTOMOUS	CK
K101_03_4	Living beings_Characteristics: Absorb nutrients, convert them, and release unused substances./Fungi	1	not selected	DICHOTOMOUS	CK
K101_03_4	Living beings_Characteristics: Absorb nutrients, convert them, and release unused substances./Fungi	2	selected	DICHOTOMOUS	CK
K101_04_1	Living beings_Characteristics: React to their environment (are irritable)./Bacteria	1	not selected	DICHOTOMOUS	CK
K101_04_1	Living beings_Characteristics: React to their environment (are irritable)./Bacteria	2	selected	DICHOTOMOUS	CK
K101_04_2	Living beings_Characteristics: Respond to their environment (are irritable)./Plants	1	not selected	DICHOTOMOUS	CK
K101_04_2	Living beings_Characteristics: React to their environment (are irritable)./Plants	2	selected	DICHOTOMOUS	CK
K101_04_3	Living beings_Characteristics: React to their environment (are irritable)./Animals	1	not selected	DICHOTOMOUS	CK
K101_04_3	Living beings_Characteristics: React to their environment (are irritable)./Animals	2	selected	DICHOTOMOUS	CK
K101_04_4	Living beings_Characteristics: React to their environment (are irritable)./Fungi	1	not selected	DICHOTOMOUS	CK
K101_04_4	Living beings_Characteristics: React to their environment (are irritable)./Fungi	2	selected	DICHOTOMOUS	CK
K101_05_1	Living beings_Characteristics: Reproduce (multiply)./Bacteria	1	Not selected	DICHOTOMOUS	CK
K101_05_1	Living beings_Characteristics: Reproduce (multiply)./Bacteria	2	selected	DICHOTOMOUS	CK
K101_05_2	Living beings_Characteristics: Reproduce (multiply)./Plants	1	not selected	DICHOTOMOUS	CK
K101_05_2	Living beings_Characteristics: Reproduce (multiply)./Plants	2	selected	DICHOTOMOUS	CK
K101_05_3	Living beings_Characteristics: Reproduce (multiply)./Animals	1	not selected	DICHOTOMOUS	CK
K101_05_3	Living beings_Characteristics: Reproduce (multiply)./Animals	2	selected	DICHOTOMOUS	CK
K101_05_4	Living beings_Characteristics: Reproduce (multiply)./Fungi	1	Not selected	DICHOTOMOUS	CK
K101_05_4	Living beings_Characteristics: Reproduce (multiply)./Fungi	2	selected	DICHOTOMOUS	CK
K101_06_1	Living beings_Characteristics: Pass on characteristics to their offspring./Bacteria	1	not selected	DICHOTOMOUS	CK
K101_06_1	Living beings_Characteristics: Pass on characteristics to their offspring./Bacteria	2	selected	DICHOTOMOUS	CK
K101_06_2	Living beings_Characteristics: Pass on characteristics to their offspring./Plants	1	Not selected	DICHOTOMOUS	CK
K101_06_2	Living beings_Characteristics: Pass on characteristics to their offspring./Plants	2	selected	DICHOTOMOUS	CK
K101_06_3	Living beings_Characteristics: Pass on characteristics to their offspring./Animals	1	not selected	DICHOTOMOUS	CK
K101_06_3	Living beings_Characteristics: Pass on characteristics to their offspring./Animals	2	selected	DICHOTOMOUS	CK
K101_06_4	Living beings_Characteristics: Pass on characteristics to their offspring./Fungi	1	not selected	DICHOTOMOUS	CK
K101_06_4	Living beings_Characteristics: Pass on characteristics to their offspring./Fungi	2	selected	DICHOTOMOUS	CK
K101_07_1	Living beings_Characteristics: Capable of growth./Bacteria	1	Not selected	DICHOTOMOUS	CK
K101_07_1	Living beings_Characteristics: Capable of growth./Bacteria	2	selected	DICHOTOMOUS	CK
K101_07_2	Living beings_Characteristics: Capable of growth./Plants	1	Not selected	DICHOTOMOUS	CK
K101_07_2	Living beings_Characteristics: Capable of growth./Plants	2	selected	DICHOTOMOUS	CK
K101_07_3	Living beings_Characteristics: Capable of growth./Animals	1	Not selected	DICHOTOMOUS	CK
K101_07_3	Living beings_Characteristics: Capable of growth./Animals	2	selected	DICHOTOMOUS	CK
K101_07_4	Living beings_Characteristics: Capable of growth./Fungi	1	Not selected	DICHOTOMOUS	CK
K101_07_4	Living beings_Characteristics: Capable of growth./Fungi	2	selected	DICHOTOMOUS	CK
K101_08_1	Living beings_Characteristics: Can become ill./Bacteria	1	Not selected	DICHOTOMOUS	CK
K101_08_1	Living beings_Characteristics: Can become ill./Bacteria	2	selected	DICHOTOMOUS	CK
K101_08_2	Living beings_Characteristics: Can become ill./Plants	1	Not selected	DICHOTOMOUS	CK
K101_08_2	Living beings_Characteristics: Can become ill./Plants	2	selected	DICHOTOMOUS	CK
K101_08_3	Living beings_Characteristics: Can become ill./Animals	1	Not selected	DICHOTOMOUS	CK
K101_08_3	Living beings_Characteristics: Can become ill./Animals	2	selected	DICHOTOMOUS	CK
K101_08_4	Living beings_Characteristics: Can become ill./Fungi	1	Not selected	DICHOTOMOUS	CK
K101_08_4	Living beings_Characteristics: Can become ill./Fungi	2	selected	DICHOTOMOUS	CK
K209_01	Carbon cycle: Forests	1	Not selected	DICHOTOMOUS	CK
K209_01	Carbon cycle: Forests	2	selected	DICHOTOMOUS	CK
K209_02	Carbon cycle: Algae in the sea	1	Not selected	DICHOTOMOUS	CK
K209_02	Carbon cycle: Algae in the sea	2	selected	DICHOTOMOUS	CK
K209_03	Carbon cycle: Air traffic	1	Not selected	DICHOTOMOUS	CK
K209_03	Carbon cycle: Air traffic	2	selected	DICHOTOMOUS	CK
K209_04	Carbon cycle: Oil power plants	1	Not selected	DICHOTOMOUS	CK
K209_04	Carbon cycle: Oil power plants	2	selected	DICHOTOMOUS	CK
K209_05	Carbon cycle: Pasture farming (e.g., cows)	1	Not selected	DICHOTOMOUS	CK
K209_05	Carbon cycle: Pasture farming (e.g., cows)	2	selected	DICHOTOMOUS	CK
K209_06	Carbon cycle: Car traffic	1	Not selected	DICHOTOMOUS	CK
K209_06	Carbon cycle: Car traffic	2	selected	DICHOTOMOUS	CK
K205_01	Agricultural Blindness: Soil Quality	1	not selected	DICHOTOMOUS	CK
K205_01	Agricultural Blindness: The Quality of Soils	2	selected	DICHOTOMOUS	CK
K205_02	Agricultural Blindness: Elevated Temperatures	1	Not selected	DICHOTOMOUS	CK
K205_02	Agricultural Blindness: Elevated Temperatures	2	selected	DICHOTOMOUS	CK
K205_03	Agricultural Blindness: The Insect Dieback	1	not selected	DICHOTOMOUS	CK
K205_03	Agricultural Blindness: The Insect Dieback	2	selected	DICHOTOMOUS	CK
K205_04	Agricultural Blindness: Extreme Weather Events	1	Not selected	DICHOTOMOUS	CK
K205_04	Agricultural Blindness: Extreme Weather Events	2	selected	DICHOTOMOUS	CK
K205_05	Agricultural Blindness: Destruction of Habitats	1	not selected	DICHOTOMOUS	CK
K205_05	Agricultural Blindness: Destruction of Habitats	2	selected	DICHOTOMOUS	CK
K206_01	Sustainable clothing: Hemp pants	1	Not selected	DICHOTOMOUS	CK
K206_01	Sustainability Clothing: Hemp pants	2	Selected	DICHOTOMOUS	CK
K206_02	Sustainable clothing: Bamboo socks	1	Not selected	DICHOTOMOUS	CK
K206_02	Sustainability Clothing: Bamboo socks	2	Selected	DICHOTOMOUS	CK
K206_03	Sustainability Clothing: Cotton T-shirt	1	Not selected	DICHOTOMOUS	CK
K206_03	Sustainability Clothing: Cotton T-shirt	2	Selected	DICHOTOMOUS	CK
K206_04	Sustainability Clothing: Linen shirt	1	Not selected	DICHOTOMOUS	CK
K206_04	Sustainability Clothing: Linen shirt	2	Selected	DICHOTOMOUS	CK
K206_05	Sustainability Clothing: Polyester running shirt	1	Not selected	DICHOTOMOUS	CK
K206_05	Sustainability Clothing: Polyester running shirt	2	Selected	DICHOTOMOUS	CK
K207_01	Sustainability Energy production: Wood	1	Not selected	DICHOTOMOUS	CK
K207_01	Sustainability Energy production: Wood	2	selected	DICHOTOMOUS	CK
K207_02	Sustainability Energy production: Biofuel	1	Not selected	DICHOTOMOUS	CK
K207_02	Sustainability Energy production: Biofuel	2	selected	DICHOTOMOUS	CK

NR01_01	NR: I enjoy being outdoors in nature	1	Not applicable	ORDINAL	MC
NR01_01	NR: I enjoy being outdoors in nature	5	Completely true	ORDINAL	MC
NR01_01	NR: I enjoy being outdoors in nature	-9	No response	ORDINAL	MC
NR01_02	NR: My favorite places are outdoors in nature	1	Not applicable	ORDINAL	MC
NR01_02	NR: My favorite places are outdoors in nature	5	Totally agree	ORDINAL	MC
NR01_02	NR: My favorite places are outdoors in nature	-9	No response	ORDINAL	MC
NR01_03	NR: When I can, I spend my time outdoors.	1	Not applicable	ORDINAL	MC
NR01_03	NR: When I can, I spend my time outdoors.	5	Completely true	ORDINAL	MC
NR01_03	NR: When I can, I spend my time outdoors.	-9	No response	ORDINAL	MC
NR01_04	NR: I feel very connected to all living beings and to the earth.	1	Not applicable	ORDINAL	MC
NR01_04	NR: I feel very connected to all living beings and the earth.	5	Completely true	ORDINAL	MC
NR01_04	NR: I feel very connected to all living beings and the earth.	-9	No answer	ORDINAL	MC
NR01_06	NR: My relationship with nature is an important part of my personality	1	Not applicable	ORDINAL	MC
NR01_06	NR: My relationship with nature is an important part of my personality	5	Completely true	ORDINAL	MC
NR01_06	NR: My relationship with nature is an important part of my personality	-9	No response	ORDINAL	MC
NR01_07	NR: I consider the impact my behavior has on the earth.	1	Not applicable	ORDINAL	MC
NR01_07	NR: I consider the impact my behavior has on the earth.	5	Completely true	ORDINAL	MC
NR01_07	NR: I consider the impact my behavior has on the earth.	-9	No answer	ORDINAL	MC
NR02	Nature and self scale	1	A	NOMINAL	MC
NR02	Nature and self scale	2	B	NOMINAL	MC
NR02	Nature and self scale	3	C	NOMINAL	MC
NR02	Nature and self scale	4	D	NOMINAL	MC
NR02	Nature and self scale	5	E	NOMINAL	MC
NR02	Nature and self scale	6	F	NOMINAL	MC
NR02	Nature and self scale	7	G	NOMINAL	MC
NR02	Nature and self scale	-9	No response	NOMINAL	MC
PI01_01	Rose Interest: How plants grow and reproduce.	1	Not interested at all	ORDINAL	MC
PI01_01	Rose Interest: How plants grow and reproduce.	2	Not really interested	ORDINAL	MC
PI01_01	Rose Interest: How plants grow and reproduce.	3	I'm more interested in	ORDINAL	MC
PI01_01	Rose Interest: How plants grow and reproduce.	4	Very interested	ORDINAL	MC
PI01_01	Rose Interest: How plants grow and reproduce.	-9	No answer	ORDINAL	MC
PI01_02	Rose Interest: How humans, animals, plants, and their environment depend on each other.	1	Not interested at all	ORDINAL	MC
PI01_02	Rose Interest: How humans, animals, plants, and their environment depend on each other.	2	I'm not really interested	ORDINAL	MC
PI01_02	Rose Interest: How humans, animals, plants, and their environment depend on each other.	3	I'm more interested in	ORDINAL	MC
PI01_02	Rose Interest: How humans, animals, plants, and their environment depend on each other.	4	Very interested	ORDINAL	MC
PI01_02	Rose Interest: How humans, animals, plants, and their environment depend on each other.	-9	No response	ORDINAL	MC
PI01_03	Rose Interest: Poisonous plants in my environment.	1	Not interested at all	ORDINAL	MC
PI01_03	Rose Interest: Poisonous plants in my environment.	2	Not really interested	ORDINAL	MC
PI01_03	Rose Interest: Poisonous plants in my environment.	3	I'm more interested in	ORDINAL	MC
PI01_03	Rose Interest: Poisonous plants in my area.	4	Very interested	ORDINAL	MC
PI01_03	Rose Interest: Poisonous plants in my area.	-9	No response	ORDINAL	MC
PI01_04	Rose Interest: Patterns and shapes of leaves and flowers.	1	Not interested at all	ORDINAL	MC
PI01_04	Rose Interest: Patterns and shapes of leaves and flowers.	2	Not really interested	ORDINAL	MC
PI01_04	Rose Interest: Patterns and shapes of leaves and flowers.	3	I'm more interested in	ORDINAL	MC
PI01_04	Rose Interest: Patterns and shapes of leaves and flowers.	4	Very interested	ORDINAL	MC
PI01_04	Rose Interest: Patterns and shapes of leaves and flowers.	-9	No response	ORDINAL	MC
PI01_05	Rose Interest: The greenhouse effect and how humans influence it.	1	Not interested at all	ORDINAL	MC
PI01_05	Rose Interest: The greenhouse effect and how humans influence it.	2	Not really interested	ORDINAL	MC
PI01_05	Rose Interest: The greenhouse effect and how humans influence it.	3	I'm more interested in	ORDINAL	MC
PI01_05	Rose Interest: The greenhouse effect and how humans influence it.	4	Very interested	ORDINAL	MC
PI01_05	Rose Interest: The greenhouse effect and how humans influence it.	-9	No response	ORDINAL	MC
PI01_06	Rose Interest: How to protect and save plants threatened with extinction.	1	Not interested	ORDINAL	MC
PI01_06	Rose Interest: How to protect and save endangered plants.	2	Not really interested	ORDINAL	MC
PI01_06	Rose Interest: How to protect and save endangered plants.	3	I'm more interested in	ORDINAL	MC
PI01_06	Rose Interest: How to protect and save endangered plants.	4	Very interested	ORDINAL	MC
PI01_06	Rose Interest: How to protect and save plants threatened with extinction.	-9	Unanswered	ORDINAL	MC
PI01_07	Rose Interest: How to improve crop yields in gardens and agriculture<br>.	1	Not interested	ORDINAL	MC
PI01_07	Rose Interest: How to improve the harvest of plants in gardens and agriculture<br>.	2	Not really interested	ORDINAL	MC
PI01_07	Rose Interest: How to improve crop yields in gardens and agriculture<br>.	3	I'm more interested in	ORDINAL	MC
PI01_07	Rose Interest: How to improve the harvest of plants in gardens and agriculture<br>.	4	Very interested	ORDINAL	MC
PI01_07	Rose Interest: How to improve the harvest of plants in gardens and agriculture<br>.	-9	No response	ORDINAL	MC
PI01_08	Rose Interest: How plants are used in medicine.	1	Not interested at all	ORDINAL	MC
PI01_08	Rose Interest: How plants are used in medicine.	2	Not really interested	ORDINAL	MC
PI01_08	Rose Interest: How plants are used in medicine.	3	I'm more interested in	ORDINAL	MC
PI01_08	Rose Interest: How plants are used in medicine.	4	Very interested	ORDINAL	MC
PI01_08	Rose Interest: How plants are used in medicine.	-9	No response	ORDINAL	MC
PI01_09	Rose Interest: Organic cultivation of plants without pesticides.	1	Not interested at all	ORDINAL	MC
PI01_09	Rose Interest: Organic cultivation of plants without pesticides.	2	Not really interested	ORDINAL	MC
PI01_09	Rose Interest: Organic cultivation of plants without pesticides.	3	I'm more interested in	ORDINAL	MC
PI01_09	Rose Interest: Organic cultivation of plants without pesticides.	4	Very interested	ORDINAL	MC
PI01_09	Rose Interest: Organic cultivation of plants without pesticides.	-9	No response	ORDINAL	MC
PI01_10	Rose Interest: Plants in my environment.	1	Not interested at all	ORDINAL	MC
PI01_10	Rose Interest: Plants in my area.	2	Not really interested	ORDINAL	MC
PI01_10	Rose Interest: Plants in my environment.	3	I'm more interested in	ORDINAL	MC
PI01_10	Rose Interest: Plants in my environment.	4	Very interested	ORDINAL	MC
PI01_10	Rose Interest: Plants in my environment.	-9	No response	ORDINAL	MC
PI02_01	Out of school: Do you collect fruits, mushrooms, berries, or plants?	1	Never	ORDINAL	MC
PI02_01	Out of school: Do you collect fruits, mushrooms, berries, or plants?	2	Rarely	ORDINAL	MC
PI02_01	Out of school: Do you collect fruits, mushrooms, berries, or plants?	3	Often	ORDINAL	MC
PI02_01	Out of school: Do you collect fruits, mushrooms, berries, or plants?	4	Very often	ORDINAL	MC
PI02_01	Out of school: Do you collect fruits, mushrooms, berries, or plants?	-9	No answer	ORDINAL	MC
PI02_02	Out of school: Do you plant seeds?	1	Never	ORDINAL	MC
PI02_02	Out of school: Do you plant seeds?	2	Rare	ORDINAL	MC
PI02_02	Out of school: Do you plant seeds?	3	often	ORDINAL	MC
PI02_02	Out of school: Do you plant seeds?	4	Very often	ORDINAL	MC
PI02_02	Out of school: Do you plant seeds?	-9	No answer	ORDINAL	MC
PI02_03	Out of school: Do you take plants as medicine?	1	Never	ORDINAL	MC
PI02_03	Out of school: Do you use plants as medicine?	2	Rare	ORDINAL	MC
PI02_03	Out of school: Do you use plants as medicine?	3	often	ORDINAL	MC
PI02_03	Out of school: Do you use plants as medicine?	4	Very often	ORDINAL	MC
PI02_03	Out of school: Do you use plants as medicine?	-9	No answer	ORDINAL	MC
PI02_04	Out of school: Do you water plants?	1	Never	ORDINAL	MC
PI02_04	Out of school: Do you water plants?	2	Rare	ORDINAL	MC
PI02_04	Out of school: Do you water plants?	3	often	ORDINAL	MC
PI02_04	Out of school: Do you water plants?	4	Very often	ORDINAL	MC

PI02_04	Out of school: Do you water plants?	-9	No answer	ORDINAL	MC
