# Supplementary material for: Rooting plant awareness: development of a tool to explore human-plant relationships
Source: PeerJ. 2026 Sep 8;14:e21667. doi: 10.7717/peerj.21667 (PMC13564396; doi:10.7717/peerj.21667)
Supplement: Supplementary file 4 [file peerj-14-21667-s006.pdf]

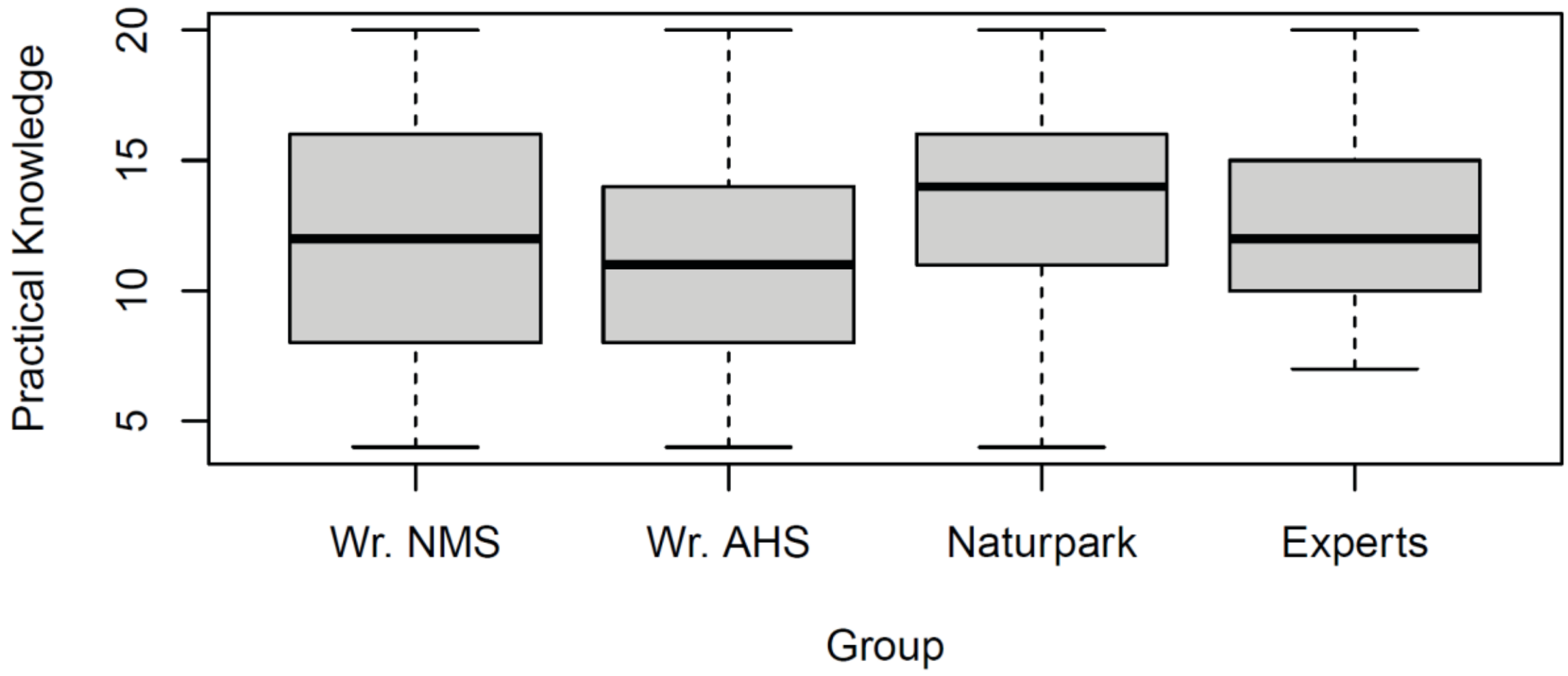

Figure 3S: Boxplots presenting the differences between the four subsamples *Mittelschule (Wr. NMS), Gymnasium (Wr. AHS), Naturparkschule,* and experts, regarding the *practical knowledge* subscale of the knowledge scale. Since this scale also tests frequency of collecting plants, students from *Naturparkschulen* who live close to a nature park collect plants more often than experts who are rather located in an urban area
