# Supplementary material for: Rooting plant awareness: development of a tool to explore human-plant relationships
Source: PeerJ. 2026 Sep 8;14:e21667. doi: 10.7717/peerj.21667 (PMC13564396; doi:10.7717/peerj.21667)
Supplement: Supplementary file 6 [file peerj-14-21667-s008.pdf]

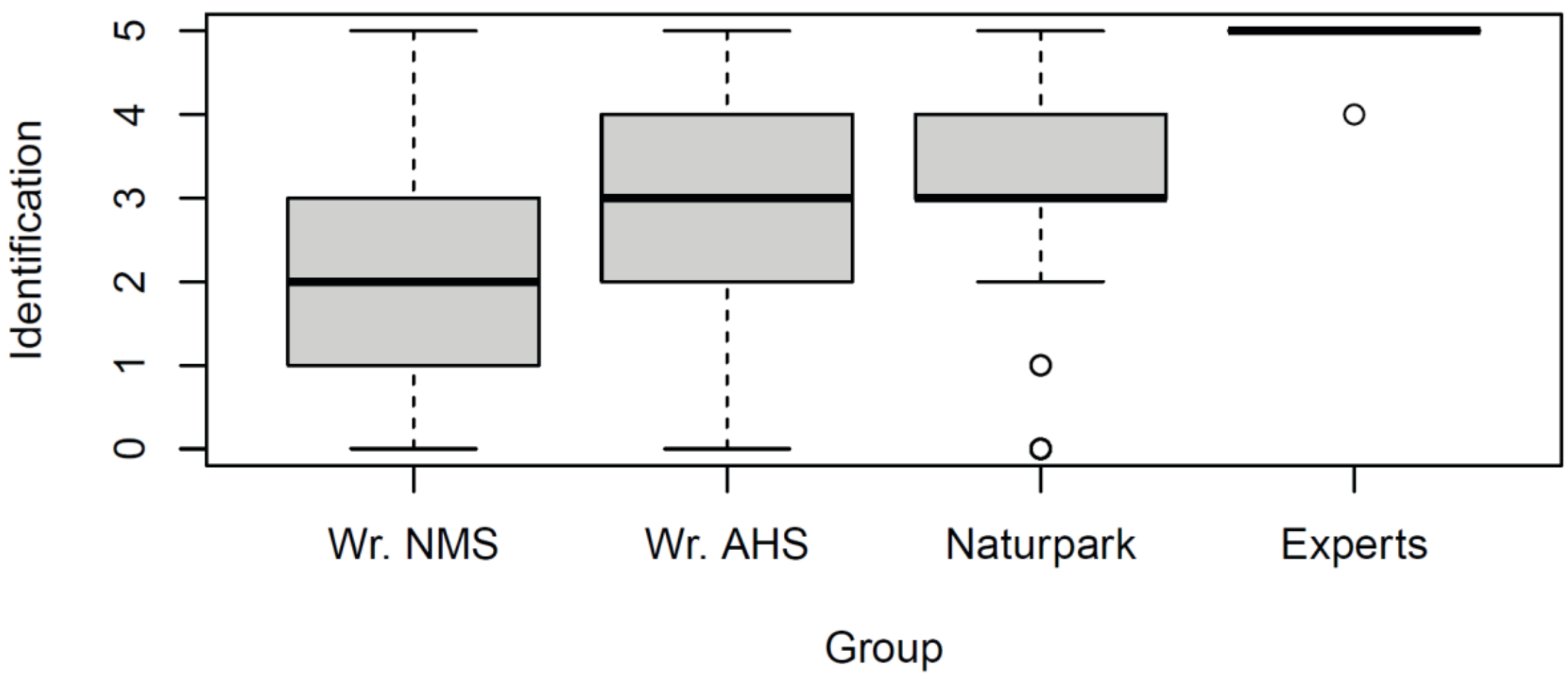

Figure 5S: Boxplots presenting the differences between the four subsamples *Mittelschule (Wr. NMS), Gymnasium (Wr. AHS), Naturparkschule,* and experts, regarding the *plant identification* subscale of the knowledge scale
