# Supplementary material for: Rooting plant awareness: development of a tool to explore human-plant relationships
Source: PeerJ. 2026 Sep 8;14:e21667. doi: 10.7717/peerj.21667 (PMC13564396; doi:10.7717/peerj.21667)
Supplement: Supplementary file 9 [file peerj-14-21667-s011.pdf]

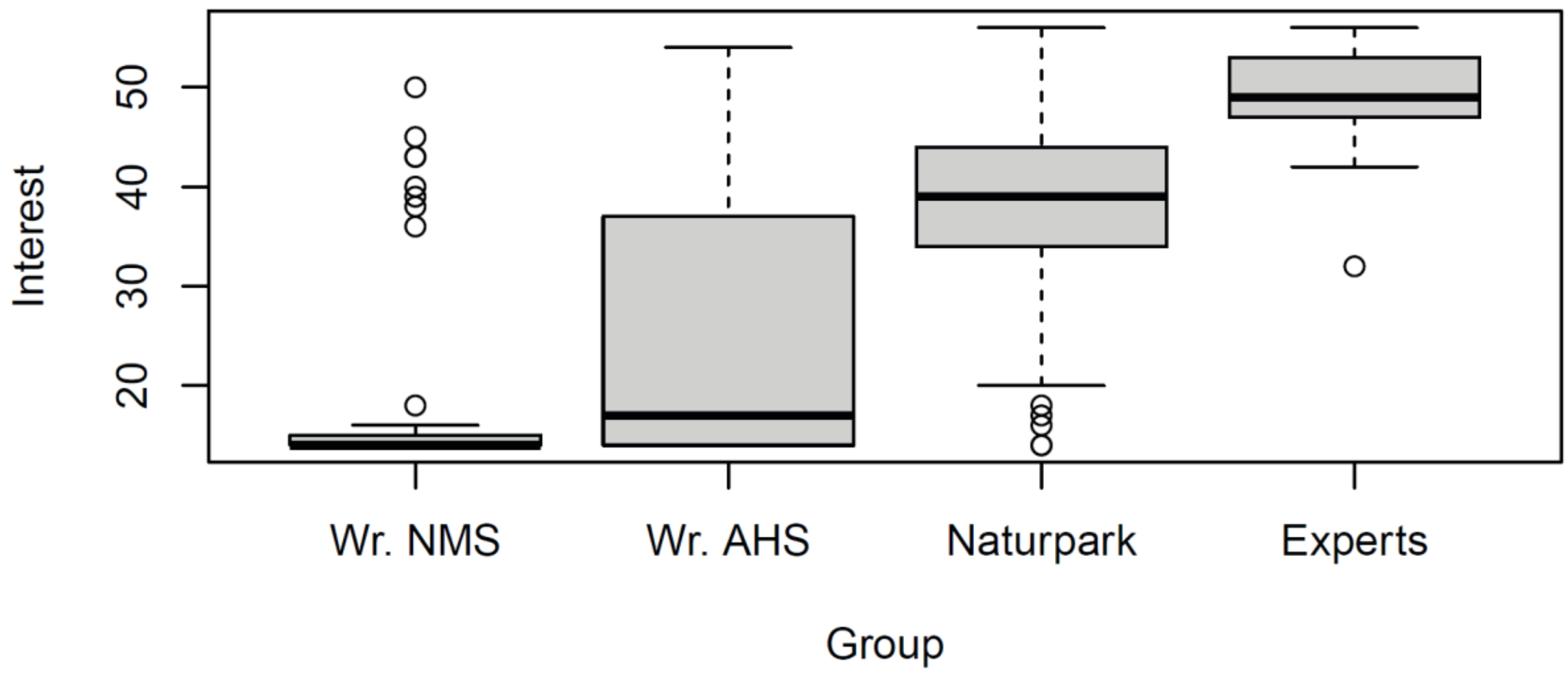

Figure 8S: Boxplots showing differences in interest for plants between the four subsamples *Mittelschule (Wr. NMS), Gymnasium (Wr. AHS), Naturparkschule,* and experts
