# Supplementary material for: Rooting plant awareness: development of a tool to explore human-plant relationships
Source: PeerJ. 2026 Sep 8;14:e21667. doi: 10.7717/peerj.21667 (PMC13564396; doi:10.7717/peerj.21667)
Supplement: Supplementary file 10 [file peerj-14-21667-s012.pdf]

Table 1S: The item universe, showing the origin and genesis of the subscales, along with their relationship to the SDGs and their distribution within the dimensions

Subscale	Description	Type	Items based on	SDG relation
		Attention		
-	Recall task Hidden Object Picture	Free-listing	(Schussler & Olzak, 2008)	
		***Knowledge** local*		
Plant identification	Common plant species identification (naming)	Single choice	(Buck et al., 2019; Kaasinen, 2019; Pany et al., 2022)	15
	Common plant species identification (choosing picture)	Single choice	(Buck et al., 2019; Kaasinen, 2019; Pany et al., 2022)	15
	Common plant species recognition (picture)	Single choice	(Schunko & Brandner, 2022)	15
Spice plants	Identifying common spice plants (by name)	Multiple choice	(Pany et al., 2022)	(3), 15
Practical knowledge	Harvesting frequency	Likert scale	(Schunko & Brandner, 2022)	2, 3, 15
		***Knowledge** global*		
Living organism	Categorization of plants as living organism	Single Choice	(Pany et al., 2022; Urry et al., 2017)	15
Carbon cycle	Carbon Cycle	Multiple Choice	(Asshoff et al., 2019; Düsing et al., 2019; Hartley et al., 2011)	7,12,13
Agriculture	Food & Reproduction	Multiple choice	(Lampert et al., 2020)	2,12,13
Sustainability	Sustainable energy & material	Multiple choice	(Dünser, 2022)	7, 12
		Attitude		
-	Plants in school		(Berto, 2005; Dünser et al., 2024; Laumann et al., 2003; Taylor et al., 2001)	
	Comfortable around plants		(Dünser et al., 2024; Gladwell et al., 2012; Karjalainen et al., 2010; Park et al., 2010)	
	Plants in the city		(Dünser et al., 2024; Kuo, 2001; Maller et al., 2009; Takayama et al., 2014)	
	Aesthetic qualities		(Dünser et al., 2024; Hůla & Flegr, 2021; Nyberg, 2017)	
	Cultural services		(Berkes, 2017; Dünser et al., 2024)	

References for the item universe

Asshoff, R., Düsing, K., Winkelmann, T., & Hammann, M. (2019). Considering the levels of biological organisation when teaching carbon flows in a terrestrial ecosystem. *Journal of Biological Education*, 1–14. https://doi.org/10.1080/00219266.2019.1575263

Berkes, F. (2017). *Sacred ecology*. Routledge. https://api.taylorfrancis.com/content/books/mono/download?identifierName=doi&identifierValue=10.4324/9781315114644&type=googlepdf

Berto, R. (2005). Exposure to restorative environments helps restore attentional capacity. *Journal of Environmental Psychology*, *25*(3), 249–259.

Buck, T., Bruchmann, I., Zumstein, P., & Drees, C. (2019). Just a small bunch of flowers: The botanical knowledge of students and the positive effects of courses in plant identification at German universities. *PeerJ*, *7*, e6581. https://doi.org/10.7717/peerj.6581

Dünser, B. (2022). *Pilotierung eines Fragebogens zur Quantifizierung von Plant Blindness* [Master thesis, University of Vienna]. https://utheses.univie.ac.at/detail/64189/#

Dünser, B., Möller, A., Fondriest, V., Boeckle, M., Lampert, P., & Pany, P. (2024). Attitudes towards plants – exploring the role of plants' ecosystem services. *Journal of Biological Education*, *59*(1), 124–138. https://doi.org/10.1080/00219266.2024.2308293

Düsing, K., Asshoff, R., & Hammann, M. (2019). Students' conceptions of the carbon cycle: Identifying and interrelating components of the carbon cycle and tracing carbon atoms across the levels of biological organisation. *Journal of Biological Education*, *53*(1), 110–125. https://doi.org/10.1080/00219266.2018.1447002

Gladwell, V. F., Brown, D. K., Barton, J. L., Tarvainen, M. P., Kuoppa, P., Pretty, J., Suddaby, J. M., & Sandercock, G. R. H. (2012). The effects of views of nature on autonomic control. *European Journal of Applied Physiology*, *112*, 3379–3386.

Hartley, L. M., Wilke, B. J., Schramm, J. W., D'Avanzo, C., & Anderson, C. W. (2011). College students' understanding of the carbon cycle: Contrasting principle-based and informal reasoning. *BioScience*, *61*(1), 65–75.

Hůla, M., & Flegr, J. (2021). Habitat selection and human aesthetic responses to flowers. *Evolutionary Human Sciences*, *3*, e5.

Kaasinen, A. (2019). Plant Species Recognition Skills in Finnish Students and Teachers. *Education Sciences*, *9*(2), 85. https://doi.org/10.3390/educsci9020085

Karjalainen, E., Sarjala, T., & Raitio, H. (2010). Promoting human health through forests: Overview and major challenges. *Environmental Health and Preventive Medicine*, *15*(1), 1–8. https://doi.org/10.1007/s12199-008-0069-2

Kuo, F. E. (2001). Coping with poverty: Impacts of environment and attention in the inner city. *Environment and Behavior*, *33*(1), 5–34.

Lampert, P., Müllner, B., Pany, P., Scheuch, M., & Kiehn, M. (2020). Students' conceptions of plant reproduction processes. *Journal of Biological Education*, *54*(2), 213–223. https://doi.org/10.1080/00219266.2020.1739424

Laumann, K., Gärling, T., & Stormark, K. M. (2003). Selective attention and heart rate responses to natural and urban environments. *Journal of Environmental Psychology, Restorative Environments*, *23*(2), 125–134. https://doi.org/10.1016/S0272-4944(02)00110-X

Maller, C., Townsend, M., St Leger, L., Henderson-Wilson, C., Pryor, A., Prosser, L., & Moore, M. (2009). Healthy Parks, Healthy People: The Health Benefits of Contact with Nature in a Park Context. *The George Wright Forum*, *26*(2), 51–83.

Nyberg, E. (2017). Aesthetic Experiences Related to Living Plants: A Starting Point in Framing Humans' Relationship with Nature? In *Ethical Literacies and Education for Sustainable Development* (pp. 137–157). Palgrave Macmillan, Cham. https://doi.org/10.1007/978-3-319-49010-6_9

Pany, P., Meier, F. D., Dünser, B., Yanagida, T., Kiehn, M., & Möller, A. (2022). Measuring students' plant awareness: A prerequisite for effective botany education. *Journal of Biological Education*, *58*(5), 1103–1116. https://doi.org/10.1080/00219266.2022.2159491

Park, B. J., Tsunetsugu, Y., Kasetani, T., Kagawa, T., & Miyazaki, Y. (2010). The physiological effects of Shinrin-yoku (taking in the forest atmosphere or forest bathing): Evidence from field experiments in 24 forests across Japan. *Environmental Health and Preventive Medicine*, *15*(1), 18–26. https://doi.org/10.1007/s12199-009-0086-9

Schunko, C., & Brandner, A. (2022). Urban nature at the fingertips: Investigating wild food foraging to enable nature interactions of urban dwellers. *Ambio*, *51*(5), 1168–1178. https://doi.org/10.1007/s13280-021-01648-1

Schussler, E. E., & Olzak, L. A. (2008). It's not easy being green: Student recall of plant and animal images. *Journal of Biological Education*, *42*(3), 112–119.

Takayama, N., Korpela, K., Lee, J., Morikawa, T., Tsunetsugu, Y., Park, B.-J., Li, Q., Tyrväinen, L., Miyazaki, Y., & Kagawa, T. (2014). Emotional, restorative and vitalizing effects of forest and urban environments at four sites in Japan. *International Journal of Environmental Research and Public Health*, *11*(7), 7207–7230.

Taylor, A. F., Kuo, F. E., & Sullivan, W. C. (2001). Coping with ADD: The surprising connection to green play settings. *Environment and Behavior*, *33*(1), 54–77.

Urry, L. A., Cain, M. L., Wasserman, S. A., Minorsky, P. V., & Reece, J. B. (2017). *Campbell biology*. Pearson Education, Incorporated.
