# Supplementary material for: Rooting plant awareness: development of a tool to explore human-plant relationships
Source: PeerJ. 2026 Sep 8;14:e21667. doi: 10.7717/peerj.21667 (PMC13564396; doi:10.7717/peerj.21667)
Supplement: Supplementary file 11 [file peerj-14-21667-s013.pdf]

Table 2S: Results of the Confirmatory Factor Analysis for the three dimensions of plant awareness (*attention*, *knowledge*, *attitude*) as well as interest: estimates, standard errors, and confidence intervals. N=474

| | Factors | | | | | | | | Confidence Intervals | |
|---|---|---|---|---|---|---|---|---|---|---|
| Item | *Attention* | | *Knowledge* | | *Attitude* | | Interest | | | |
| | Estimate | Std. Err. | Estimate | Std. Err. | Estimate | Std. Err. | Estimate | Std. Err. | lower bound | upper bound |
| Apple Tree | 1.0000 | | | | | | | | | |
| Pear Tree | 2.5410 | 1.6820 | | | | | | | -0.75572 | 5.83772 |
| Nettle | 3.6920 | 2.3130 | | | | | | | -0.84148 | 8.22548 |
| Strawberries | 2.0550 | 1.4560 | | | | | | | -0.79876 | 4.90876 |
| Spruce | 2.9480 | 1.8030 | | | | | | | -0.58588 | 6.48188 |
| Cereal Field | 1.5290 | 0.9910 | | | | | | | -0.41336 | 3.47136 |
| Cherry | 2.8460 | 1.8120 | | | | | | | -0.70552 | 6.39752 |
| Maize Field | 0.0430 | 0.4450 | | | | | | | -0.8292 | 0.9152 |
| Fruit Tree | 1.1280 | 0.9320 | | | | | | | -0.69872 | 2.95472 |
| Sunflowers | 1.8050 | 1.1790 | | | | | | | -0.50584 | 4.11584 |
| K101_01_1 | | | 1 | | | | | | | |
| K101_01_2 | | | 0.795 | 0.143 | | | | | 0.51472 | 1.07528 |
| K101_01_3 | | | 0.815 | 0.19 | | | | | 0.4426 | 1.1874 |
| K101_01_4 | | | 0.787 | 0.143 | | | | | 0.50672 | 1.06728 |
| K101_02_1 | | | 0.507 | 0.137 | | | | | 0.23848 | 0.77552 |
| K101_02_2 | | | 1293 | 0.162 | | | | | 1292.68248 | 1293.31752 |
| K101_02_3 | | | 1441 | 0.169 | | | | | 1440.66876 | 1441.33124 |
| K101_02_4 | | | 1474 | 0.169 | | | | | 1473.66876 | 1474.33124 |
| K101_03_1 | | | 0.607 | 0.151 | | | | | 0.31104 | 0.90296 |
| K101_03_2 | | | 0.653 | 0.146 | | | | | 0.36684 | 0.93916 |
| K101_03_3 | | | 0.863 | 0.149 | | | | | 0.57096 | 1.15504 |
| K101_03_4 | | | 0.669 | 0.133 | | | | | 0.40832 | 0.92968 |
| K101_04_1 | | | 0.765 | 0.145 | | | | | 0.4808 | 1.0492 |

| | Factors | | | | | | | | Confidence Intervals | |
|---|---|---|---|---|---|---|---|---|---|---|
| Item | *Attention* | | *Knowledge* | | *Attitude* | | Interest | | | |
| | Estimate | Std. Err. | Estimate | Std. Err. | Estimate | Std. Err. | Estimate | Std. Err. | lower bound | upper bound |
| K101_04_2 | | | 1007 | 0.148 | | | | | 1006.70992 | 1007.29008 |
| K101_04_3 | | | 0.84 | 0.134 | | | | | 0.57736 | 1.10264 |
| K101_04_4 | | | 0.667 | 0.139 | | | | | 0.39456 | 0.93944 |
| K101_05_1 | | | 1283 | 0.157 | | | | | 1282.69228 | 1283.30772 |
| K101_05_2 | | | 0.983 | 0.164 | | | | | 0.66156 | 1.30444 |
| K101_05_3 | | | 1518 | 0.193 | | | | | 1517.62172 | 1518.37828 |
| K101_05_4 | | | 1051 | 0.159 | | | | | 1050.68836 | 1051.31164 |
| K101_06_1 | | | 0.762 | 0.151 | | | | | 0.46604 | 1.05796 |
| K101_06_2 | | | 0.999 | 0.162 | | | | | 0.68148 | 1.31652 |
| K101_06_3 | | | 1356 | 0.176 | | | | | 1355.65504 | 1356.34496 |
| K101_06_4 | | | 0.989 | 0.167 | | | | | 0.66168 | 1.31632 |
| K101_07_1 | | | 0.837 | 0.161 | | | | | 0.52144 | 1.15256 |
| K101_07_2 | | | 1369 | 0.177 | | | | | 1368.65308 | 1369.34692 |
| K101_07_3 | | | 1492 | 0.189 | | | | | 1491.62956 | 1492.37044 |
| K101_07_4 | | | 1363 | 0.18 | | | | | 1362.6472 | 1363.3528 |
| K101_08_1 | | | -0.079 | 0.18 | | | | | -0.4318 | 0.2738 |
| K101_08_2 | | | 1355 | 0.171 | | | | | 1354.66484 | 1355.33516 |
| K101_08_3 | | | 1384 | 0.207 | | | | | 1383.59428 | 1384.40572 |
| K101_08_4 | | | 0.941 | 0.154 | | | | | 0.63916 | 1.24284 |
| K209_01 | | | 0.507 | 0.143 | | | | | 0.22672 | 0.78728 |
| K209_02 | | | 0.768 | 0.169 | | | | | 0.43676 | 1.09924 |
| K209_03 | | | 2045 | 0.237 | | | | | 2044.53548 | 2045.46452 |
| K209_04 | | | 1819 | 0.215 | | | | | 1818.5786 | 1819.4214 |
| K209_05 | | | 1260 | 0.174 | | | | | 1259.65896 | 1260.34104 |
| K209_06 | | | 1406 | 0.173 | | | | | 1405.66092 | 1406.33908 |
| K205_01 | | | 0.799 | 0.145 | | | | | 0.5148 | 1.0832 |
| K205_02 | | | 1419 | 0.191 | | | | | 1418.62564 | 1419.37436 |
| K205_03 | | | 1100 | 0.16 | | | | | 1099.6864 | 1100.3136 |

| | Factors | | | | | | | | Confidence Intervals | |
|---|---|---|---|---|---|---|---|---|---|---|
| Item | *Attention* | | *Knowledge* | | *Attitude* | | Interest | | | |
| | Estimate | Std. Err. | Estimate | Std. Err. | Estimate | Std. Err. | Estimate | Std. Err. | lower bound | upper bound |
| K205_04 | | | 1443 | 0.183 | | | | | 1442.64132 | 1443.35868 |
| K205_05 | | | 1091 | 0.182 | | | | | 1090.64328 | 1091.35672 |
| K206_03 | | | 1136 | 0.176 | | | | | 1135.65504 | 1136.34496 |
| K206_01 | | | 1269 | 0.18 | | | | | 1268.6472 | 1269.3528 |
| K206_02 | | | 1070 | 0.165 | | | | | 1069.6766 | 1070.3234 |
| K206_04 | | | 0.521 | 0.143 | | | | | 0.24072 | 0.80128 |
| K207_01 | | | 1114 | 0.174 | | | | | 1113.65896 | 1114.34104 |
| K207_02 | | | 0.939 | 0.16 | | | | | 0.6254 | 1.2526 |
| E101 | | | 0.412 | 0.167 | | | | | 0.08468 | 0.73932 |
| E113 | | | 0.259 | 0.193 | | | | | -0.11928 | 0.63728 |
| E103 | | | 0.539 | 0.14 | | | | | 0.2646 | 0.8134 |
| E104_01 | | | 1259 | 0.17 | | | | | 1258.6668 | 1259.3332 |
| E104_02 | | | 1199 | 0.172 | | | | | 1198.66288 | 1199.33712 |
| E104_03 | | | 1162 | 0.183 | | | | | 1161.64132 | 1162.35868 |
| E104_04 | | | 1347 | 0.2 | | | | | | |
| E104_05 | | | 0.931 | 0.217 | | | | | 0.50568 | 1.35632 |
| E117_01_korrkt | | | 0.534 | 0.171 | | | | | 0.19884 | 0.86916 |
| E118_01_korrkt | | | 0.430 | 0.218 | | | | | 0.00272 | 0.85728 |
| E203 | | | 0.435 | 0.134 | | | | | | |
| E206 | | | 0.653 | 0.136 | | | | | | |
| E205 | | | 0.495 | 0.127 | | | | | | |
| E204 | | | 0.46 | 0.126 | | | | | | |
| AT01_01 | | | | | 1 | | | | | |
| AT06_01 | | | | | 0.496 | 0.081 | | | 0.33724 | 0.65476 |
| AT03_01 | | | | | 0.959 | 0.08 | | | 0.8022 | 1.1158 |
| AT04_01 | | | | | 0.687 | 0.077 | | | 0.53608 | 0.83792 |
| AT05_01 | | | | | 0.829 | 0.083 | | | 0.66632 | 0.99168 |

| | Factors | | | | | | | | Confidence Intervals | |
|---|---|---|---|---|---|---|---|---|---|---|
| Item | *Attention* | | *Knowledge* | | *Attitude* | | Interest | | | |
| | Estimate | Std. Err. | Estimate | Std. Err. | Estimate | Std. Err. | Estimate | Std. Err. | lower bound | upper bound |
| PI01_01 | | | | | | | 1000 | | | |
| PI01_02 | | | | | | | 1013 | 0.022 | 1012.95688 | 1013.04312 |
| PI01_03 | | | | | | | 0.941 | 0.024 | 0.89396 | 0.98804 |
| PI01_04 | | | | | | | 0.887 | 0.024 | 0.83996 | 0.93404 |
| PI01_05 | | | | | | | 0.891 | 0.024 | 0.84396 | 0.93804 |
| PI01_06 | | | | | | | 1043 | 0.019 | 0.86388 | 0.95012 |
| PI01_07 | | | | | | | 0.958 | 0.022 | 1039.95688 | 1040.04312 |
| PI01_08 | | | | | | | 0.956 | 0.022 | 0.89284 | 0.97516 |
| PI01_09 | | | | | | | 0.907 | 0.021 | 0.90076 | 0.97524 |
| PI01_10 | | | | | | | 1040 | 0.019 | 0.749 | 0.847 |
| PI02_01 | | | | | | | 0.934 | 0.025 | 0.929 | 1.027 |
| PI02_02 | | | | | | | 0.938 | 0.025 | 1042.94316 | 1043.05684 |
| PI02_03 | | | | | | | 0.798 | 0.029 | | |
| PI02_04 | | | | | | | 0.978 | 0.026 | | |

Estimates which are not significant on the 5%-level are printed as italics
