# Supplementary material for: Rooting plant awareness: development of a tool to explore human-plant relationships
Source: PeerJ. 2026 Sep 8;14:e21667. doi: 10.7717/peerj.21667 (PMC13564396; doi:10.7717/peerj.21667)
Supplement: Supplementary file 12 [file peerj-14-21667-s014.pdf]

Table 3S: Reliability of knowledge and its subscales (Cronbach's α)

**Dimension**	**Subscale**	**Cronbach's α**	**Confidence Intervalls**
Knowledge		0.96	0.94; 0.97
	Plant Species Identification	0.80	0.27; 0.98
	Practical Knowledge	0.73	0; 0.98
	Spice Plants	0.93	0.74; 0.99
	Plants as Living Organisms	0.94	0.91; 0.97
	Carbon Cycle	0.79	0.36; 0.97
	Agriculture	0.86	0.47; 0.98
	Sustainability	0.83	0.46; 0.97
