# Supplementary material for: Rooting plant awareness: development of a tool to explore human-plant relationships
Source: PeerJ. 2026 Sep 8;14:e21667. doi: 10.7717/peerj.21667 (PMC13564396; doi:10.7717/peerj.21667)
Supplement: Supplementary file 13 [file peerj-14-21667-s015.pdf]

Table 4S: Exact p-values for the post hoc analysis via pairwise Mann-Whitney tests (with Holm correction) of the mean comparison of the three dimensions of plant awareness *attention*, *knowledge*, and *attitude*, as well as interest between the four subsamples

	Attention			*Knowledge*			*Attitude*			Interest		
	Gymnasium	*Naturpark-schule*	Experts	*Gymnasium*	*Naturpark-schule*	Experts	*Gymnasium*	*Naturpark-schule*	Experts	*Gymnasium*	*Naturpark-schule*	Experts
Naturparkschule	0.79	-	-	0.32	-	-	0.01	-	-	2e-16	-	-
Experts	6.1e-11	5.0e-12	-	2e-16	2.4e-16	-	3.1e-08	3.2e-06	-	2e-16	4.7e-12	-
Mitttelschule	0.76	0.79	2.2e-08	1.5e-08	5.4e-10	3.7e-13	0.86	0.30	3.3e-05	0.0018	2e-16	7.5e-13
