# Supplementary material for: Rooting plant awareness: development of a tool to explore human-plant relationships
Source: PeerJ. 2026 Sep 8;14:e21667. doi: 10.7717/peerj.21667 (PMC13564396; doi:10.7717/peerj.21667)
Supplement: Supplementary file 14 [file peerj-14-21667-s016.pdf]

Table 5S: Means, Standard Deviations, Standard Errors, and confidence intervals of the three dimensions of plant awareness (*attention*, *knowledge*, *attitudes*), as well as interest for the four subsamples *Mittelschule, Gymnasium, Naturparkschule,* and experts

			Attention			
Subsample	N	Mean	SD	s.e.	CI lower bound	CI upper bound
Mittelschule	66	0.59	0.84	0.11	0.37	0.81
Gymnasium	370	0.82	1.15	0,06	0.69	0.93
Naturparkschule	388	0.76	1.05	0,05	0.64	0.84
Experts	34	2.41	1.79	0,20	1.72	2.50
			Knowledge			
	N	Mean	SD	s.e.	CI lower bound	CI upper bound
Mittelschule	66	36.09	11.5	2,69	36.11	46.65
Gymnasium	370	46.28	10.72	0,86	50.98	54.36
Naturparkschule	388	47.31	10.21	0,80	50.52	53.66
Experts	34	66.18	6.35	1,17	66.34	70.92
			Attitudes			
	N	Mean	SD	s.e.	CI lower bound	CI upper bound
Mittelschule	66	19.29	3.67	0,11	19.07	19.51
Gymnasium	370	19.10	3.67	0,06	18.98	19.22
Naturparkschule	388	20.04	3.14	0,06	19.92	20.16
Experts	34	22.74	1.93	0,22	22.31	23.17
			Interest			
	N	Mean	SD	s.e.	CI lower bound	CI upper bound
Mittelschule	66	18.82	10.28	1.45	15.98	21.66
Gymnasium	370	25.21	12.24	0.71	23.82	26.60
Naturparkschule	388	38.75	7.51	0.42	37.93	39.57
Experts	34	49.13	4.97	0.91	47.35	50.91
