# Supplementary material for: Rooting plant awareness: development of a tool to explore human-plant relationships
Source: PeerJ. 2026 Sep 8;14:e21667. doi: 10.7717/peerj.21667 (PMC13564396; doi:10.7717/peerj.21667)
Supplement: Supplementary file 15 [file peerj-14-21667-s017.pdf]

Table 6S: Means and standard deviations of the seven subscales of the plant awareness dimension *knowledge,* as well as effect sizes (ordinal η^2) and p-values for the four subsamples *Mittelschule, Gymnasium, Naturparkschule,* and experts

Subscale		*Living organisms*		*Carbon Cycle*		*Agriculture*		*Sustainability*		*Identification*		*Practical*		*Spices*	
	N	Mean	SD	Mean	SD	Mean	SD	Mean	SD	Mean	SD	Mean	SD	Mean	SD
Mittelschule	66	11.91	6.13	1.75	1.84	2.08	1.59	2.20	1.56	1.23	1.23	11.98	4.04	2.75	1.33
Gymnasium	370	18.49	5.76	3.28	1.86	3.18	1.77	3.25	1.87	2.84	1.03	11.16	3.51	3.83	1.21
Naturparkschule	388	14.64	7.17	3.84	1.64	2.71	1.70	2.96	1.80	1.14	1.21	13.56	3.62	3.65	1.61
Experts	34	25.79	6.21	6.12	0.99	4.56	1.05	5.71	1.09	4.97	0.17	12.85	4.32	5.00	(0.00)
Kruskal Wallis test															
Ordinal η^2 all groups		0.157		0.15		0.077		0.0972		0.16		0.073		0.0847	
Ordinal η^2 between schools		0.1		0.0825		0.0367		0.0215		0.0597		0.0762		0.0395	
Ordinal η^2 students vs. experts		0.0654		0.0751		0.0419		0.0774		0.107		(0)		0.0472	
p-value		2.2E-16		2.2E-16		8.02E-15		2.2E-16		2.2E-16		4.30E-14		3.08E-16	
H (χ^2)		137		130.91		68.721		85.999		139.99		65.134		75.331	
df		3		3		3		3		3		3		3	
