# Supplementary material for: Rooting plant awareness: development of a tool to explore human-plant relationships
Source: PeerJ. 2026 Sep 8;14:e21667. doi: 10.7717/peerj.21667 (PMC13564396; doi:10.7717/peerj.21667)
Supplement: Supplementary file 16 [file peerj-14-21667-s018.pdf]

Table 7S: Regressions with confidence intervals and effect sizes

**Predictor**	**Attitude**				**Knowledge**				**Attention**				**Interest**			
	Estimate	s.e.	p	95% CI	Estimate	s.e.	p	95% CI	Estimate	s.e.	p	95% CI	Estimate	s.e.	p	95% CI
Age	-0.37	0.12	0.001	[-0.61, -0.13]	0.01	0.01	0.062	[-0.01, 0.03]	0.16	0.03	<0.001	[0.10, 0.22]	-2.73	0.33	<0.001	[-3.38, -2.08]
Gender	-1.05	0.28	<0.001	[-1.60, -0.50]	-0.007	0.01	0.569	[-0.03, 0.01]	-0.23	0.08	0.006	[-0.39, -0.07]	-2.37	0.79	0.003	[-3.92, -0.82]
Naturparkschule	-0.18	0.69	0.796	[-1.53, 1.17]	-0.03	0.03	0.286	[-0.09, 0.03]	0.21	0.20	0.292	[-0.18, 0.60]	5.27	1.92	0.006	[1.51, 9.03]
Mittelschule	0.56	0.54	0.300	[-0.50, 1.62]	-0.10	0.03	<0.001	[-0.16, -0.04]	-0.19	0.16	0.233	[-0.50, 0.12]	-6.69	1.57	<0.001	[-9.77, -3.61]
