# Supplementary material for: Rooting plant awareness: development of a tool to explore human-plant relationships
Source: PeerJ. 2026 Sep 8;14:e21667. doi: 10.7717/peerj.21667 (PMC13564396; doi:10.7717/peerj.21667)
Supplement: Supplementary file 17 [file peerj-14-21667-s019.pdf]

Schau dir das Wimmelbild genau an! Nach 30 Sekunden erscheint ein Weiter-Button. Klicke ihn an, wenn du bereit bist. Sonst wirst du nach 60 Sekunden automatisch weitergeleitet.

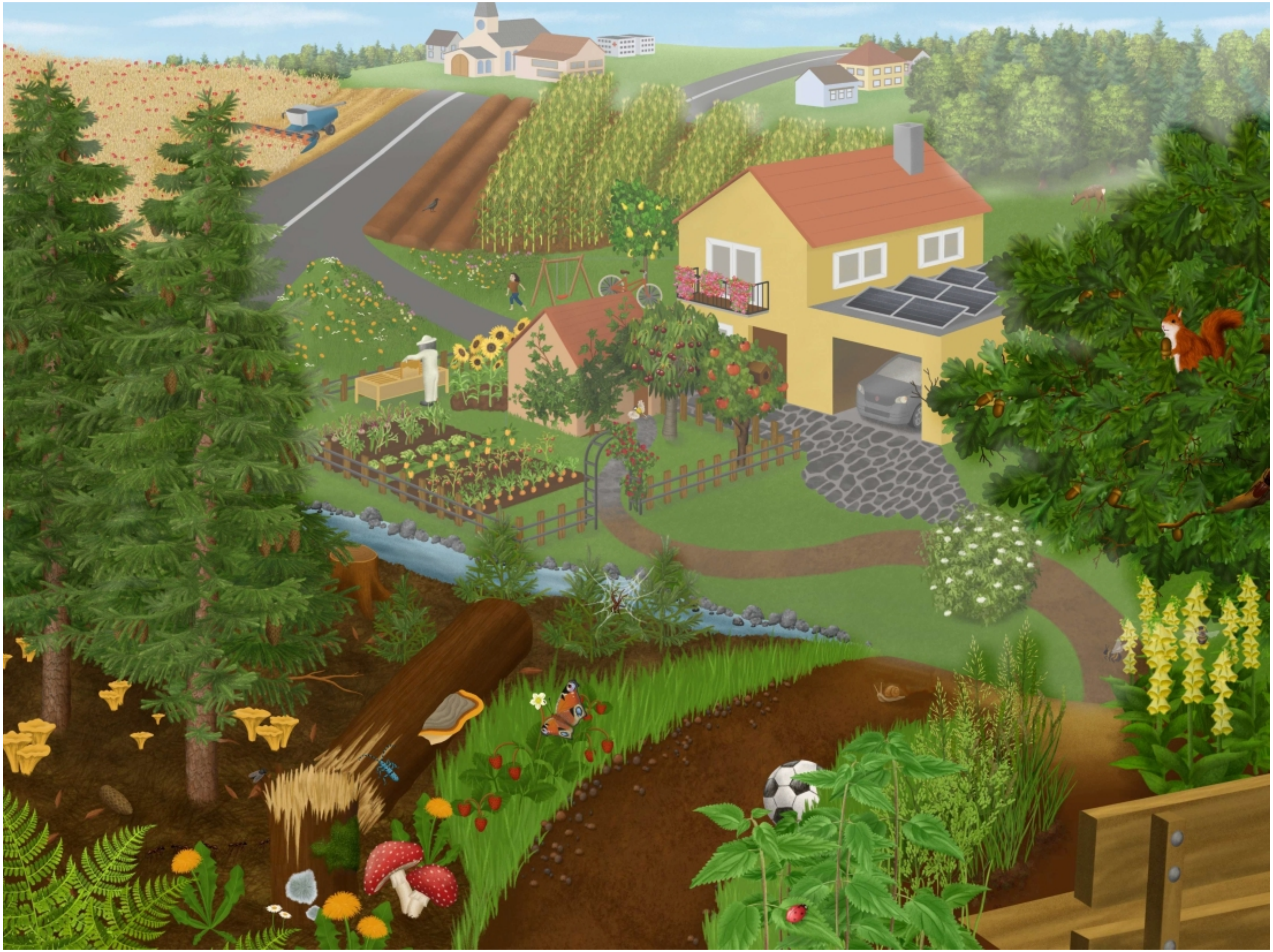

Was hast du auf dem Wimmelbild gesehen? Notiere alle Dinge in der Tabelle, an die du dich erinnern kannst (du hast Platz für höchstens 25 Dinge)!

Wenn du eine Antwort in ein Feld geschrieben hast und auf die E NTER-Taste drückst, erscheint automatisch ein neues Feld.

Welche der genannten Pflanzen werden häufig als Gewürze in der Küche verwendet?

Kreuze die richtigen Antworten an. Mehrere sind richtig.

- ☐ Thuje
- ☐ Pfeffer
- ☐ Thymian
- ☐ Oregano
- ☐ Majoran
- ☐ Löwenzahn
- ☐ Petunie
- ☐ Basilikum
- ☐ Philodendron
- ☐ Geranie

Die Pflanze auf dem Bild ist ein/e

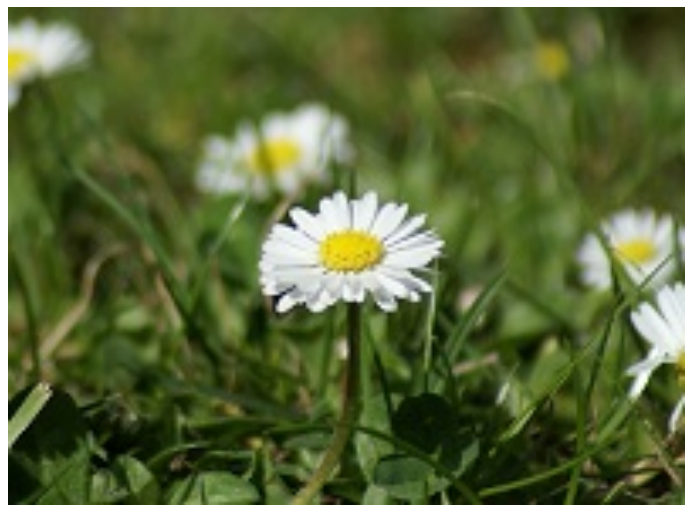

Kreuze die richtige Antwort an.

- ◯ Wiesenklee
- ◯ Margerite
- ◯ Löwenzahn
- ◯ Gänseblümchen

Hast du diese Pflanze schon einmal gesammelt oder gepflückt ?

Wähle aus was für dich zutrifft

Noch nie	1 – 5 mal pro Jahr	mehr als 5 mal pro Jahr	(Fast) Immer wenn diese Pflanze blüht
◯	◯	◯	◯

Welches der folgenden Bilder zeigt einen Löwenzahn?

Wähle das passende Bild aus.

○ 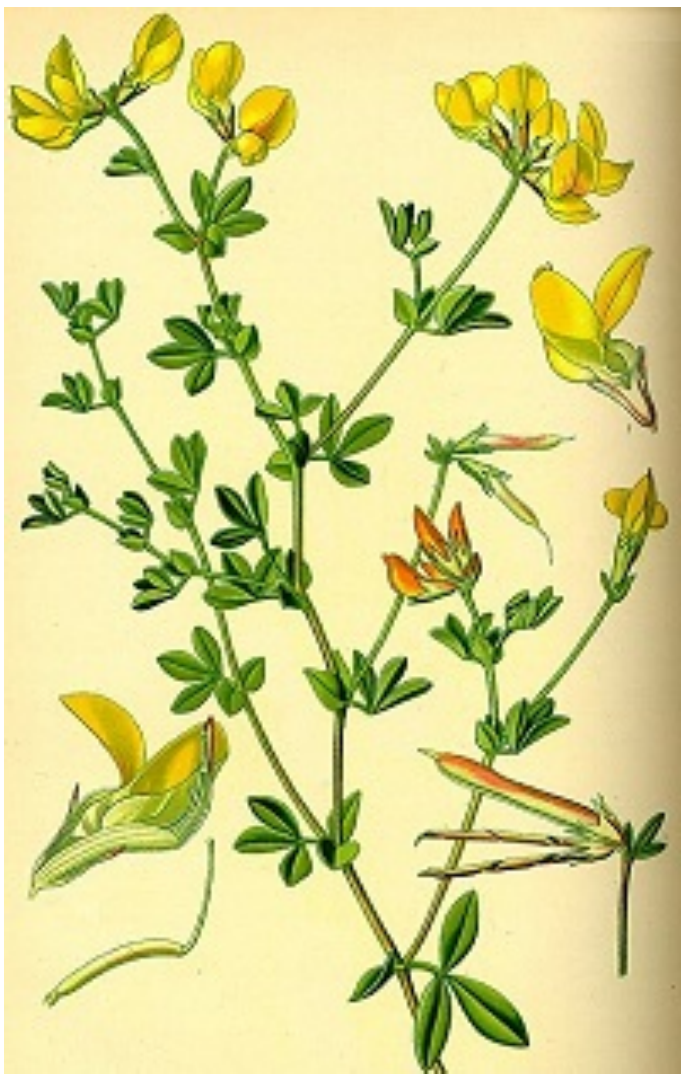

○ 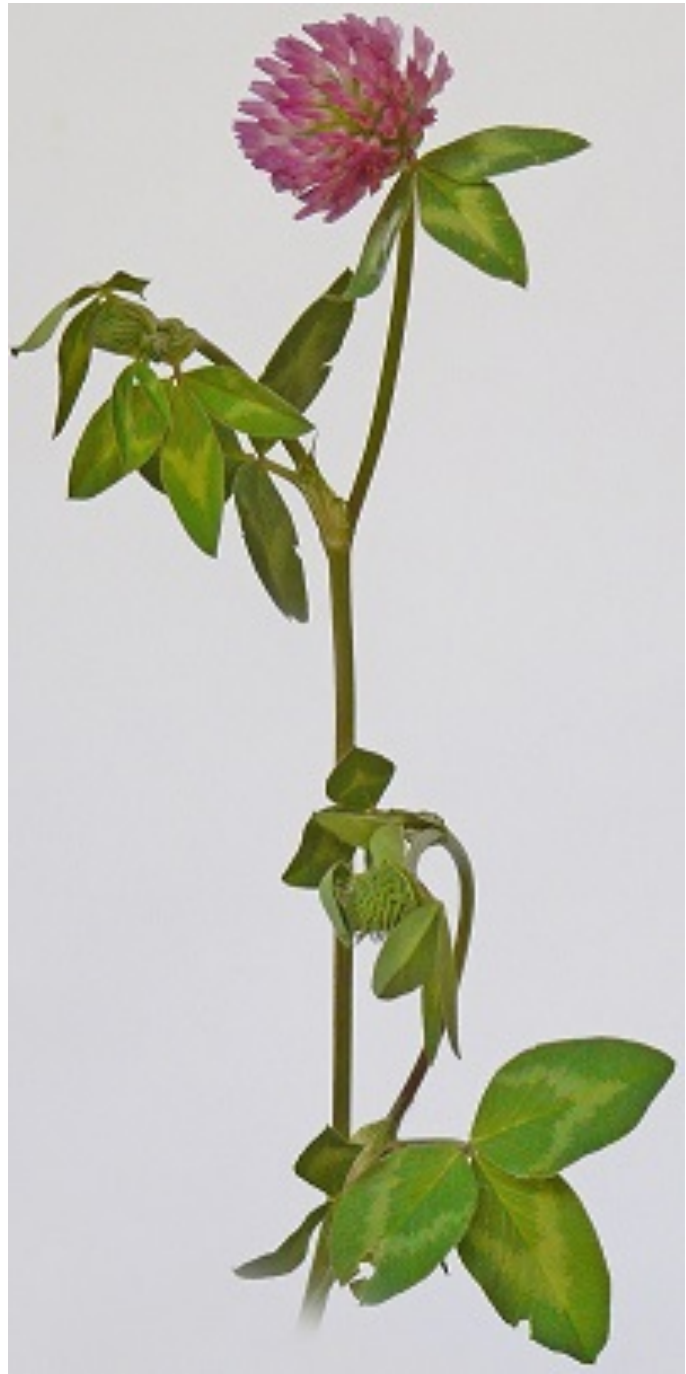

○ 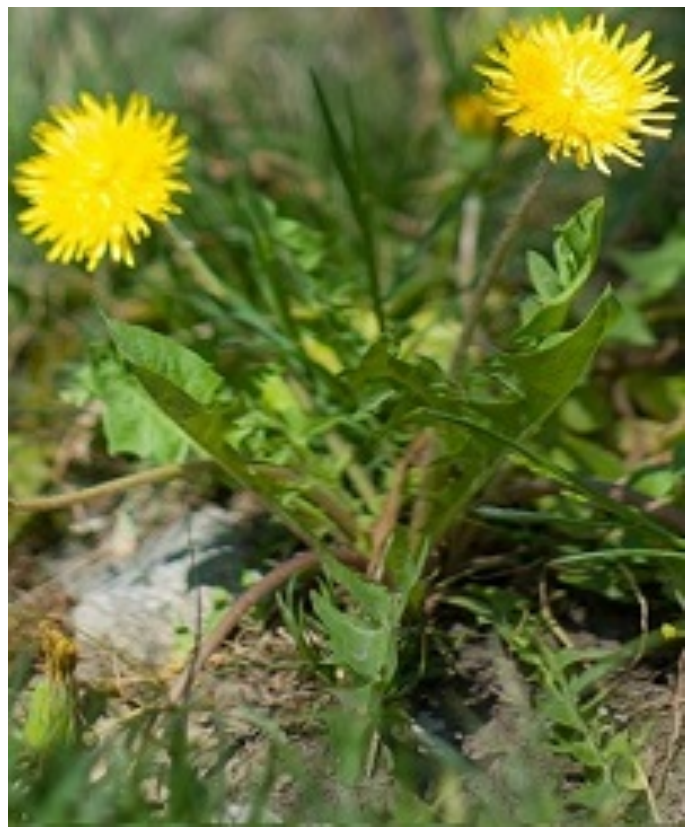

○ 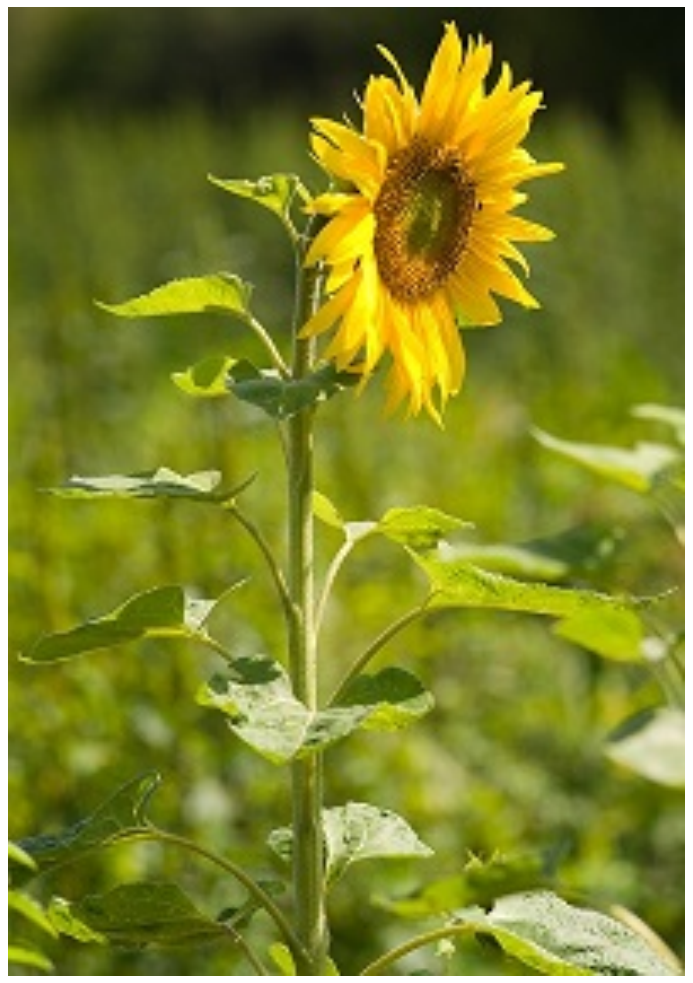

Hast du schon einmal einen Löwenzahn gesammelt oder gepflückt?

Wähle aus was für dich zutrifft

Noch nie	1 – 5 mal pro Jahr	mehr als 5 mal pro Jahr	(Fast) Immer wenn diese Pflanze blüht
○	○	○	○

Welcher dieser Bäume ist eine Fichte?

Wähle das passende Bild aus.

○ 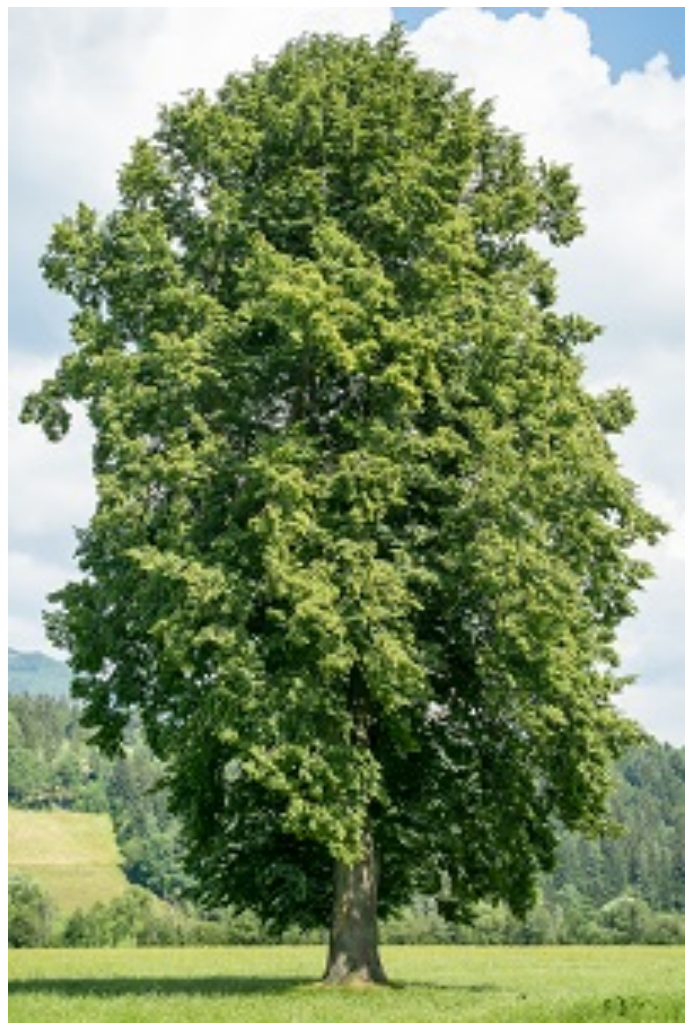

○ 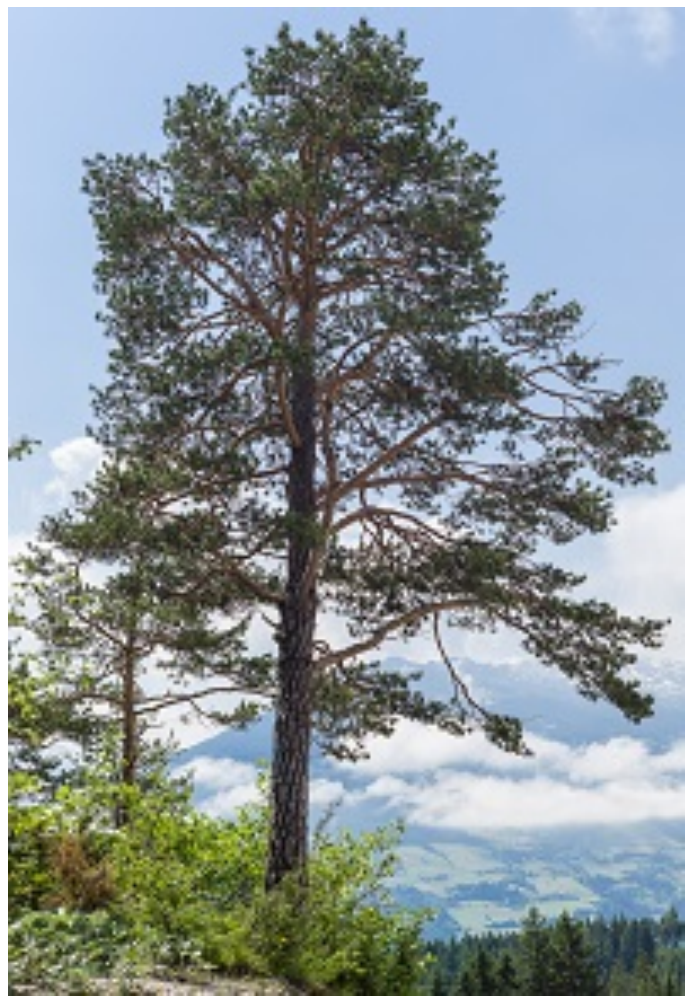

○ 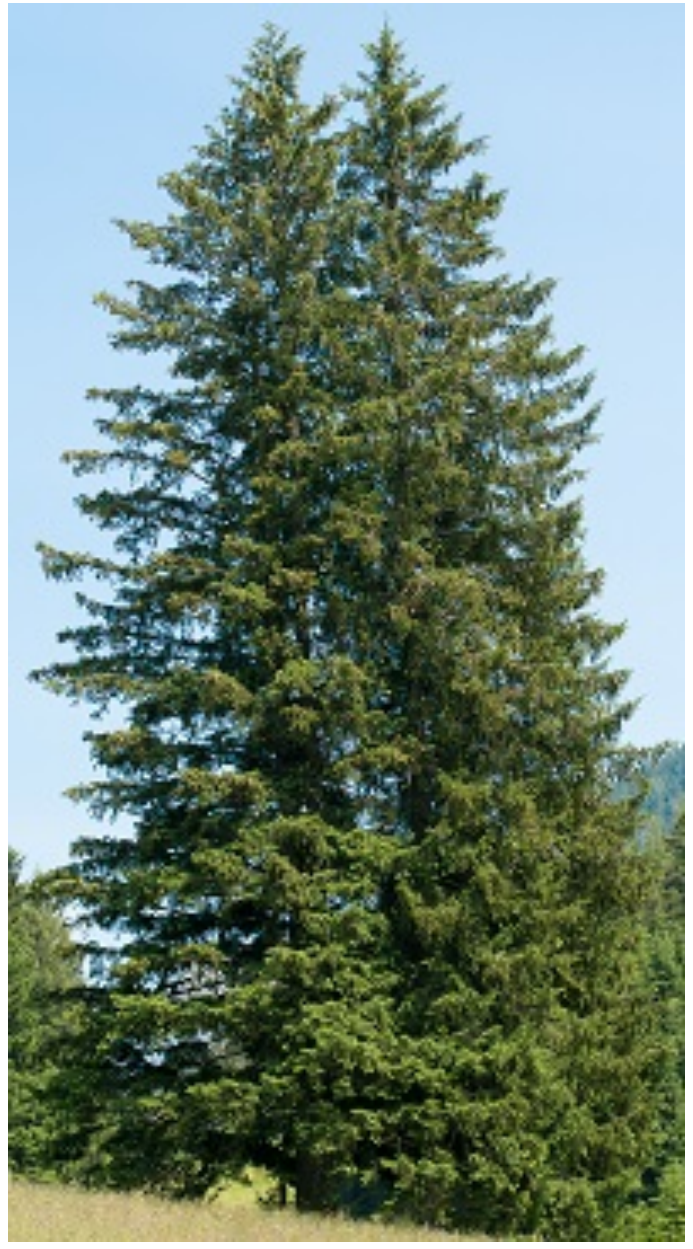

○ 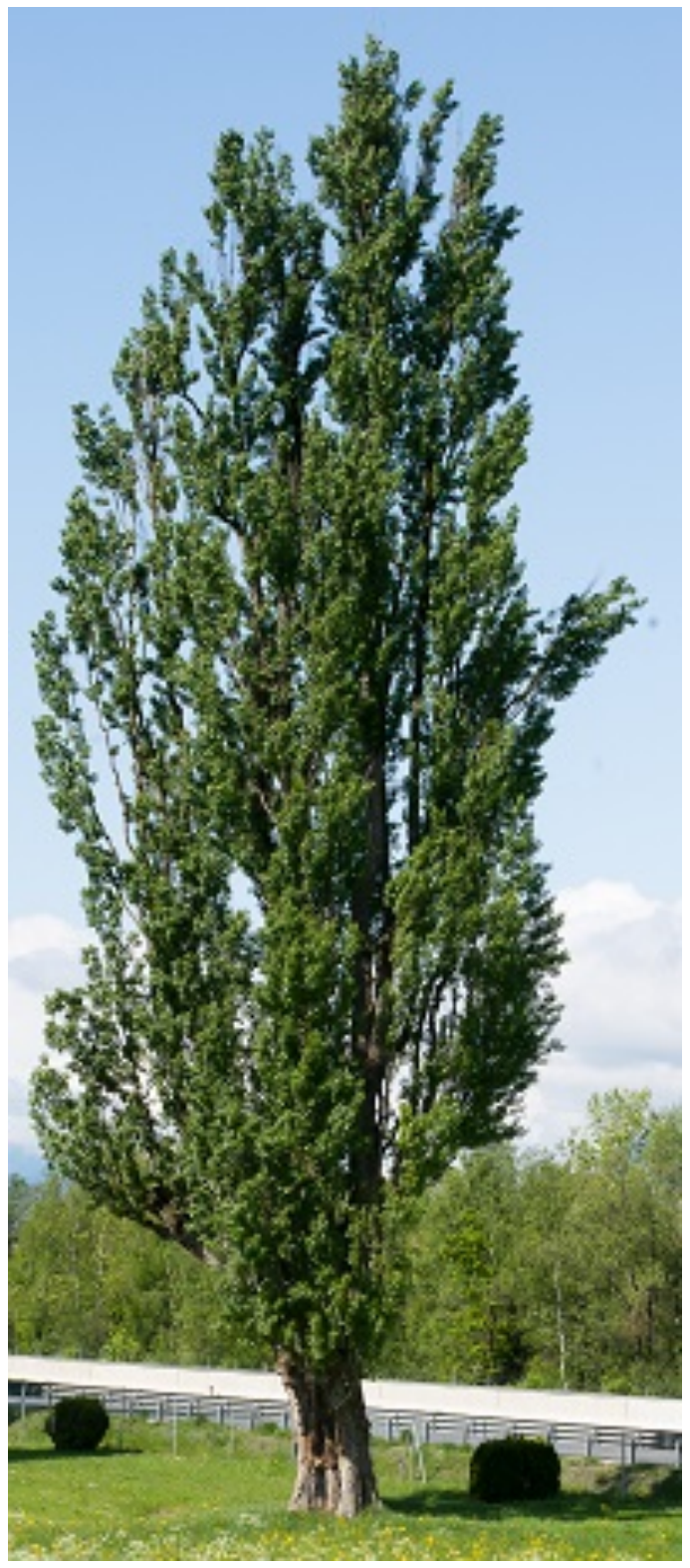

Welche Pflanze siehst du auf diesem Bild?

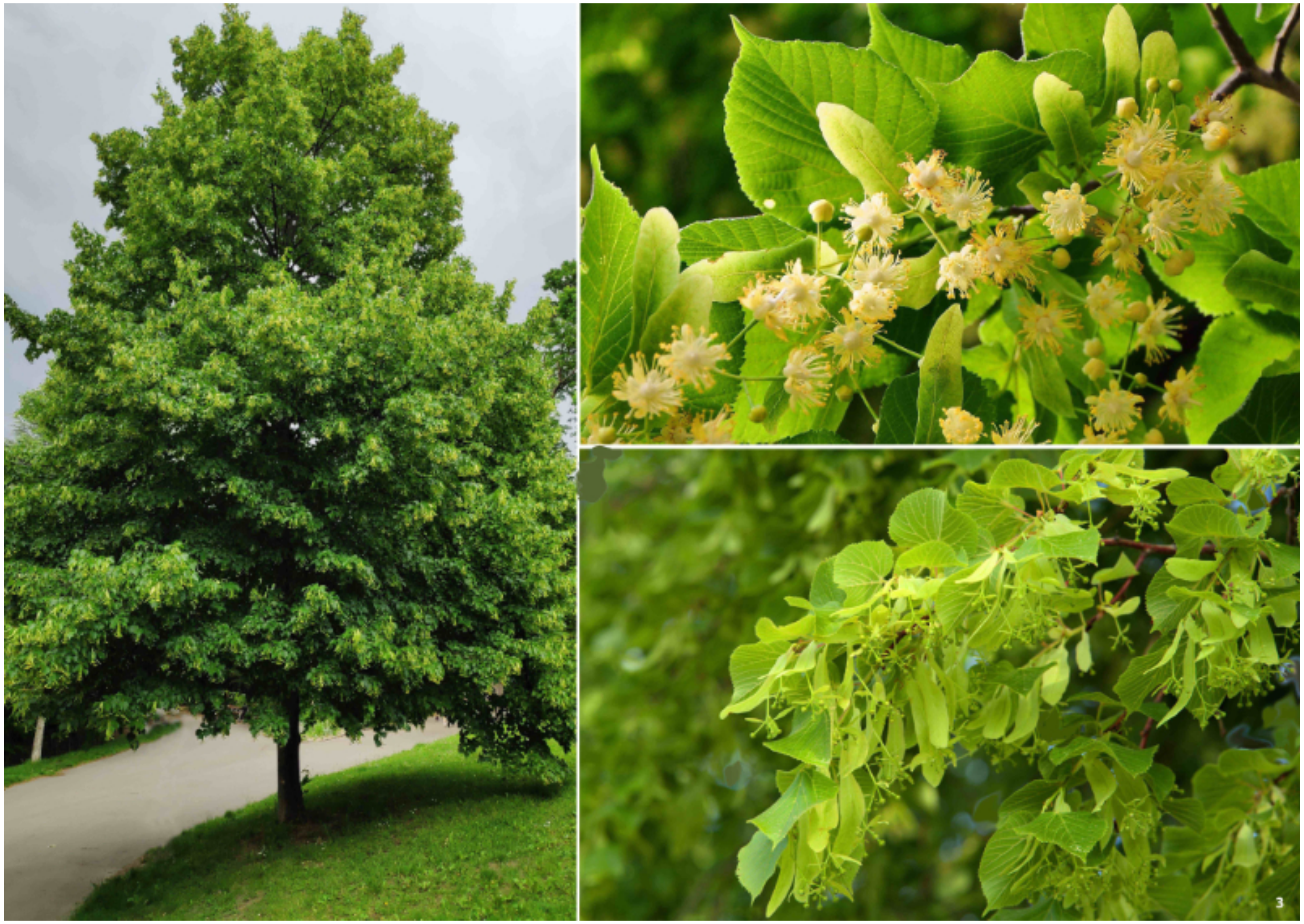

Kreuze die richtige Antwort an!

- ◯ Holler/Holunder
- ◯ Buche
- ◯ Linde
- ◯ Eiche
- ◯ Ahorn

Hast du Teile dieser Pflanze schon einmal gesammelt oder gepflückt?

Wähle aus was für dich zutrifft

Noch nie	1 – 5 mal pro Jahr	mehr als 5 mal pro Jahr	(Fast) Immer wenn diese Pflanze blüht
○	○	○	○

Welche Pflanze siehst du auf diesem Bild?

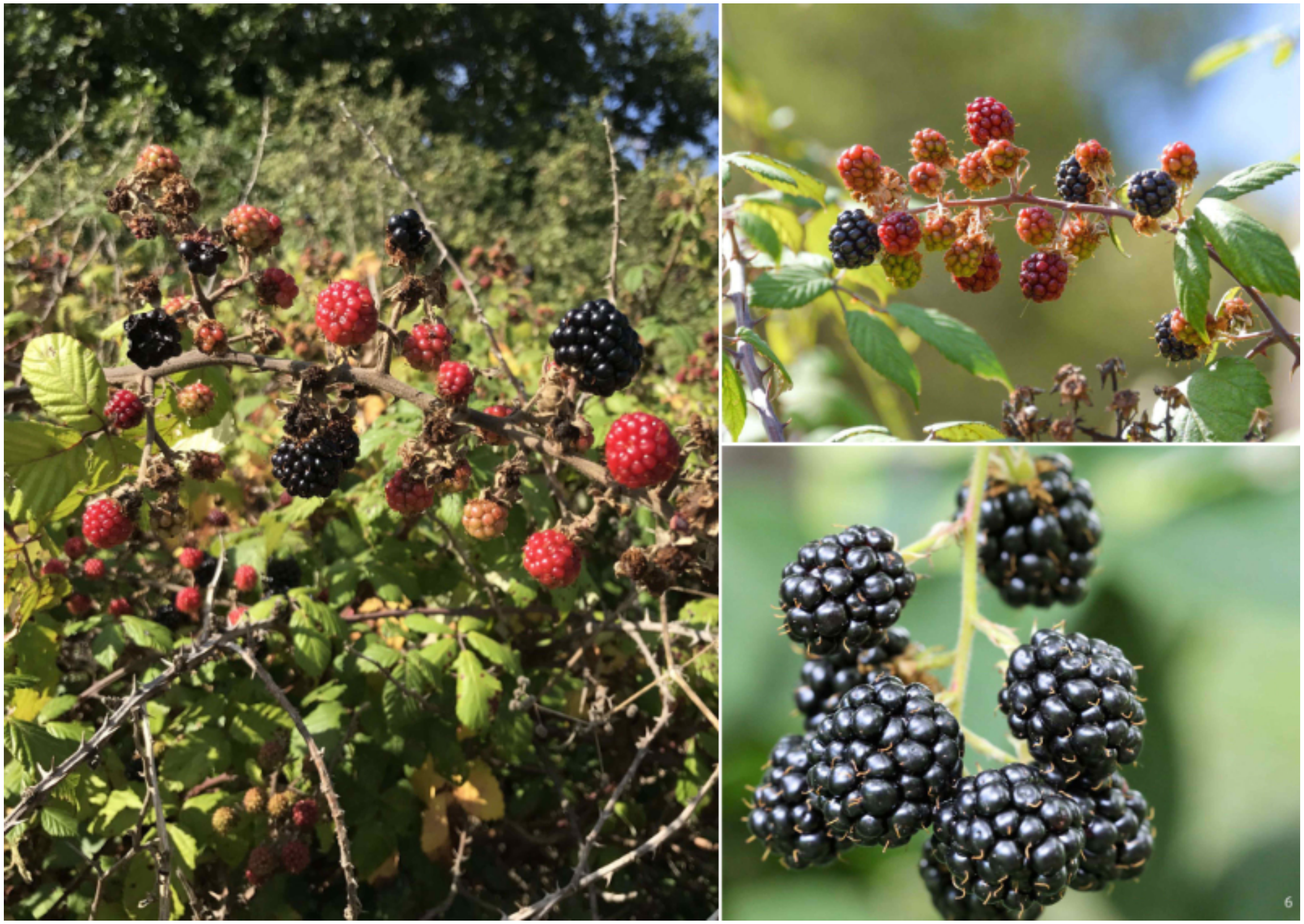

Kreuze die richtige Antwort an!

- ◯ Maulbeere
- ◯ Heidelbeere
- ◯ Brombeere
- ◯ Himbeere
- ◯ Johannisbeere

Hast du die Frucht dieser Pflanze schon einmal selber gesammelt oder gepflückt ?

Wähle aus was für dich zutrifft

Noch nie	1 – 5 mal pro Jahr	mehr als 5 mal pro Jahr	(Fast) Immer wenn diese Pflanze Früchte trägt
○	○	○	○

Hast Du schon einmal einen heilenden Tee getrunken (z.B. gegen Halsschmerzen, Husten oder Bauchschmerzen)?

Wenn Ja, wähle jene Pflanzen aus, aus denen du schon einen Tee gemacht hast.

- ☐ Thymian
- ☐ Lindenblüten
- ☐ Kamille
- ☐ Ingwer
- ☐ Fenchel
- ☐ Salbei
- ☐ Minze
- ☐ Brennessel
- ☐ Kümmel
- ☐ Efeu
- ☐ Eibisch
- ☐ andere ______

- ☐ Nein
- ☐ weiß ich nicht

Entscheide für welche der angeführten Gruppen – Pilze, Pflanzen, Tiere – die jeweiligen Aussagen zutreffen

Es können mehrere Gruppen pro Frage ausgewählt werden. Wähle die richtige(n) Antwort(en).

	Pilze	Pflanzen	Tiere	trifft auf alle Gruppen zu	ich verstehe diese Frage nicht	ich weiß nicht
Können sich auf verschiedene Weise bewegen.	☐	☐	☐	☐	☐	☐
Sind zum Wachstum fähig.	☐	☐	☐	☐	☐	☐
Geben Eigenschaften an ihre Nachkommen weiter.	☐	☐	☐	☐	☐	☐
Können krank werden.	☐	☐	☐	☐	☐	☐
Bestehen aus einer oder mehreren Zellen.	☐	☐	☐	☐	☐	☐
Pflanzen sich selbstständig fort (vermehren sich selbstständig).	☐	☐	☐	☐	☐	☐
Nehmen Nährstoffe auf, wandeln diese um und geben nicht benötigte Stoffe wieder ab.	☐	☐	☐	☐	☐	☐
Reagieren auf ihre Umwelt (sind reizbar).	☐	☐	☐	☐	☐	☐

Welche der angegebenen Punkte beeinflussen die Menge an Treibhausgasen (z.B. Kohlendioxid oder Methan) in der Atmosphäre direkt?

Kreuze die richtigen Antworten an. Mehrere Antworten sind möglich.

- ☐ Wälder
- ☐ Autoverkehr
- ☐ Meeresströmungen (z.B. der Golfstrom)
- ☐ Sonnenaktivität
- ☐ Ölkraftwerke
- ☐ Weidewirtschaft (z.B. Kühe)
- ☐ Flugverkehr
- ☐ Algen im Meer

- ☐ Ich verstehe die Frage nicht.

Welche der folgenden Punkte könnten in Zukunft die Produktion pflanzlicher Nahrungsmittel verringern?

Kreuze die richtigen Antworten an. Mehrere Antworten sind möglich.

- [ ] Erhöhte Temperaturen
- [ ] Zerstörung der Lebensräume
- [ ] Extremwetterereignisse
- [ ] Das Insektensterben
- [ ] Die Qualität der Böden

- [ ] Ich verstehe die Frage nicht.

Welche der folgenden Kleidungsstücke bestehen aus nachwachsenden Rohstoffen?
Kreuze die richtigen Antworten an. Mehrere Antworten sind möglich.

- ☐ Hemd aus Leinen
- ☐ Laufshirt aus Polyester
- ☐ Socken aus Bambus
- ☐ Hose aus Hanf
- ☐ T-Shirt aus Baumwolle

- ☐ Ich verstehe die Frage nicht.

Welche der folgenden Energiequellen werden aus nachwachsenden Rohstoffen gewonnen?
Kreuze die richtigen Antworten an. Mehrere Antworten sind möglich.

- ☐ Erdgas
- ☐ Holz
- ☐ Bio-Diesel
- ☐ Kohle
- ☐ Erdöl

- ☐ Ich verstehe die Frage nicht.

Wenn Pflanzen in meiner Umgebung sind fühle ich mich ....
Wähle den Wert, der für dich zutrifft.

sehr unwohl (schlecht) ○○○○○ sehr wohl (gut)

Ich finde (die meisten) Pflanzen .....
Wähle den Wert, der für dich zutrifft.

gar nicht schön ○○○○○ sehr schön

In meiner Familie spielen Pflanzen bei Festen ..... Rolle (z.B. als Schmuck)
Wähle den Wert, der für dich zutrifft.

gar keine ○○○○○ eine ganz wichtige

Ich hätte gerne .... Bäume in meiner Stadt.
Wähle den Wert, der für dich zutrifft.

viel weniger ○○○○○ viel mehr

Ich hätte gerne .... Pflanzen in meiner direkten Umgebung (z.B. Zuhause oder in der Schule).
Wähle den Wert, der für dich zutrifft.

viel weniger ○○○○○ viel mehr
