# Supplementary material for: Rooting plant awareness: development of a tool to explore human-plant relationships
Source: PeerJ. 2026 Sep 8;14:e21667. doi: 10.7717/peerj.21667 (PMC13564396; doi:10.7717/peerj.21667)
Supplement: Supplementary file 18 [file peerj-14-21667-s020.pdf]

The Plant Awareness Inventory

[redacted]

Version 2.0

02/2025

This document contains the current version of the Plant Awareness Inventory. The following changes/additions have been made:

(1) Linguistic validation of the English version of the questionnaire
(2) minor changes in wording, addition of illustrative pictures
(3) freetext items were changed to closed items, based on the analysis of the freetext answers from the first version of the PAI
(4) One item has been removed due to complex analysis and to reduce the time for the questionnaire

A German version of the items can be received from the authors upon request [redacted]

Dimension: Attention

Take your time and take a close look at the hidden object picture! After 30 seconds, a ‘Next’ button will appear. Click on it when you are ready to proceed. Otherwise, you will be automatically redirected after 60 seconds.

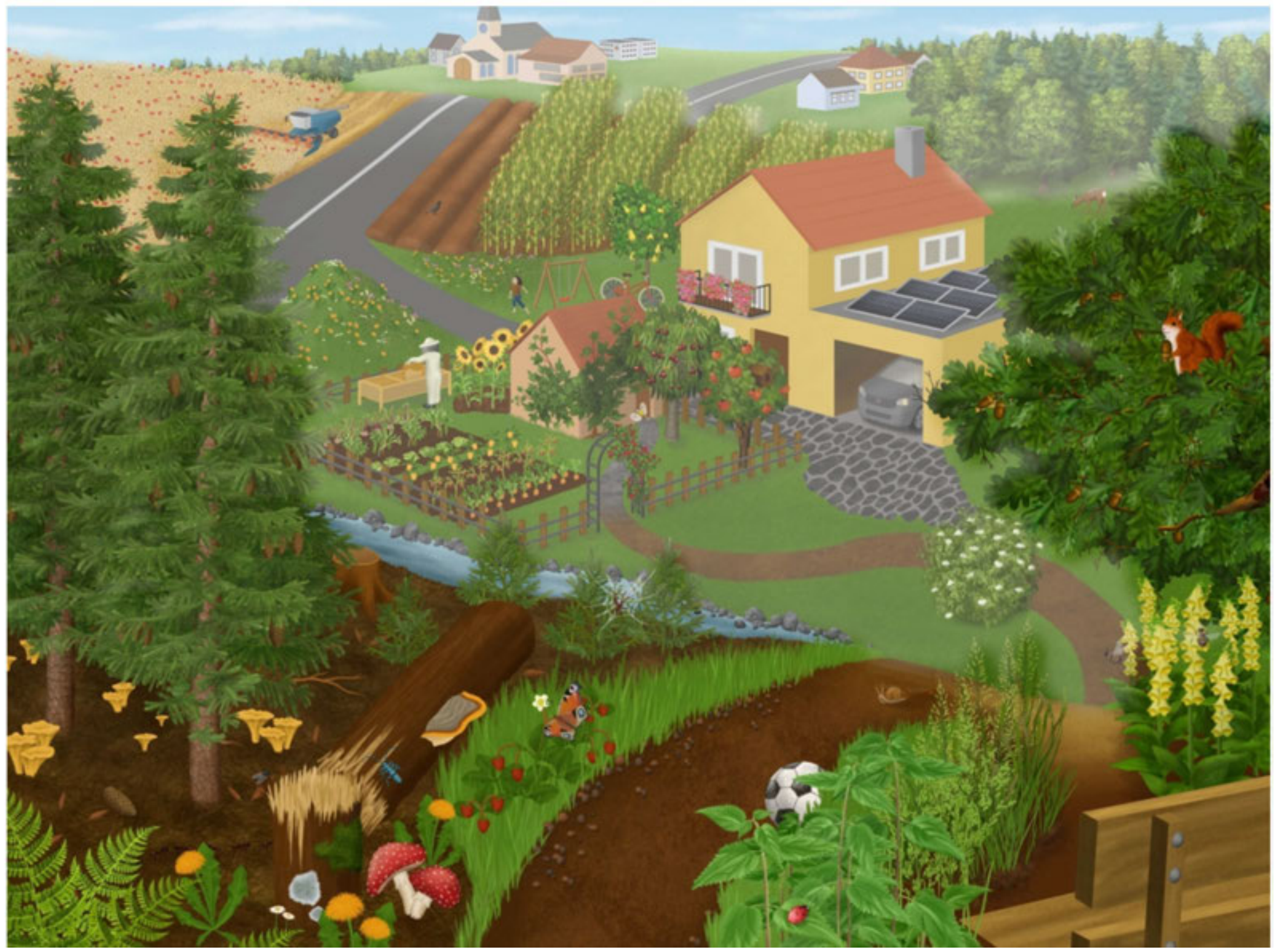

What did you see in the picture? Write down everything you can remember (you have space for a maximum of 25 words)!

When you have written an answer in a field and pressed the ENTER key, a new field automatically appears.

Dimension: Knowledge/Understanding

Which of these plants are often used as spices in the kitchen?
Tick the correct answers. Several are correct.

Subscale: spice plants

- ☐ Geranium
- ☐ Petunia
- ☐ Thuja
- ☐ Pepper
- ☐ Philodendron
- ☐ Basil
- ☐ Thyme
- ☐ Dandelion
- ☐ Oregano
- ☐ Marjoram

Subscale: Plant identification
Items of the subscale "Practical knowledge" are marked with an asterisk (*)

The plant in the picture is a...

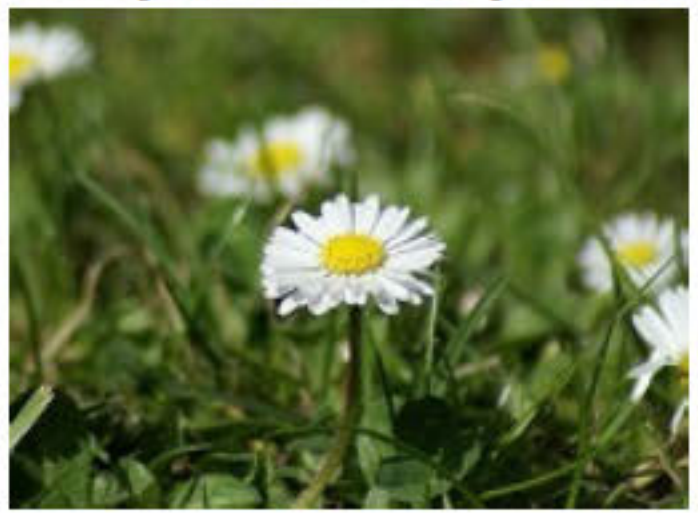

Tick the correct answer.

- ◯ Daisy
- ◯ Dandelion
- ◯ Clover
- ◯ Marguerite

Have you ever collected or picked this plant? (*)

Choose what applies to you.

Never	1 – 5 times per year	More than 5 times per year	(Almost) every time this plant flowers
◯	◯	◯	◯

Which of the following pictures shows a dandelion?

Tick the correct picture.

○ 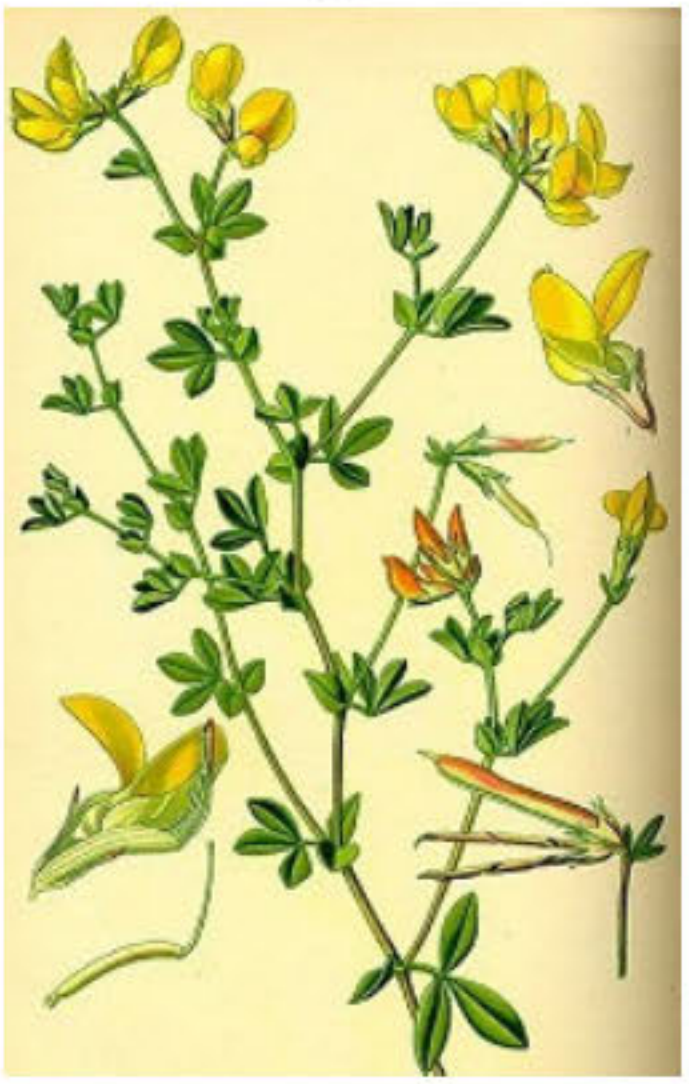

○ 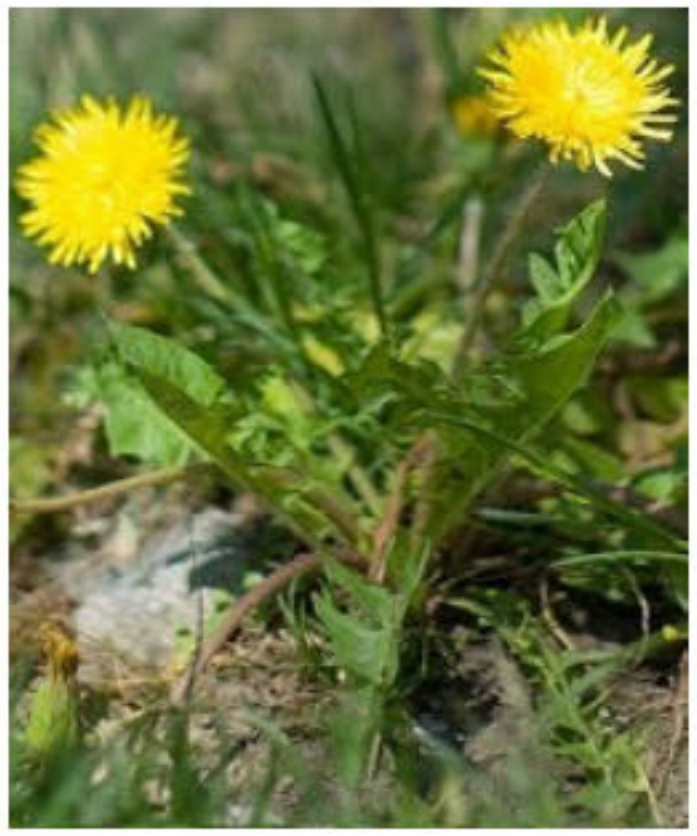

○ 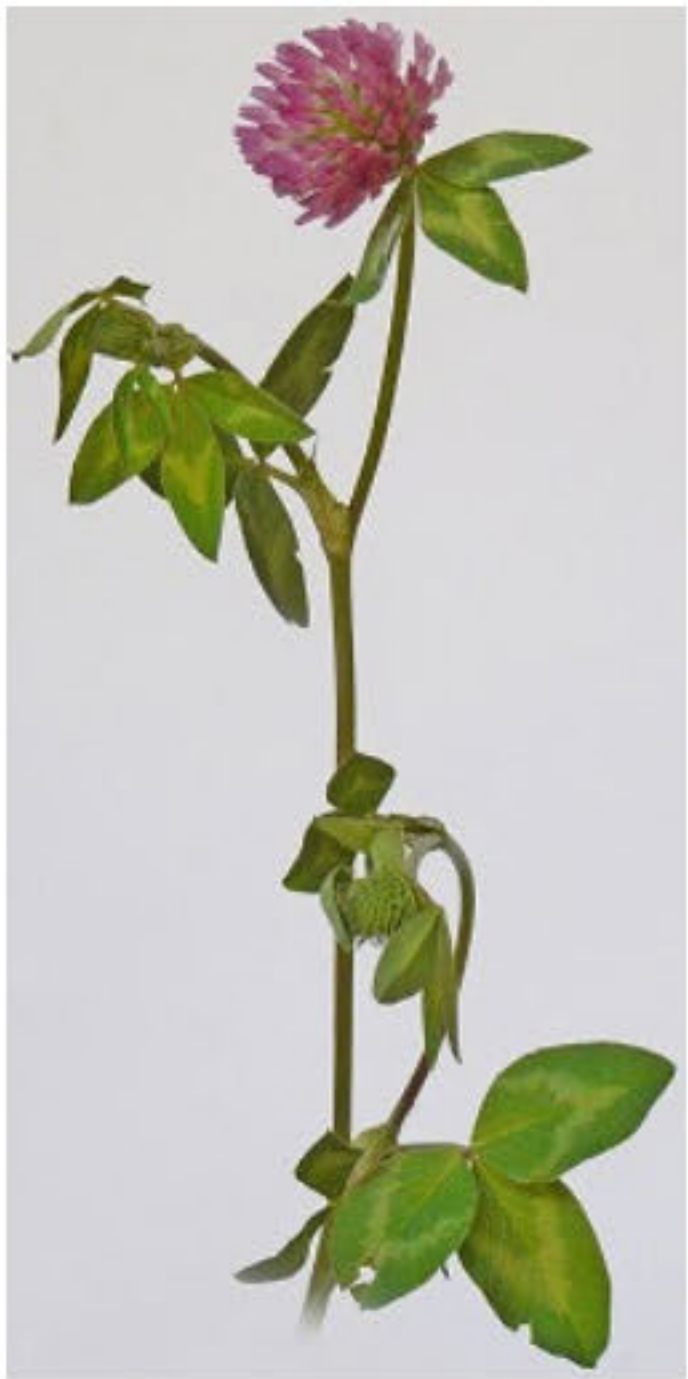

○ 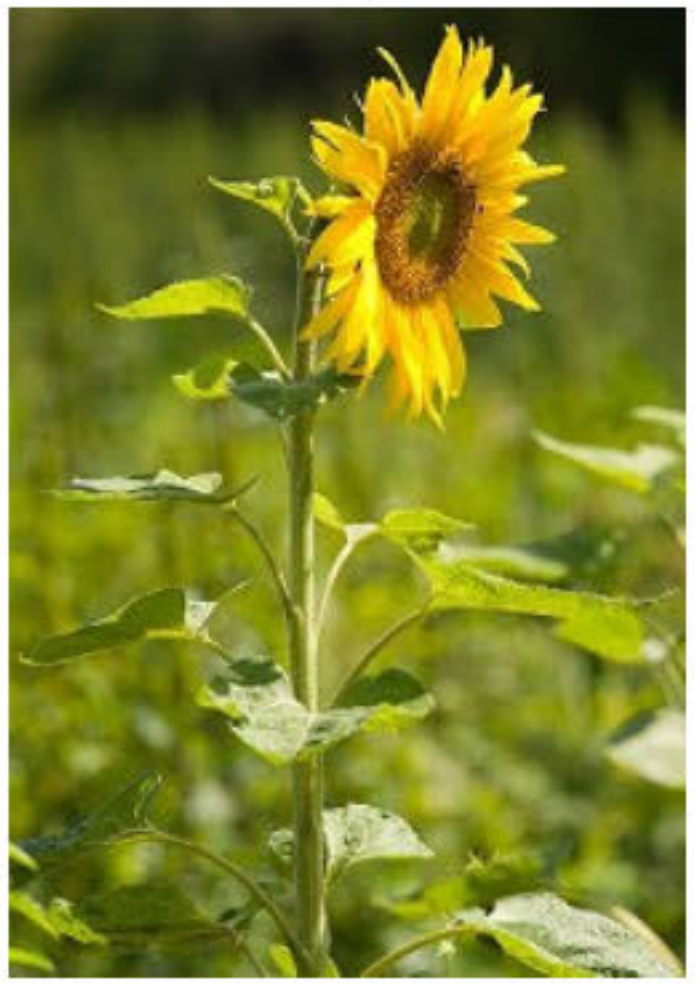

Have you ever collected or picked a dandelion? (*)

Choose what applies to you.

Never	1 – 5 times per year	more than 5 times per year	(Almost) every time this plant flowers
○	○	○	○

Which of these trees is a spruce?

Tick the correct picture.

○ 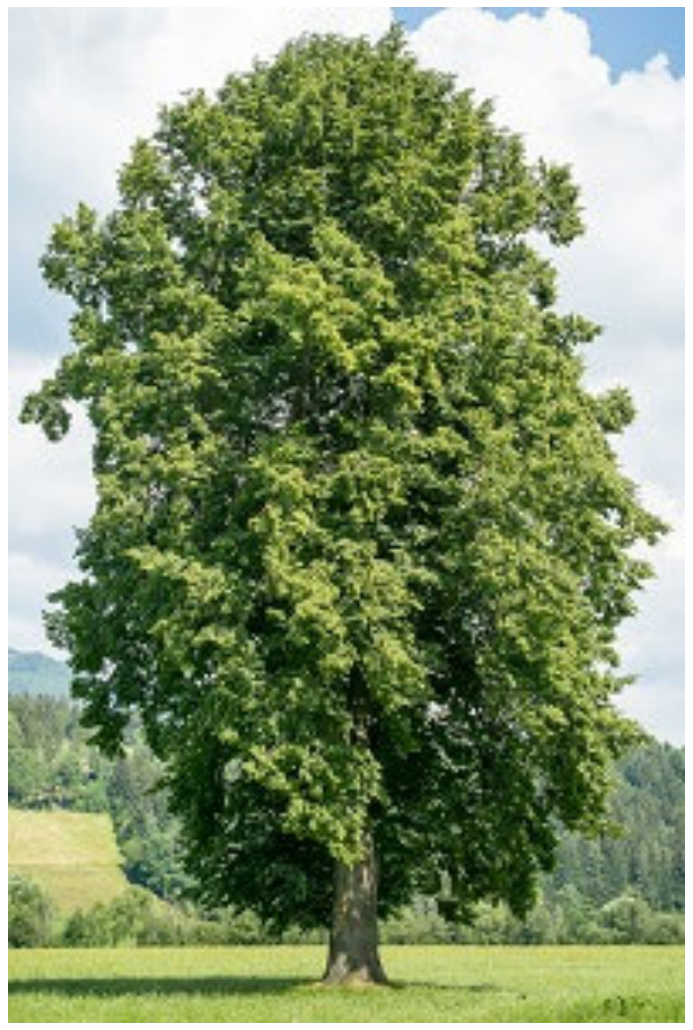

○ 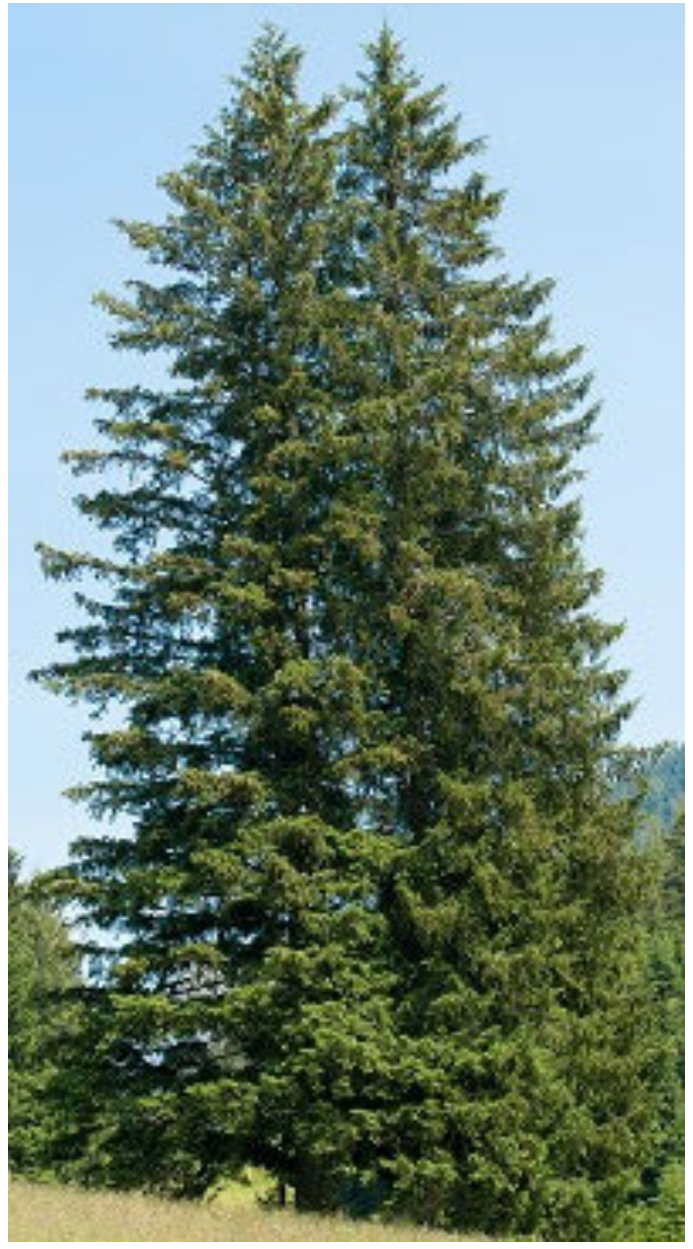

○ 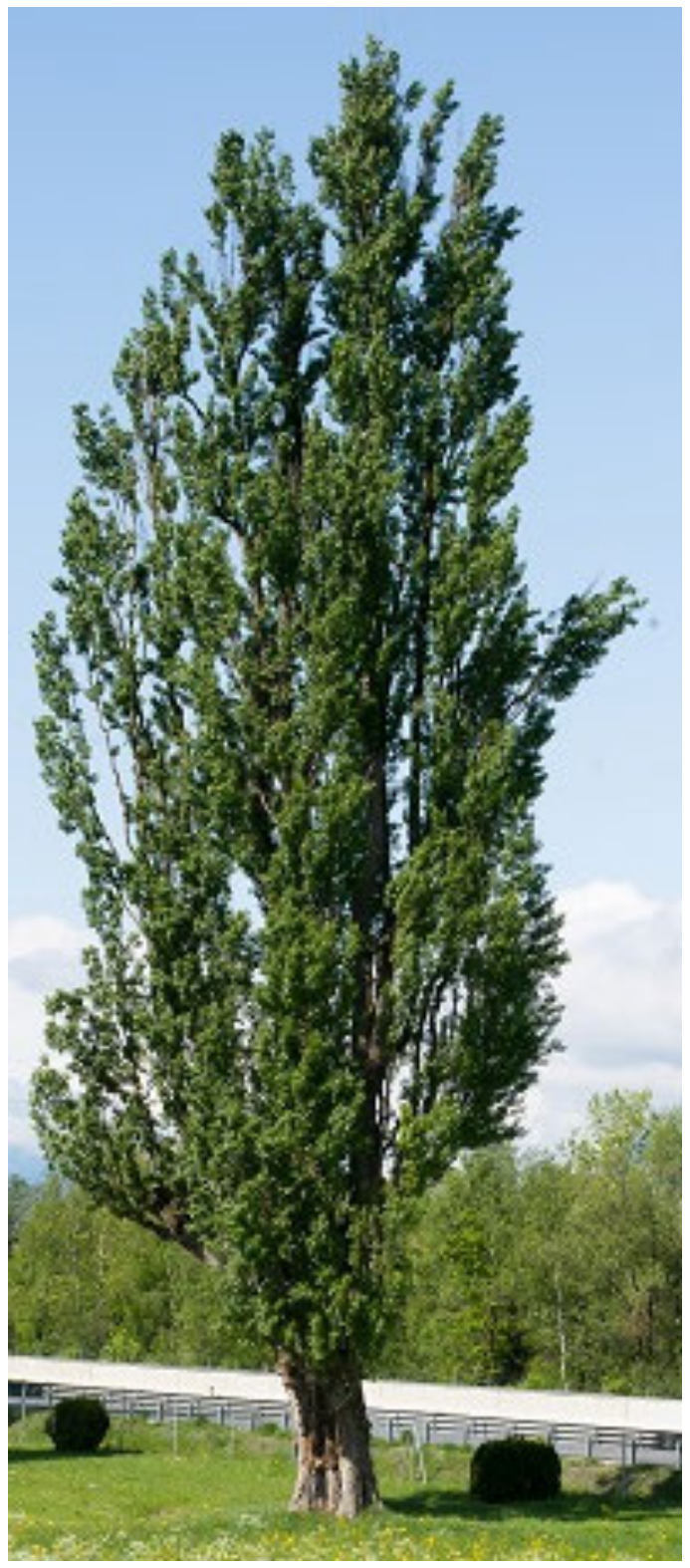

○ 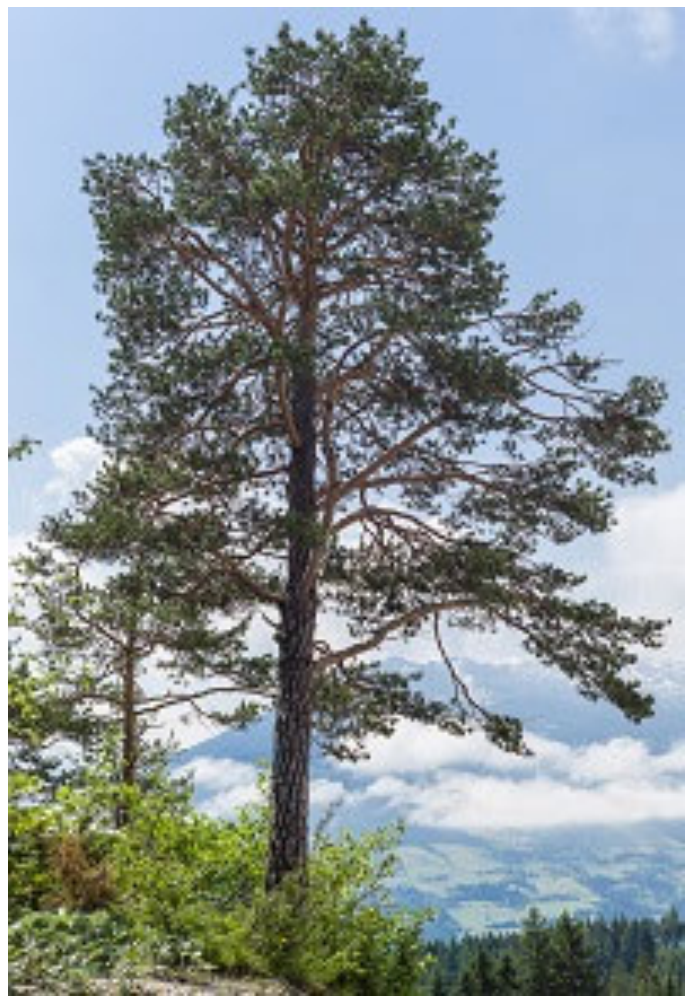

Which plant do you see in this picture?

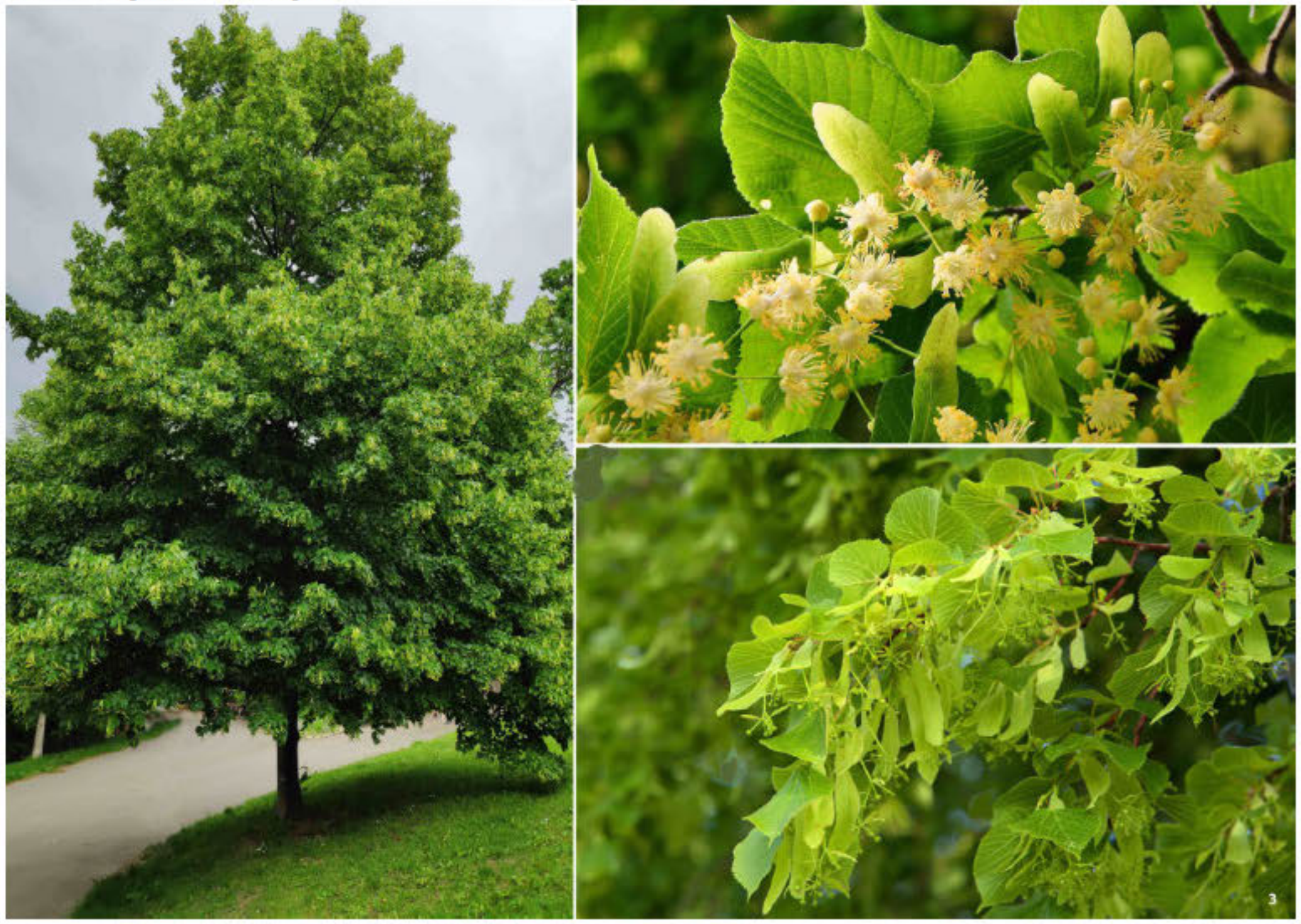

Tick the correct answer!

- ◯ Linden
- ◯ Maple
- ◯ Beech
- ◯ Elder
- ◯ Oak

Have you ever collected or picked parts of this plant? (*)

Choose what applies to you.

Never	1 – 5 times per year	More than 5 times per year	(Almost) every time this plant flowers
◯	◯	◯	◯

Which plant do you see in this picture?

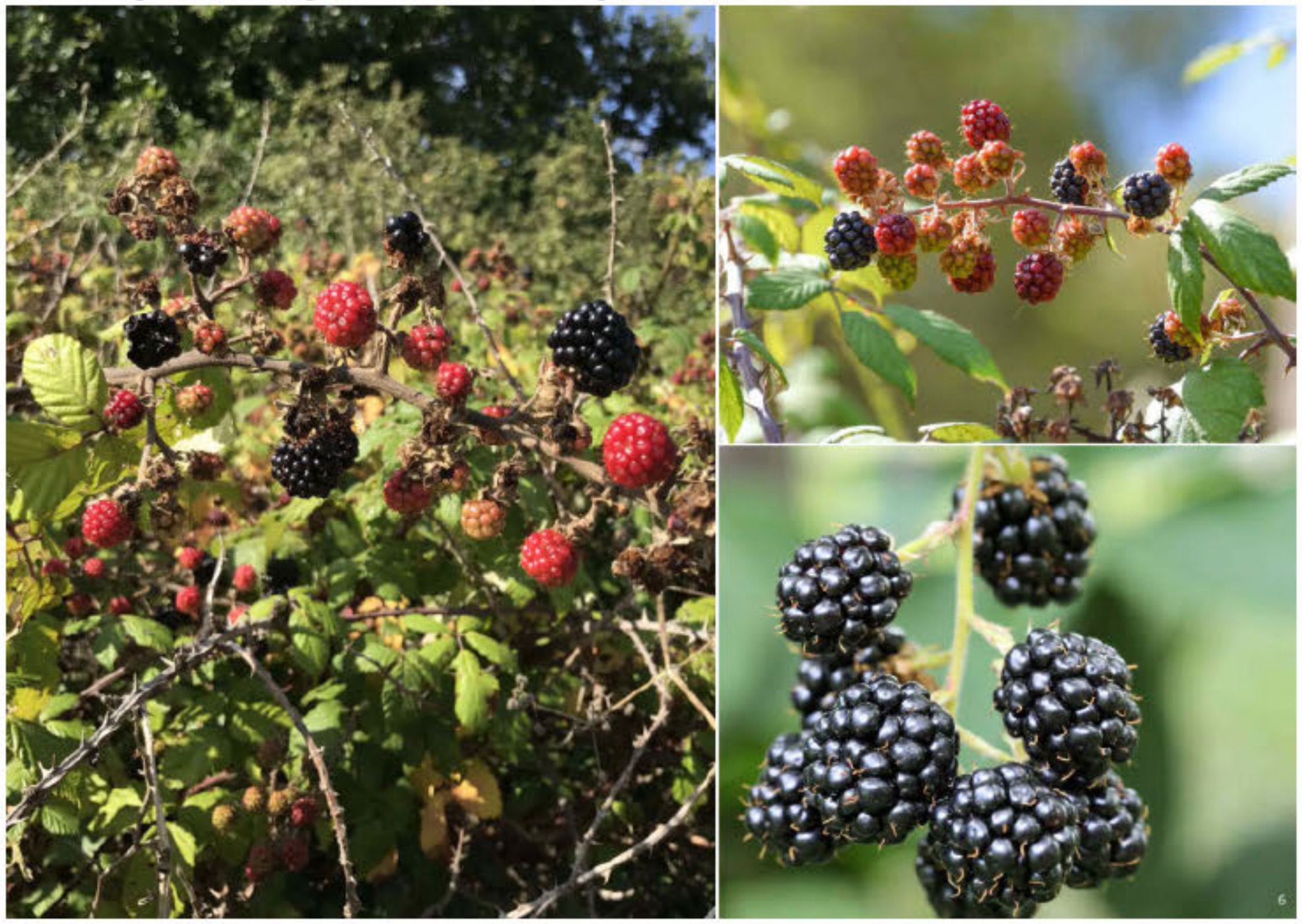

Tick the correct answer!

- ◯ Blackberry
- ◯ Blueberry
- ◯ Raspberry
- ◯ Currant
- ◯ Mulberry

Have you ever collected or picked the fruit of this plant? (*)

Choose what applies to you.

Never	1 – 5 times per year	More than 5 times per year	(Almost) every time this plant bears fruit
◯	◯	◯	◯

Decide for which of the groups listed – fungi, plants, animals – the respective statements apply.

Several groups can be selected per question. Choose the correct answer(s).

	Fungi	Plants	Animals	all three groups
Consist of one or more cells.	☐	☐	☐	☐
Are capable of growth.	☐	☐	☐	☐
Reproduce independently (multiply on their own).	☐	☐	☐	☐
They absorb nutrients, convert them and release substances that are not required.	☐	☐	☐	☐
React to their environment (are excitable).	☐	☐	☐	☐
Can move in different ways.	☐	☐	☐	☐
Pass on characteristics to their offspring.	☐	☐	☐	☐
Can become ill.	☐	☐	☐	☐

Which of the points listed directly influence the amount of greenhouse gases (e.g. carbon dioxide or methane) in the atmosphere?

Tick the correct answers. Multiple answers are possible.

- ☐ Air traffic
- ☐ Pasture farming (e.g. cows)
- ☐ Forests
- ☐ Solar activity
- ☐ Oil power stations
- ☐ Algae in the sea
- ☐ Ocean currents (e.g. the Gulf Stream)
- ☐ Car traffic

- ☐ I don’t understand the question.

Which of the following could reduce the production of plant-based foods in the future?

Tick the correct answers. Multiple answers are possible.

- ☐ Insect extinction
- ☐ Increased temperatures
- ☐ Extreme weather events
- ☐ Destruction of habitats
- ☐ Soil quality

- ☐ I don’t understand the question.

Which of the following items of clothing are made from renewable raw materials?

Tick the correct answers. Multiple answers are possible.

- ☐ T-shirt made from cotton
- ☐ Running shirt made of polyester
- ☐ Shirt made from linen
- ☐ Socks made from bamboo
- ☐ Trousers made from hemp

- ☐ I don't understand the question.

Which of the following energy sources are obtained from renewable raw materials?

Tick the correct answers. Multiple answers are possible.

- ☐ Wood
- ☐ Lignite
- ☐ Petroleum
- ☐ Natural gas
- ☐ Biofuel

- ☐ Ich verstehe die Frage nicht.

Dimension: Attitudes

When there are plants around me, I feel ....

Select the value that applies to you.

very unwell (bad) ○○○○○ very comfortable (good)

I find (most) plants ....

Select the value that applies to you.

not beautiful at all ○○○○○ very beautiful

In my family, plants play .... role at celebrations (e.g. as decoration).

Select the value that applies to you.

not a all (a) ○○○○○ a very important

I would like to have .... trees in my city.

Select the value that applies to you.

much less ○○○○○ much more

I would like to have .... plants in my immediate surroundings (e.g. at home or at school).

Select the value that applies to you.

much less ○○○○○ much more
